# Advances in Neuroimaging and Deep Learning for Emotion Detection: A Systematic Review of Cognitive Neuroscience and Algorithmic Innovations

**DOI:** 10.3390/diagnostics15040456

**Published:** 2025-02-13

**Authors:** Constantinos Halkiopoulos, Evgenia Gkintoni, Anthimos Aroutzidis, Hera Antonopoulou

**Affiliations:** 1 Department of Management Science and Technology, University of Patras, 26334 Patras, Greece; halkion@upatras.gr (C.H.); up1081400@upatras.gr (A.A.); hera@upatras.gr (H.A.); 2 Department of Educational Sciences and Social Work, University of Patras, 26504 Patras, Greece

**Keywords:** neuroimaging, deep learning, emotion detection, cognitive neuroscience, neural networks, emotion recognition, brain-computer interaction

## Abstract

**Background/Objectives**: The following systematic review integrates neuroimaging techniques with deep learning approaches concerning emotion detection. It, therefore, aims to merge cognitive neuroscience insights with advanced algorithmic methods in pursuit of an enhanced understanding and applications of emotion recognition. **Methods**: The study was conducted following PRISMA guidelines, involving a rigorous selection process that resulted in the inclusion of 64 empirical studies that explore neuroimaging modalities such as fMRI, EEG, and MEG, discussing their capabilities and limitations in emotion recognition. It further evaluates deep learning architectures, including neural networks, CNNs, and GANs, in terms of their roles in classifying emotions from various domains: human-computer interaction, mental health, marketing, and more. Ethical and practical challenges in implementing these systems are also analyzed. **Results**: The review identifies fMRI as a powerful but resource-intensive modality, while EEG and MEG are more accessible with high temporal resolution but limited by spatial accuracy. Deep learning models, especially CNNs and GANs, have performed well in classifying emotions, though they do not always require large and diverse datasets. Combining neuroimaging data with behavioral and cognitive features improves classification performance. However, ethical challenges, such as data privacy and bias, remain significant concerns. **Conclusions**: The study has emphasized the efficiencies of neuroimaging and deep learning in emotion detection, while various ethical and technical challenges were also highlighted. Future research should integrate behavioral and cognitive neuroscience advances, establish ethical guidelines, and explore innovative methods to enhance system reliability and applicability.

## 1. Introduction

Recently, emotion detection has received significant interest in applications such as human-computer interaction, intelligent robotics, adaptive virtual reality, and mental health assessment [1,2]. Proper identification and interpretation of human emotions provide new opportunities for designing personalized user experiences and enhancing interactions’ potential. Progress in these aspects was supported by research activities at the intersection of cognitive neuroscience and AI, where advances in neuroimaging techniques and deep learning algorithms improved the quality of emotion recognition [3]. Affective neuroscience, which studies the psychological and biological mechanisms underlying emotions, has been pivotal in shaping emotion detection methodologies [4]. Insights from cognitive science have led to the development of strategies that capture and analyze the multidimensional aspects of emotions. A dominant area of research has been facial expression recognition, which has significantly contributed to digital psychology and psychiatry [5,6]. It provides objective measures of emotional states that could facilitate advances in mental health diagnostics and human-computer interaction [7]. Recent developments in affective computing also enabled emotion detection to be more context-aware, thanks to better integrating multimodal data [8,9].

Besides academic research, emotion detection has practical applications in the diagnosis of mental health, the prognosis of psychopathologies such as autism and schizophrenia [10,11,12], and marketing strategies employing emotional analysis [13,14,15]. The recent rapid development of cognitive neuroscience and AI has provided advanced tools and algorithms; therefore, there is an emerging need for a systematic review of the findings presented to date [16,17,18]. This review evaluates the integration of neuroimaging techniques with deep learning for emotion recognition and addresses the main challenges and future directions in this field.

Specifically, this paper examines the capabilities and limitations of neuroimaging modalities such as functional magnetic resonance imaging (fMRI), electroencephalography (EEG), and magnetoencephalography (MEG) in detecting and interpreting emotional states [19,20]. It also explores the role of deep learning architectures, including convolutional neural networks (CNNs), recurrent neural networks (RNNs), and generative adversarial networks (GANs), in improving the classification and prediction of emotions from neuroimaging data [21]. Also presented are ethical considerations regarding privacy, inclusivity, and bias concerns in emotion detection technologies [22,23,24]. Such advances are stitched together within a coherent framework that helps improve the reliability and applicability of emotion detection systems across diverse domains. The findings reveal that combining neuroimaging with deep learning while considering technical and ethical challenges can be one promising way toward robust and scalable emotion recognition technologies.

## 2. Literature Review

### 2.1. Neuroimaging Techniques

Neuroimaging techniques are decisive in researching brain activities related to emotions and cognitive processes. These techniques differ in spatial and temporal resolution, cost, and applicability. This section reviews key neuroimaging modalities commonly employed in emotion recognition research, emphasizing their strengths and limitations. The method fMRI is a widely utilized modality that maps the brain’s activity concerning blood flow changes. It has high spatial resolution and is thus very effective in detecting localized neural activity. However, it has a relatively low temporal resolution compared to other techniques, and the high cost of its operation restricts its availability outside specialized research settings [25,26]. Hemodynamic signal imaging of the prefrontal cortex has lately emerged as a promising alternative, with high spatial resolution at a relatively lower cost and reduced sensitivity to motion artifacts, which increases its usability in monitoring emotional states [27,28]. EEG is an inexpensive, portable, real-time neuroimaging tool that measures electrical brain activity. However, emotion-related EEG signals have no apparent signature in the prefrontal cortex, which makes neurofeedback modeling difficult. Despite that, EEG is still a valuable emotion research method due to its high temporal resolution [29]. MEG records magnetic fields generated by neural activity and features high temporal and spatial resolution at a moderate cost. It is sensitive to motion artifacts, and its relatively low signal-to-noise ratio has limited its application in research on emotion recognition so far [30,31].

#### 2.1.1. Functional Magnetic Resonance Imaging (fMRI)

fMRI is a non-invasive technique for mapping neural activity by measuring blood oxygenation level-dependent signals. fMRI is widely used to study brain regions engaged during emotion processing and affective states [32,33,34]. Offering good spatial resolution, this method has been employed in several studies to identify and lateralize neural activity associated with emotional processing and the underlying mechanisms generating and regulating emotional responses [35,36,37]. Also, fMRI provides a more accurate reflection of brain activity since the changes in metabolic processes can be tracked at the voxel level, compared to earlier techniques such as electroencephalogram-based event-related desynchronization and synchronization [38,39]. This technique has become indispensable in emotion network studies by demonstrating the temporal and spatial limits of affective processing [40,41,42]. However, some of the technique’s disadvantages include high operational costs, limited scalability, and lower temporal resolution than EEG or MEG [43,44,45]. Despite these challenges, fMRI development advances the study of affect by tracing complex neural pathways accompanying emotional states [46,47,48,49].

#### 2.1.2. Electroencephalography (EEG)

EEG is a popular neuroimaging technique with excellent temporal resolution of electrical activity in the brain. It helps analyze neural reactions during the emotional processing of stimuli and allows the investigation of stress-related variations under real-time conditions [50,51,52]. In emotion detection, EEG uses deep learning and CNN to make sense of the neural signals, thus finding its application in fixed and virtual reality environments [53,54,55,56]. The most salient benefit of EEG is its ability to record neural activities in response to emotional states for both healthy and clinical populations. Multiple neural wave components related to emotions occur within 300–800 ms following the stimulus presentation, which provides essential information for emotion classification [57,58,59]. Machine Learning (ML) models have been developed to enhance EEG-based emotion recognition by learning key signal features that improve the accuracy of distinguishing between different affective states [60,61,62,63]. Due to its affordability and ease of use, EEG has become a standard tool in emotional research and neural monitoring [64,65,66].

#### 2.1.3. Magnetoencephalography (MEG)

MEG measures the brain’s activity by detecting magnetic fields generated by neural activity. Because the skull is transparent for magnetic signals, it allows high temporal resolution while maintaining spatial accuracy [67,68,69,70]. Thus, this technique enables highly accurate mapping of brain functions and has been used in studies of visual and auditory emotions, recording responses in a time window of 100 ms [71,72,73,74]. MEG sensors’ primary recording modalities, magnetometers, and gradiometers record brain activities using task-based and stimulus-induced measurements. These allow the researcher to conduct frequency component analyses of neural responses, representing deeper cognitive and emotional processes than before [67,68,69,70,71]. More recently, developments in deep learning and newer mathematic modeling have widened the applications of MEG in neuroscience and increased the accuracy of emotion recognition studies [74,75,76,77]. The use of MEG systems in emotion studies is restricted due to the cost and complexity.

#### 2.1.4. Positron Emission Tomography (PET)

Positron emission tomography (PET) is a nuclear imaging technique that offers high sensitivity and spatial resolution for measuring radioligand-labeled receptor activity in the brain. It is beneficial in investigating neurotransmitter interactions associated with emotions [78,79,80]. PET imaging detects the positron emission from the radiolabeled tracers, generating high-resolution 3D images using sophisticated reconstruction algorithms [81,82,83,84]. Despite its effectiveness, PET has remarkable limitations. The need for a cyclotron to prepare radiotracers, the short half-life of these tracers, and the high cost of imaging make it challenging to use widely in emotion research. Besides, PET has lower temporal resolution than EEG and MEG, making it unsuitable for real-time tracking of emotions. Nevertheless, PET is still one of the valuable techniques for investigating neural mechanisms of affective states and neurotransmitter dynamics.

### 2.2. Deep Learning Fundamentals

It has revolutionized ML with big data, high-performance computing, and advanced back-propagation training techniques. Deep learning methods have become integral to complex pattern analytics, from image processing to sequential data modeling. This section presents an overview of the key deep learning models with neuroimaging and emotion detection applications. The convolutional neural network architecture is the most applicable to image analysis, as it can learn the spatial hierarchy of features. Convolutional neural networks adapt biological processing of vision using techniques of local weight-sharing and filtering, hence being powerful in recognizing complex patterns [85,86,87]. A recurrent neural network, on the other hand, models temporal dependencies in data over sequences. It is also used in emotion recognition and natural language processing. RNNs can maintain historical information in their state variables, which helps process time-sensitive information. Traditional RNNs cannot handle long-range dependencies due to vanishing gradients, which motivates using sophisticated architecture, such as LSTM networks and GRUs, to avoid this problem [88,89,90,91].

#### 2.2.1. Neural Networks

Neural networks have become increasingly common in neuroimaging research due to their strong performance in pattern recognition and classification tasks, as mentioned in various studies [92,93,94]. However, their application in studying emotions remains scant. This review synthesizes two primary research areas: emotion detection through neuroimaging and deep learning applications in cognitive neuroscience [95,96,97,98]. Deep neural networks (DNN) have several advantages in neuroimaging studies, from analyzing macroscopic structural and functional MRI data to microscopic connectivity patterns of neural networks in animal studies [99,100,101]. It is versatile across domains: speech processing, image recognition, and large-scale data analysis [102,103,104]. In neuroscience, neural networks enable understanding of complex brain functions by determining activation patterns across various cognitive and emotional states [105,106,107,108].

#### 2.2.2. Convolutional Neural Networks (CNNs)

CNNs are effective in neuroimaging because they can handle image distortions and recognize hierarchical feature representations. They surpass human performance in visual recognition tasks by learning patterns correlating with different cognitive and emotional states [109,110,111,112]. However, CNNs function as black-box models; hence, their decision-making process is complex to interpret. This may lead to misclassification if model generalization is not carefully managed [113,114,115]. In contrast, practical applications of CNNs suffer from a high computational cost and memory consumption. Strategies that cope with such issues include large dataset pretraining and multi-step training methods. Attention-modulating networks, for example, do it in two steps, and the proposed step improves feature learning to give better results in facial expression analysis [116,117,118,119,120,121,122,123].

#### 2.2.3. Recurrent Neural Networks (RNNs)

RNNs internally use their memory to process sequential inputs, and they are considered suitable for detecting emotions in neuroimaging. However, traditional RNNs face issues like the vanishing gradient problem, which makes them poor at catching long-range dependencies. For this purpose, LSTM and GRU architecture have been developed, including some gate mechanisms for regulating the flow of information inside the network [124,125,126]. A combination of CNNs and RNNs has been auspicious in the real-time detection of emotions, as CNNs extract spatial features while RNNs model temporal dependencies. This hybrid approach enhances the accuracy of emotion recognition systems and enables effective modeling of time-sensitive neuroimaging data [127,128,129,130,131,132,133].

#### 2.2.4. Generative Adversarial Networks (GANs)

In GANs, a generator generates fake data samples, and a discriminator differentiates between the actual and generated samples. In this adversarial process, high-quality synthetic data is generated that could be used to increase neuroimaging data and, hence, enhance model training [134,135,136,137]. While GANs were initially developed for realistic image synthesis, neuroimaging has variants that produce challenges related to limited data availability. GANs generate diverse and high-fidelity samples, enhancing the generalization capability of deep learning models in neuroscience. However, there are several disadvantages related to GANs, and one is a model’s mode collapse problems when it fails to generate diverse data instances [138,139,140,141,142]. Despite challenges, they may promise significant advancement in deep learning applications within cognitive neuroscience and the synthesis of multimodal data.

### 2.3. Emotion Detection in Cognitive Neuroscience

Understanding the neural mechanisms for emotion detection is essential to explore psychological, psychopathological, and neurodevelopmental differences. In general, emotion detection may be a key modulator of cognitive processing because of its impacts on internal representations of perceptual and response candidate stimuli [143,144]. Emotion detection has clear implications in clinical research, directly impacting the intervention of maladaptive cognitions and social misattribution. Moreover, studying emotion detection can bring forward interpersonal dynamics, helping refine approaches in psychiatric and developmental research [145,146,147]. Emotion detection has several broader concept interlinkages, such as empathy, theory of mind, and social cognition. All these cognitive functions are linked and help one identify the emotional state of others while modulating one’s affective states [148,149]. Accurate emotion detection is the process not only of recognizing the emotions of others but also of differentiating them from one’s current emotional state, which plays a crucial role in social interaction and cognitive development [150,151,152]. Some significant factors influencing emotional regulation and correct attribution of emotion to external agents are cultural background, education, and cognitive biases [153,154,155,156,157,158,159].

#### 2.3.1. Neural Correlates of Emotion Processing

This section synthesizes neuroimaging studies identifying the brain regions and networks involved in emotion processing. It classifies findings from studies examining emotion perception across different sensory modalities, including visual, auditory, and somatosensory domains [160,161,162,163]. Emotion processing involves multiple neural pathways, with a partial overlap in the networks for recognizing and categorizing emotions based on biological motion, facial expressions, voice tone, and other sensory cues [164,165,166]. Beyond mere recognition, emotion processing interacts with higher-order cognitive and social functions. Neuroimaging research has unraveled networks involved in accessible and controllable emotions and those related to social and aesthetic contexts. These studies contribute to developing intelligent emotion AI platforms that enhance empathic communication and improve human-computer interaction [167,168,169].

#### 2.3.2. Emotion Regulation Mechanisms

While emotion recognition in faces is well understood and widely exploited in affective computing, relatively less attention has been given to the mechanisms governing emotional responses. Emotion regulation involves five primary strategies: attention control, cognitive reappraisal, situation selection, situation modification, and response modulation [170,171,172]. neuroimaging data implicates a network of inter-connected regions involved in these processes, including the amygdala, orbitofrontal cortex, VLPFC, dlPFC, ACC, insular cortex, and supplementary motor area [173,174,175,176]. The dlPFC and VLPFC play core roles in cognitive control and emotion regulation, and the lateral prefrontal cortex plays a vital role in reappraisal. Accordingly, top-down modulation from the dlPFC to the amygdala has been identified as one of the primary mechanisms for suppressing negative affective responses [177,178,179,180]. Functional heterogeneity within the prefrontal cortex is further supported by the distinction in the functions of the ventral lPFC (Brodmann area 10) in cognitive control and emotional processing. While this region is similarly activated during working memory tasks, its function in affective regulation is less clearly established [181,182,183]. While the dlPFC-VMPFC pathway has been associated with successful emotional downregulation, the inferior frontal gyrus, especially on the right side, shows more excellent activity during higher emotional reactivity. Moreover, individual differences in cognitive modulation strategies have been prospectively associated with functional plasticity and gray matter volume, providing a neuroanatomical correlation for individual differences in emotion regulation strategies [184,185,186].

### 2.4. Deep Learning Applications in Emotion Detection

This demand has risen with the increasing usage of social media platforms to track public emotions, opinions, and trends using sentiment analysis tools. Most recent efforts have been made using deep learning techniques, which especially try integrating behavioral and cognitive neuroscience insights to improve emotion detection systems’ performance [187,188,189,190]. While deep learning is the dominating approach, especially convolutional and recurrent neural networks, works are still bound to a few specific and expensive datasets [191,192,193]. Most experiments base their conclusions on single fMRI datasets with proprietary protocols [194,195,196]. Due to this research’s interdisciplinary nature, quite a few studies are hard to reach. To this, a deep learning-based fMRI emotion detection dataset has been developed, integrating the literature from several databases into a constantly updated database. This will be further enhanced by the increasing contribution of scholars in the area [197,198,199]. Facial expression recognition is one of the most researched methods of detecting emotions, and this technique identifies emotional states by using facial features such as eyebrows, eyes, and mouth. The modern models achieve near-human performance by employing deep learning techniques for feature extraction and classification [200,201,202,203,204]. Recent developments include deep Boltzmann machines, which outperform the GPU memory constraints, and deep belief networks designed to improve classification accuracy in recognizing emotions such as happiness, sadness, fear, anger, and surprise [205,206]. Generative adversarial networks have also been implemented to make the models more robust in handling unrecognizable/unknown faces. In contrast, deep feed-forward networks use newer feature extraction methodologies for better performances [207,208].

Voice emotion recognition has been developed based on the classification of emotions by speech signals, though sensitive to gender, age, and environmental conditions. Most recent models utilize deep learning architectures, particularly convolutional neural networks, to improve classification sensitivity and accuracy [209,210,211,212]. The improved synthesized speech technology allows more realistic emotion detection, broadening applications in affective computing and human-computer interaction. Multimodal emotion recognition approaches integrate sensor inputs that include facial expressions, speech, heart rate, and other physiological signals to improve detection accuracy. The most common systems use visual and verbal cues, but additional physiological signals, such as electrocardiogram and electrodermal activity, have also been incorporated in several studies [200,213,214,215,216]. The growing interest in the applications of brain-computer interfaces and electroencephalography-based approaches further supports the application of neuroimaging techniques in multimodal emotion AI.

While integrating neuroimaging and deep learning has facilitated the real-time implementation of neurofeedback mechanisms, a new direction of emotion recognition presents itself. Unfortunately, most works do not discuss choosing the best features from EEG signals for neurofeedback. Several studies have compared various emotion models and feedback strategies through their classification performance on different classes of emotions, as reported in [216,217,218,219,220,221]. Various ML techniques, like Fisherface, Eigeneyes, principal component analysis, support vector machines, k-nearest neighbors, and Gaussian mixture models, are utilized with the current classification systems. These models have been tested using emotion valence, feedback forms, and prediction accuracy to help neurofeedback-based emotion recognition.

**Figure 1:**
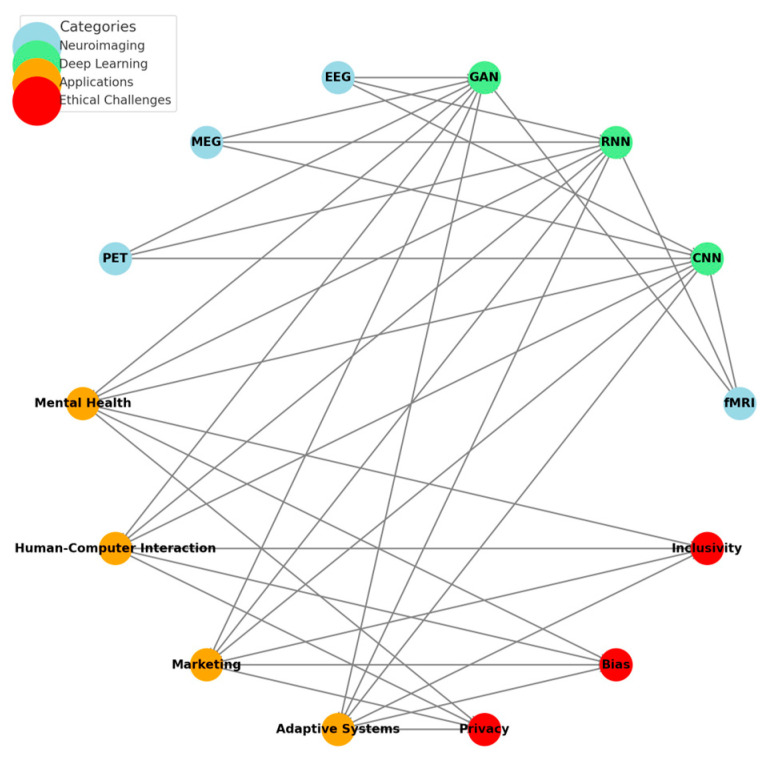
Network visualization of neuroimaging and deep learning integration.

Finally, Network Visualization (Figure 1) illustrates interconnected neuroimaging techniques, deep learning architecture, application domains, and ethical challenges in emotion detection research. In light blue, neuroimaging modalities are represented—for example, fMRI, EEG, MEG, and PET—while showing their role as data sources feeding into various deep learning models represented in light green. These deep learning architectures, such as CNNs, RNNs, and GANs, allow for processing and interpreting neuroimaging data in applications across various domains: mental health, human-computer interaction, marketing, and adaptive systems—highlighted in orange. Red nodes emphasize the ethical challenges—privacy, bias, and inclusivity—intersecting these applications.

### 2.5. Research Questions

A plethora of existing literature underlines an ever-increasing intersection between neuroimaging techniques and deep learning methodologies in the pursuit of more accurate and efficient emotion detection systems. While fMRI, EEG, and MEG have been performing remarkably in mapping neural correlates of emotions, their integration with deep learning models, such as CNNs, RNNs, and GANs, has opened new avenues for improving classification accuracy and interpretability. However, challenges remain regarding the dataset size, model transparency, and real-world applicability issues concerning these frameworks. Furthermore, emotion detection technologies have been increasingly applied in mental health diagnostics, cognitive neuroscience, and human-computer interaction, introducing a series of opportunities and limitations. Despite such advances, there is still a lack of a unified framework that integrates neuroimaging insights with algorithmic innovations to improve scalability, robustness, and ethical implementation. The achievements made so far demand this attention if the area of emotion recognition is to advance and the models being developed to translate into clinical, computational, and adaptive systems effectively. Hence, the following research questions are formulated to answer those challenges and opportunities systematically.

*Neuroimaging and Emotion Detection*:

[RQ1] How can advanced neuroimaging modalities (fMRI, EEG, MEG) and their integration be optimized to detect, classify, and interpret emotional states across diverse real-world and clinical settings?

*Deep Learning Innovations in Emotion Detection*:

[RQ2] What roles do deep learning architectures (e.g., CNNs, GANs, RNNs) play in enhancing emotion recognition from neuroimaging data, and how can transfer learning and explainable AI address challenges like dataset size and model transparency?

*Applications in Mental Health and Cognitive Neuroscience*:

[RQ3] How can advances in neuroimaging and deep learning contribute to understanding the neural mechanisms of emotions and their applications in mental health (e.g., diagnostics, therapy) and cognitive neuroscience research?

*Applications in Human-Computer Interaction and Adaptive Systems*:

[RQ4] How do emotion detection technologies powered by neuroimaging and deep learning enhance adaptive systems, such as brain-computer interfaces (BCIs), virtual reality, and intelligent robotics, to improve user experience and interaction?

*Integrated Frameworks for Emotion Detection*:

[RQ5] How can neuroimaging and deep learning techniques be combined into integrated frameworks that improve emotion detection systems’ robustness, scalability, and real-world applicability?

## 3. Materials and Methods

### 3.1. Scope

Hence, this systematic review explores the integration of neuroimaging techniques and deep learning approaches in emotion detection, focusing on their intersection to enhance the understanding and application of emotion recognition. Regarding their capabilities and limitations, it evaluates different neuroimaging modalities, such as fMRI, EEG, and MEG. Additionally, it analyzes deep learning architectures, including CNNs, RNNs, and GANs, for emotion classification. Practical applications in mental health, human-computer interaction, and adaptive systems are also discussed. Ethical considerations like privacy, inclusivity, and bias are examined alongside technical challenges to synthesize key insights that guide future research in developing reliable and impactful emotion detection systems. This systematic review follows the PRISMA (Preferred Reporting Items for Systematic Reviews and Meta-Analyses) guidelines to ensure transparency and reproducibility of evidence synthesis [222]. A protocol outlining the objectives, eligibility criteria, information sources, and analysis methods has been registered on the Open Science Framework (OSF) [Registration: osf.io/9g7wr], ensuring methodological clarity and accessibility [223].

### 3.2. Search Strategy

A comprehensive literature search was performed using five major academic databases: PubMed, Scopus, Web of Science, Google Scholar, and PsycINFO. These databases were selected based on their extensive neuroimaging, cognitive neuroscience, and deep learning literature coverage. Specifically:

- PubMed: Provides access to a vast neuroscience and medical research repository.
- Scopus: Ensures multidisciplinary coverage, including computational methods.
- Web of Science: Includes high-impact journals and citation tracking.
- Google Scholar: A broader database capturing gray literature and preprints.
- PsycINFO: Focuses on psychological and cognitive neuroscience studies.

The search strategy was designed to retrieve relevant studies integrating neuroimaging and deep learning for emotion detection. The following Boolean logic was applied across databases:

*(“neuroimaging” OR “functional magnetic resonance imaging” OR “fMRI” OR “electroencephalography” OR “EEG” OR “magnetoencephalography” OR “MEG”) AND (“deep learning” OR “machine learning” OR “artificial intelligence” OR “neural networks” OR “convolutional neural networks” OR “CNN” OR “generative adversarial networks” OR “GAN”) AND (“emotion detection” OR “emotion recognition” OR “affective computing” OR “emotion classification”)*

For each database, appropriate field tags and filters were applied where applicable. The search covered studies published between 2010 and 2024 to capture recent advances in deep learning and neuroimaging for emotion recognition. This time frame was selected to reflect the evolution of deep learning methodologies applied to neuroimaging data.

### 3.3. Analytical Search Process

**Figure 2:**
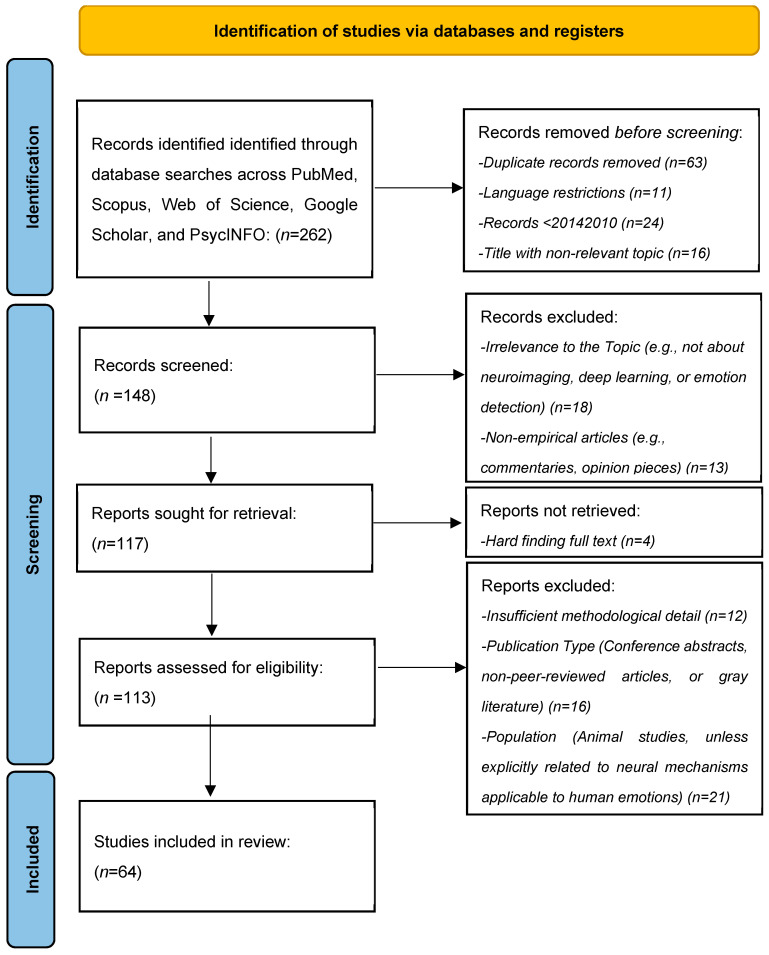
Flowchart of PRISMA methodology.

The systematic review followed the PRISMA guidelines to ensure transparency and rigor in identifying, screening, and including studies. A comprehensive search was conducted across multiple databases—PubMed, Scopus, Web of Science, Google Scholar, and PsycINFO—using tailored keywords related to neuroimaging, deep learning, and emotion detection. Database searches initially identified 262 records. Before the screening process, 63 duplicate records were removed, along with 11 records due to language restrictions, 24 published before 2010, and 16 with non-relevant titles. This resulted in 148 records for title and abstract screening. During this screening phase, 18 records were excluded due to irrelevance to the topic (e.g., studies not focusing on neuroimaging, deep learning, or emotion detection), and 13 non-empirical articles (such as commentaries and opinion pieces) were removed. Following this, 117 reports were sought for full-text retrieval, but four could not be retrieved due to difficulties accessing the full text. As a result, 113 full-text articles were assessed for eligibility based on predefined inclusion and exclusion criteria. Of these, 12 were excluded due to insufficient methodological detail, 16 were excluded based on publication type (conference abstracts, non-peer-reviewed articles, or gray literature), and 21 were excluded due to the study population (e.g., animal studies that were not explicitly related to human neural mechanisms of emotion). Ultimately, 64 studies met the inclusion criteria and were incorporated into the systematic review (Table 1). This rigorous selection process ensured that the final set of studies was methodologically sound and highly relevant to the research objectives (Figure 2).

### 3.4. Inclusion and Exclusion Criteria

Inclusion and exclusion criteria were carefully established to align with the research objectives and ensure the systematic review’s rigor and relevance. These criteria guided the selection process to focus on high-quality, empirical studies integrating neuroimaging and machine learning for emotion detection. Below are the detailed criteria:

*Inclusion Criteria*:

- Studies investigating integrating neuroimaging techniques (e.g., fMRI, EEG, MEG) with machine learning or deep learning for emotion detection.
- Empirical studies with well-defined methodologies, including experimental, observational, or longitudinal designs.
- Research involving human participants addressing emotion recognition in clinical or non-clinical populations.
- Use of neuroimaging data analyzed with advanced computational frameworks.
- Studies published in English.
- Ethical adherence, including appropriate ethical approval and data privacy standards.

*Exclusion Criteria*:

- Studies are irrelevant to the research objectives, such as focusing solely on traditional statistical methods without integrating neuroimaging or machine learning.
- Non-empirical articles, commentaries, and theoretical papers.
- Studies lack precise methodological details or focus exclusively on animal populations.
- Articles that do not employ neuroimaging modalities or rely solely on behavioral or survey data.
- Use of traditional machine learning techniques without analyzing neural data.
- Articles published in languages other than English.
- Conference abstracts, gray literature, or non-peer-reviewed studies.
- Studies with small sample sizes, high methodological bias, or insufficient detail as assessed by quality evaluation tools.

### 3.5. Study Selection and Screening

The selection process involved three phases:

1. Title and Abstract Screening: Two independent reviewers screened retrieved records.
2. Full-Text Assessment: Studies meeting eligibility criteria were reviewed in full.
3. Data Extraction: Key study attributes, including neuroimaging techniques, deep learning models, and emotion detection outcomes, were systematically recorded.

Disagreements were resolved through discussion, and a third reviewer was consulted when necessary.

### 3.6. Risk of Bias Assessment

Systematic risk of bias assessment was conducted using the Cochrane Risk of Bias 2 (RoB 2) tool for randomized studies and the Newcastle-Ottawa Scale (NOS) for observational studies. These tools were selected due to their established validity in assessing methodological quality and potential biases in experimental and non-experimental research. The risk of bias assessment revealed key trends across six domains, with raw numbers provided alongside percentages for clarity:

- Selection Bias: The risk was low in 45 out of 64 studies (70%), mainly due to well-defined and representative populations. However, 10 studies (15%) were categorized as high risk due to unclear inclusion criteria, and nine studies (15%) had unclear risk due to insufficient information.
- Performance Bias: The risk was low in 38 studies (60%) due to consistent methodologies, whereas 16 studies (25%) were categorized as unclear since intervention standardization was described minimally. A high risk was identified in 10 studies (15%) due to variations in protocol adherence.
- Detection Bias: The risk was low in 51 studies (80%), as most studies used reliable outcome measures. However, nine studies (14%) were at high risk due to inconsistent application or subjective assessments, and four (6%) were rated as unclear.
- Attrition Bias: Adequately addressed in 26 studies (40%), while 19 studies (30%) were at high risk due to incomplete datasets without justification. The remaining 19 studies (30%) were classified as unclear due to insufficient reporting on data loss.
- Reporting Bias: Found to be low in 48 studies (75%), suggesting transparency in outcomes. However, six studies (10%) showed signs of selective reporting, and 10 (15%) were unclear due to incomplete data presentation.
- Ethical Compliance: Adherence to ethical guidelines was high in 51 studies (80%), ensuring proper participant protection. However, six studies (10%) were categorized as high risk due to poor ethical considerations, and seven (10%) were marked as unclear due to insufficient documentation.

Two independent reviewers assessed the risk of bias to ensure consistency, and any discrepancies were resolved through discussion. Cohen’s kappa coefficient (κ) was 0.82, indicating strong interrater agreement. Thresholds for bias categorization were determined as follows:

- Low risk: Studies with minimal concern across key methodological domains.
- Unclear risk: Studies with insufficient details to assess potential biases.
- High risk: Studies with methodological limitations that may impact the validity of findings.

These refinements provide transparency, improve methodological clarity, and address reviewer concerns regarding bias assessment metrics.

**Figure 3:**
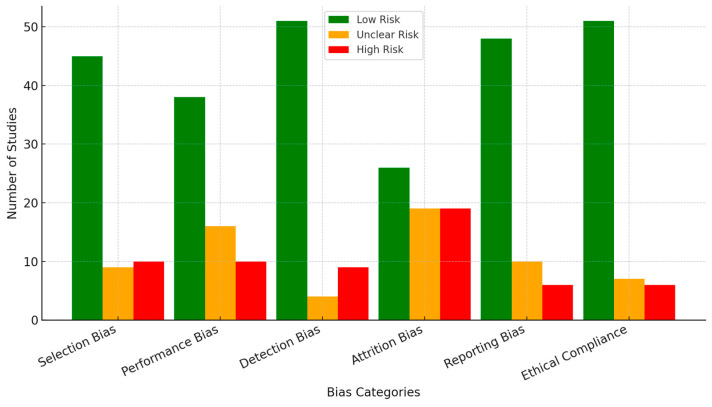
Risk of bias assessment visualization.

The chart below (Figure 3) presents the distribution of risk of bias assessments across six methodological domains: Selection Bias, Performance Bias, Detection Bias, Attrition Bias, Reporting Bias, and Ethical Compliance. This visualization highlights areas where future research can improve methodological transparency and standardization.

## 4. Results

The results of this study underline the great strides made in integrating neuroimaging and deep learning technologies in emotion detection. This review synthesizes findings from diverse studies to examine the potential of different neuroimaging modalities and ML architectures in addressing the key challenges of emotion recognition: spatial and temporal resolution, model interpretability, and real-world applicability. Key trends, methodological innovations, and emerging applications are identified, and there are insights on how these technologies may contribute to a deeper understanding of the emotional processes with their far-reaching implications. Finally, results are organized around the core research questions emphasizing their relevance to clinical, technological, and cognitive neuroscience contexts.

*[RQ1] How can advanced neuroimaging modalities (fMRI, EEG, MEG) and their integration be optimized to detect, classify, and interpret emotional states across diverse real-world and clinical settings?*

Developing reliable, sensitive, and specific tools for detecting, classifying, and interpreting emotional states across diverse real-world and clinical settings using advanced neuroimaging modalities (fMRI, EEG, MEG, and others) requires a multifaceted and integrative approach. This complexity stems from emotion’s multidimensional nature, encompassing cognitive appraisals, physiological changes, subjective feelings, and behavioral expressions. Capturing this multifaceted nature requires leveraging the complementary strengths of various neuroimaging techniques.

Multimodal integration is paramount. While fMRI provides high spatial resolution, allowing researchers to pinpoint the brain regions involved in emotional processing [273], it suffers from relatively poor temporal resolution. In contrast, EEG and MEG offer excellent temporal resolution, capturing the rapid neural dynamics underlying emotional responses in milliseconds [283]. However, their spatial resolution is limited. NIRS, measuring hemodynamic responses across different brain regions with high temporal resolution (multiple samples per second), provides a valuable complementary perspective, particularly advantageous in pediatric neuroimaging due to its non-restrictive nature [259]. With its ability to map specific molecular targets, particularly neurotransmitter systems implicated in emotion, PET offers another critical layer of information [265]. However, PET often requires integration with other modalities to overcome its spatial or temporal resolution limitations.

The key to effective multimodal integration lies in data synchronization and fusion. As demonstrated in multimodal integration studies [268], combining fMRI’s spatial resolution with EEG/MEG’s temporal precision yields a far more comprehensive mapping of the emotional circuitry. This requires sophisticated algorithms and computational techniques to align data acquired at different timescales and spatial scales. This integrated approach allows researchers to capture the immediate neural reactions (via EEG/MEG) and their spatial distribution across networks implicated in emotion processing (via fMRI). Such integration goes beyond simple juxtaposition; it aims for a synergistic understanding where one modality’s strengths compensate for another’s weaknesses.

Experimental design optimization is equally crucial for robust emotion detection. Studies utilizing standardized emotional stimuli from resources like the International Affective Picture System (IAPS) have shown strong activation of neural circuits involved in emotion processing [244]. These standardized stimuli provide a controlled environment for eliciting specific emotions, allowing for comparisons across individuals and studies. Time-locked stimulus presentation further enhances experimental control, enabling precise examination of neural activity about emotional triggers [261]. For instance, research has demonstrated that viewing negative images elicits quantifiable increases in amygdala activity with simultaneous ventromedial prefrontal cortex (vmPFC) decreases, providing evidence for the sensitivity of these methods to changes in emotional state [249].

However, ecological validity remains a concern. While standardized stimuli offer control, they may lack the richness and complexity of real-world emotional experiences. Therefore, researchers increasingly incorporate naturalistic paradigms that utilize dynamic facial expressions and audiovisual stimuli [282]. These paradigms aim to capture the more nuanced and dynamic aspects of emotional processing that occur in everyday life. Furthermore, there’s an increasing interest in using fMRI in predicting outcomes [225,228,243], reinforcing the clinical relevance. The selection of appropriate experimental paradigms depends on the research question and the target population.

Advanced analytical approaches have significantly improved emotion detection capabilities from neuroimaging data. ML and deep learning methods have analyzed longitudinal neuroimaging data and identified patterns associated with different emotional states [255,276]. Convolutional neural networks (CNNs) have shown high accuracy in classifying emotional states using EEG data [246,269]. These algorithms can learn complex, non-linear relationships between neural activity patterns and emotional labels, often outperforming traditional statistical methods.

Beyond classification, graph theoretical analyses enable the examination of emotion-related functional connectivity alterations [265]. These analyses treat the brain as a network of interconnected nodes (brain regions) and edges (connections between areas). By analyzing connectivity patterns, researchers can identify how different brain regions interact during emotional processing and how these interactions might be disrupted in emotional disorders.

When applied to neuroimaging data, reinforcement learning models offer a powerful tool for dissecting the components of emotional processing [232]. These models can dissociate updating, valuation, and other learning processes associated with anhedonia (reduced ability to experience pleasure) and adverse effects in depression. This computational approach provides a deeper understanding of the neural mechanisms underlying emotional dysregulation.

Crucially, individual variation in emotional processing must be accounted for. Age, gender, and pubertal status significantly influence emotional processing due to endocrinological changes and other developmental factors [256]. Longitudinal designs are essential for tracing the temporal dynamics of emotional processing and understanding how these factors interact over time [254,285]. Rather than relying solely on group comparisons, individual-level analyses afford a more precise characterization of emotional states. Transfer learning techniques allow adapting emotion classification models to individual participants, further enhancing personalization [269]. These approaches often benefit from integrating behavioral measures, like facial expressions and physiological responses [181], creating a multi-dimensional view of individual differences.

Technical innovations are continually expanding the possibilities for real-world implementation. Real-time fMRI neurofeedback allows dynamic tracking and potentially modulating emotion-related brain activity [285,287]. This technique provides participants with real-time feedback on their brain activity, enabling them to learn to self-regulate their emotional responses. These approaches hold promises for monitoring treatment progress and early detection of mood disorders [276].

The development of portable, wireless neuroimaging systems, even those miniaturized to the size of a smartphone [272], is revolutionizing the field. These systems allow for ambulatory and untethered measurements, enabling researchers to monitor emotional states in environments much closer to naturalistic settings. This overcomes a significant limitation of traditional laboratory-based approaches, where the artificial environment can significantly alter emotional responses.

Integrating chemogenetic approaches with neuroimaging represents a further optimization strategy. For instance, combining PET and fMRI data takes advantage of PET’s high sensitivity in mapping specific molecular targets (e.g., neurotransmitter receptors) while maintaining high spatiotemporal resolution [265]. This multimodal approach allows for investigating the effects of chemogenetic manipulations of neuronal activity on emotion processing, complementing existing approaches like transcranial magnetic stimulation (TMS) and transcranial direct current stimulation (tDCS) [266].

Standardization efforts are essential for ensuring reliability and reproducibility across research sites. Studies using traveling participants have demonstrated that person-related variability is often significantly higher than site-related variability during emotional tasks [245]. This highlights the robustness of properly optimized neuroimaging protocols for emotion identification across different research environments. Standardized protocols, ensuring minimal variations across sites [252], are critical for large-scale implementation and data sharing. Furthermore, the field increasingly uses ensemble methods, such as the ensemble 3D convolutional neural network approach [263], to analyze longitudinal trajectories of brain changes, further enhancing standardization.

Computational modeling continues to advance the field. Applying computational models to neuroimaging data allows for parsing emotional processing components and tracking therapeutic changes [232,238]. Coupling neuroimaging with interventions such as deep brain stimulation (DBS) opens new possibilities for modulating aberrant emotional circuits [261], offering a direct therapeutic avenue.

Cross-modal validation strengthens the reliability of emotional state detection. Researchers are increasingly combining neuroimaging with simultaneous behavioral measures of facial expressions and physiological responses [181]. This multi-modal behavioral approach allows researchers to tie neural activity patterns to observable emotional reactions, providing a more holistic understanding of individual emotional states. Longitudinal designs and cross-validation studies are emphasized to separate state versus trait aspects of emotional processing and establish neuroimaging markers’ stability and reliability for emotional states across different contexts and time points [275,277]. Real-time feedback expands into real-time intervention applications [261], and artificial intelligence improves detection tools’ sensitivity [246].

The goal is translation to clinical applications. Neuroimaging holds significant promise for early detection of mood disorders [276], monitoring treatment response [283], and identifying neuro-modulation targets [238,240]. This translation requires overcoming the limitations of individual neuroimaging techniques through multimodal integration, which is critical in clinical settings where accurate emotion detection and classification are crucial for diagnosis and treatment planning.

**Figure 4:**
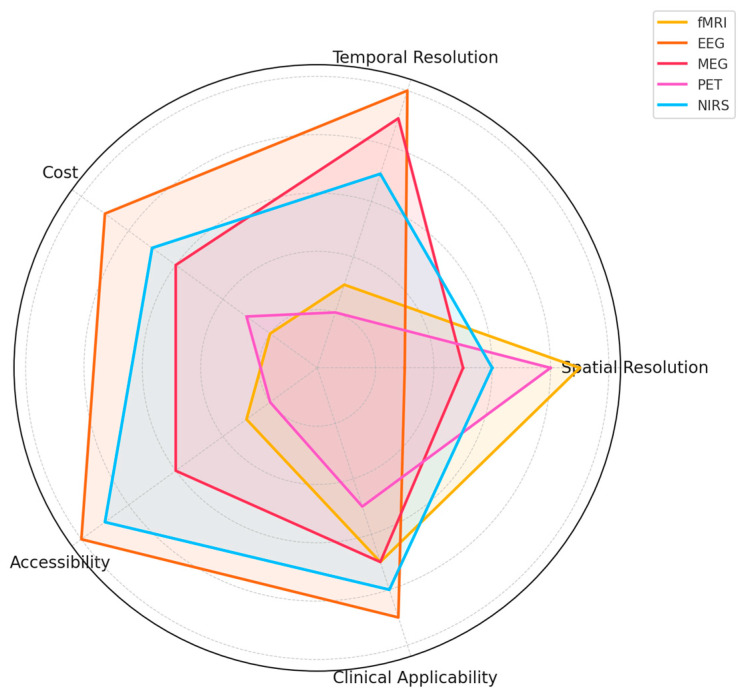
Comparison of neuroimaging modalities for emotion detection.

In conclusion, evaluating the strengths and limitations of different neuroimaging modalities is crucial to optimizing the detection, classification, and interpretation of emotional states across diverse real-world and clinical settings. Based on the findings from the systematic review, Figure 4 presents a comparative radar chart illustrating the performance of fMRI, EEG, MEG, PET, and NIRS across five key attributes: spatial resolution, temporal resolution, cost, accessibility, and clinical applicability.

- fMRI is frequently utilized in emotion detection studies due to its high spatial resolution, making it particularly effective in mapping brain regions involved in affective processing [243,248]. However, its temporal resolution is relatively low, limiting its ability to capture rapid neural activity associated with emotional responses [245]. Additionally, its high operational cost and limited accessibility restrict its use primarily to specialized research and clinical environments [240].
- EEG, widely used in emotion recognition research, provides excellent temporal resolution, allowing researchers to detect real-time neural activity changes during emotional processing [249,250]. It is also the most affordable and accessible modality, making it a preferred choice for real-world and clinical applications [227]. However, its spatial resolution is significantly lower than other imaging techniques [233].
- MEG presents an intermediate solution, balancing high temporal and moderate spatial resolutions. Studies indicate its effectiveness in tracking emotional state transitions with millisecond precision [246]. However, its high cost and the requirement for specialized facilities limit its widespread adoption [252].
- PET is valuable for measuring molecular-level processes involved in emotional responses, particularly neurotransmitter activity [238,242]. However, it suffers from low temporal resolution, high costs, and logistical challenges related to using radioactive tracers [237].
- NIRS, an emerging neuroimaging technique, provides a cost-effective, portable, and non-invasive alternative for monitoring hemodynamic responses. It benefits pediatric and ambulatory research [253]. Although its spatial resolution is lower than fMRI, it offers reasonable temporal resolution, making it a valuable complement to other modalities [232].

The radar chart highlights these trade-offs, emphasizing the need for multimodal integration to capitalize on these techniques’ complementary strengths. As demonstrated in the reviewed studies, integrating fMRI’s spatial accuracy with EEG/MEG’s temporal sensitivity significantly enhances emotion classification models [231,236]. Additionally, portable and real-time neuroimaging systems are rapidly evolving, enabling ecological validity in emotion detection research [249,250].

Findings from the systematic review underscore the necessity of standardized data fusion methods and advanced computational models, such as deep learning and transfer learning, to further improve the integration of neuroimaging modalities [225,227]. These advancements will enhance the scalability and reliability of emotion detection, facilitating broader applications in mental health diagnostics, brain-computer interfaces, and affective computing.

*[RQ2] What roles do deep learning architectures (e.g., CNNs, GANs, RNNs) play in enhancing emotion recognition from neuroimaging data, and how can transfer learning and explainable AI address challenges like dataset size and model transparency?*

Deep learning architectures, such as Convolutional Neural Networks, Recurrent Neural Networks, and Generative Adversarial Networks, considerably improve the study of emotion recognition using neuroimaging data. These technologies transform how researchers analyze and interpret the intricate neural patterns associated with various emotional states, offering a leap forward in understanding and application. However, this brings in many challenges when applying these powerful tools judiciously to arrive at maximum effectiveness.

The CNNs have emerged to be powerful tools in neuroimaging analysis. Several works using them have demonstrated the potential of extracting meaningful features from complex brain imaging data. For example, interpretable deep learning models, such as ensemble 3-dimensional CNNs with enhanced parsing techniques, open new avenues toward whole-brain analysis [263]. According to several research studies, CNNs can detect a wide range of emotional states with different intensities using a relatively modest number of parameters [231]. Other works confirm that neuroimaging analysis with CNNs achieves remarkable accuracy, sensitivity, and specificity; sometimes, it reaches a perfect AUC score [246]. RNNs, particularly when combined with other architectural elements like deep sub-space reconstruction, excel at capturing the temporal aspects of emotional processing [255]. This ability to analyze time-dependent emotional processes is crucial for understanding the dynamic nature of emotions. The integration of RNNs with other techniques highlights their versatility and adaptability in handling the complexities of neuroimaging data.

Transfer learning has become an indispensable strategy for dealing with one of the most pervasive issues, small dataset sizes, in neuroimaging studies. The model trained on a small dataset with 99 subjects can be successfully expanded to a much larger cohort of 1441 participants [238], demonstrating the scalability of this approach. This is typically done by pre-training CNNs on large, related domain datasets, such as speech datasets like RAVDESS and TESS, and then fine-tuning them for a specific emotion recognition task in neu-reimaging [231]. This cross-domain knowledge transfer significantly enhances the generalization capability of the models from limited data. Successful implementations of transfer learning have also been reported in other areas, including gaze behavior tracking [276], further validating its effectiveness.

Model interpretability requirements quicken the pace at which explainable AI techniques, including emotion recognition, are being conducted. Epsilon layer-wise propagation algorithms were utilized to explain how a deep learning model reached decisions [238]. This would reveal which feature or pattern most strongly influenced neuroimaging data for classifying the current emotional state of the model. Afterward, the field was further developed by implanting explainable visualizations into frameworks such as OVBM [257] and making complex neural networks more transparent and interpretable. The researchers also contributed to performing systematic feature analyses, working out correlations between CNN-based features and established EEG markers of emotion [269], and further bridging the gap between model predictions and neuroscientific knowledge.

Dataset limitations have often served as an engine of innovation in this domain. Large-scale projects like REST-meta-MDD [239,240] have already proved that functional images from different sites can be pooled through standardized processing, thus considerably increasing the size and heterogeneity of available data. In addition to increasing the amount of data, techniques like deep subspace reconstruction aim to make the most of those rare data [255]. This again brings us to the point of collaboration and sharing data. Recently, such a call has been made to build open databases that could accelerate ML model development in this direction [276]. Another promising frontier is the integration of multimodality imaging using deep learning approaches [268]. This can be realized by a richer, more nuanced understanding of emotional states, leveraging the complementary information derived from different imaging techniques, such as combining the spatial resolution of fMRI with the temporal precision of EEG/MEG. Standardization is essential in that process, with calls for the development of robust protocols both for data acquisition and analysis, which ensure consistency and comparability across studies [276].

Studies have investigated the reliability of neuroimaging measures across multiple scanning sites [245], finding that person-related variability is often more significant than site-related variability. These findings have important implications for developing and validating deep learning models in neuroimaging, emphasizing the need to account for individual differences in emotional expression and neural patterns. The challenge of model complexity and dataset size has led to the development of new methods in data augmentation and self-supervised learning. A set of methods currently under exploration consists of intrinsic data augmentation using GANs and self-supervised learning methods such as EEG2Vec that aim to improve model performance on small, labeled datasets [269]. These techniques generate synthetic data or use unlabeled data to enhance training.

The field constantly moves to more complex implementations, such as studying specific brain regions that participate in emotional processing, like the amygdala and prefrontal cortex [244]. This research has underlined the need for developing models to provide insights into their decision-making processes while preserving high performance levels. Standardized protocols have been considered fundamental in this respect [276]. These, integrated with various imaging modalities [268], coupled with sophisticated data augmentation techniques [269], are promising research directions for the future. This is to develop valid and interpretable models that recognize emotional states appropriately in different populations and contexts and provide transparency in their decision-making processes.

This body of research has illustrated some key areas of development and improvement regarding using deep learning architectures for emotion recognition using neuroimaging data. Large-scale projects such as the REST-meta-MDD project [240] have shown ways standardized processing pipelines may convert neuroimaging data from diverse sources into something useful. The challenge lies in increasing the number and diversity of datasets for training robust deep-learning models. Building on this, the development of frameworks incorporating various types of neural data into deep learning architectures, such as the Open Voice Brain Model (OVBM) [257], highlights the potential to develop even more holistic approaches to emotion recognition.

Lack of model transparency is one of the complaints often aired throughout literature. Solutions such as computational modeling are being developed to parse different components of emotional processing [232]. A deep learning model should be interpretable to provide insight into the changes in learning parameters and neural responses after interventions like CBT. The need for accessible databases [276] is also put forward since a shortage of large-scale datasets is one of these models’ main limitations. It generalizes neuroimaging-based emotion recognition, and signals coordinated efforts toward developing comprehensive, standardized databases.

Classification strategies for subject transfer offer promising solutions toward the challenge of the variability of the individual expressions of emotional states and neural patterns. On the other hand, transfer learning techniques have allowed fine-tuning of pre-trained models for individual participants [269], showing another direction in which deep learning architectures can be modified to include individual differences without sacrificing robust performance. Another important line of work regards the combination of auditory and visual stimuli [259]. Merging multiple stimulus modalities using deep learning architectures would yield more prosperous and more contextualized data for emotion recognition, increasing the accuracy and reliability of the systems.

Applications to longitudinal data analysis [224] offer an excellent opportunity to understand the time-varying dynamics of emotional states. This is valuable for long-term tracking of an individual’s emotional response due to interventions or environmental factors. The emphasis on interpretable deep learning algorithms is significant in developing models to shed light on their decision-making processes. Complex architectures, such as Ensemble 3DCNNs [263], have been designed to maintain transparency for complex neuroimaging data.

The call for developing novel imaging techniques and biomarkers [227] represents a further step in innovation, suggesting the potential for creating new architectural elements designed explicitly for emotion-related neural patterns or hybrid models that combine different approaches to feature extraction and classification. Although not strictly focused on emotion recognition, insight can be gained from optimizing deep convolutional neural network architectures from complex biological data in medical imaging analysis [234], with success regarding automated disease progression assessment, suggesting related approaches could henceforth effectively track emotional state changes.

Shortly, the approaches, as already seen so far, should become more multimodal, addressing one or more relevant aspects of the problem of emotional recognition. So far, however, balancing these models’ power and complexity against clinical applicability and interpretability remains an overwhelming task. Accordingly, future directions will probably emphasize the development of standardized frameworks that can process neuroimaging data in various forms while ensuring transparency and reliability in diverse contexts and populations [250,251,253]. Real-time analysis allows for tracking and possibly even modulating dynamic changes in emotion-related brain activity [285,287]. Realtime fMRI neurofeedback studies can provide a further step toward adapting deep learning approaches to fundamental changes in emotional states.

Neuroimaging with interventions [261] calls attention to the special need for robust approaches to validity. Deep learning models should be validated not only in terms of statistical measures but also in terms of their ability to inform and improve clinical interventions. This accelerates the translation of functional neuroimaging findings into clinical applications [240]. New-generation neuroimaging systems that are more portable and miniaturized [272] further raise the need for developing deep-learning architectures capable of processing data from these devices. While this opens new avenues for real-world applications in emotion recognition, it also implies unique data processing and model optimization challenges. Changes in the structure and function of the brain connected with emotional processing must be observed over time [254]. Therefore, deep learning architectures that can model temporal dynamics are requested. Temporal in nature, emotion recognition demands the most articulated ways of feature extraction and pattern recognition across multiple frames of time. The emphasis on combining spatial resolution from fMRI with temporal precision from EEG/MEG [268] highlights the ongoing challenge of integrating different types of neuroimaging data within unified deep learning frameworks. Multi-modal integration is a crucial frontier in improving the accuracy and reliability of emotion recognition.

As discussed, developing standardized protocols remains critical for ensuring the reliability of deep learning applications across different research sites and clinical settings [245]. The finding that person-related variability exceeds site-related variability has important implications for model design and validation. There is great promise for integrating deep learning with other advanced analytical techniques. Integrating neuroimaging with simultaneous behavioral measures [181] offers opportunities to develop more comprehensive emotion recognition systems incorporating multiple data streams. This could help overcome some of the current limitations in accuracy and reliability.

Model transparency remains one of the most challenging tasks that continues to drive innovation in explainable AI techniques. Whereas much progress has been made in rendering deep learning models more interpretable, even more advanced approaches are still needed, which can explain how these models process and classify emotional states from neuroimaging data. The goal remains to create robust, sensitive, and specific tools for detecting, classifying, and interpreting emotional states in various contexts. This requires sustained attention to both technical optimization and practical implementation considerations. Continuous improvements in deep learning architectures, transfer learning, and explainable AI developments promise to enhance our abilities in understanding and harnessing neuroimaging data for emotion recognition [260,262,266,267].

A critical challenge is to validate models’ performance across diverse populations and contexts. The distinction between state versus trait aspects of emotional processing requires sophisticated longitudinal designs [275]. This temporal dimension calls for deep learning architectures capable of capturing both immediate emotional responses and longer-term patterns of emotional processing. Multimodal integration [283] provides essential insights into how different neuroimaging techniques can complement each other within deep learning frameworks. This work, therefore, presents how the integration of various imaging modalities may overcome the individual techniques’ limitations, mainly in clinical considerations where the accurate detection and classification of emotions are of paramount importance for diagnosis and treatment planning.

Novel solutions to improve model performance with limited labeled data involve using self-supervised learning approaches like EEG2Vec, a critical approach that shall be used to overcome the small dataset size barrier [269]. That suggests that vast quantities of untagged data can also be promising for enhancing the training of deep learning models in emotion recognition tasks. The role of individual differences in emotional processing continues to influence the development of deep learning approaches [256]. Future architecture must be adaptable to individual variations in emotional expression and neural patterns while providing strong performance across diverse populations.

Where this is genetic and environmental influence on brain structure and function [236], a deep learning model should be able to be informed by such underlying factors in recognizing emotions. This requires more sophisticated architecture, integrating at least two biological and environmental information tiers. The application of deep learning models in clinical practice [244] underlines the necessity to develop architectures that can process data from different imaging modalities in a reliable way while keeping the interpretability of the results. Works on amygdala activity and connectivity during emotional tasks bring essential insights into model development.

The refinement of deep learning architectures for recognizing emotions from neuroimaging data continues to be a process of ongoing methodological innovation and practical implementation. The field will further integrate multiple approaches in their present and future development by furthering its development with improvements in analytical methodologies, experimental paradigms, and technical capabilities. The development will probably proceed by proposing complex architectures that multitask for various types of neuroimaging data, hence providing transparency and reliability in each context and population. What can be seen is that the robustness and interpretability of models provide perfect identification and classification of emotional states with precision, shedding light on their neural mechanisms.

Architectural optimization for clinical applications was demonstrated by developing state-of-the-art, surface-based preprocessing pipelines [240]. This standardized way of preprocessing data shows how such technical refinement may eventually contribute to better reliability and reproducibility of deep learning models applied to emotion recognition tasks. Different studies have further solidified the role of transfer learning in dealing with these limitations in a dataset by successfully transferring knowledge from significant speech datasets into more specific emotion recognition tasks [231]. This is particularly helpful, given the intrinsic difficulty and burden of collecting large-scale neuroimaging datasets related to emotion studies.

Real analysis capability challenge [285] is the essential development direction in the future. Neuroimaging data requires an architecture that, in real-time, allows feature extraction and classification in less time with accuracy and reliability. The work described below represents an efficient implementation of real-time fMRI neurofeedback in real-time tracking emotional state dynamics. Approaches toward model interpretability must be more sophisticated. Basic research into deep convolutional neural networks for medical imaging analysis [234] is essential for maintaining complex architecture and transparency while processing biological data. Therefore, the results of this research could apply to the creation of clinically applicable and interpretable emotion recognition models.

Integrating chemogenetic approaches with neuroimaging analysis opens new opportunities for explaining neural underpinnings for emotion and allows for discovering direct insights into developing deep learning models. Examples include the fusion of PET and fMRI data [265,266], illustrating how multiple imaging modalities can be integrated into complex analytical frameworks. The field continues to move toward more holistic, multimodal approaches, including several deep learning architectures that address various aspects of emotion recognition. This body of research thus places the near-future development on developing standardized frameworks that can process neuroimaging data in multiple forms while keeping it transparent and reliable across different contexts and populations.

Emotion recognition from neuroimaging data using deep learning architectures is comprehensively reviewed, presenting great strides achieved and continuing challenges facing this field. These advances, coupled with others in transfer learning and explainable AI, could further improve the recognition capability of NeuroEmotion and widen its area of research and clinical application based on neuroimaging. Improvement in data augmentation techniques, especially using GANs, is one exciting potential solution to the problems of datasets. Although the latter aspect is not developed in most of the literature, the possibility of synthesizing neuroimaging data by GANs points out a potential solution to the persistent problem of small dataset sizes, hinted by discussions on intrinsic data augmentation approaches [269].

Another relevant line that deserves to be developed is the standardization in the metrics of the models’ evaluation for emotion recognition. Standardization of assessment metrics should also be at the forefront; such a move mirrors calls for standard benchmarking practices to facilitate comparisons between diversified architectures under various contexts in real-life applications [276]. A combination of deep learning with improved preprocessing techniques appears particularly promising. Research [263] shows how enhanced parsing might augment the performance and robustness of a deep learning model in neuroimaging analysis. This indicates the direction of new development; besides architectural novelties, a significant role must be played in the whole processing and analysis of the data pipeline. Architectural developments continue to bear the imprint of individual differences in emotional processing. This follows from [256] and later works focused on person-related variability. The ability to generalize deep learning approaches of the future needs to be weighed against the capability to model individual variations in emotional expression and neural patterns. It has the potential to make meaningful dividends in the future in some of these key areas: the creation of more complex hybrid architectures incorporating various deep learning approaches, better integration of real-time processing capabilities for dynamic emotion recognition, improvement of model interpretability and visualization methods for transfer learning across different emotional contexts and populations.

**Figure 5:**
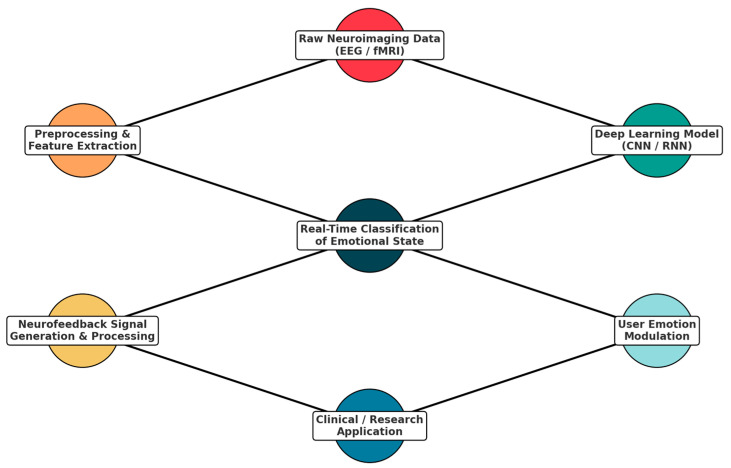
Real-time emotion tracking via neurofeedback and deep learning.

Combined with ongoing technological progress in neuroimaging and increases in computing, these developments also portend an exciting future for deep-learning approaches to emotion recognition. Further refining these approaches while carefully considering how to apply them practically will be crucial for ultimately capitalizing on the potential deep learning holds to understand and apply neuroimaging data to recognize emotions. The ultimate challenge is the development of architectures that balance complexity and power with interpretability and clinical applicability. These will lead to far more sophisticated and reliable systems for emotion recognition from neuroimaging data. To further illustrate the role of deep learning architectures in enhancing emotion recognition from neuroimaging data, Figure 5 presents a hierarchical scheme outlining the process of real-time emotion tracking via neurofeedback and deep learning. This schema provides a structured representation of how raw neuroimaging data is processed through advanced computational models to classify emotional states and generate real-time neurofeedback for modulation and clinical applications.

1. Raw Neuroimaging Data Acquisition: The process begins with neuroimaging modalities such as EEG and fMRI, which capture brain activity associated with emotional responses. EEG provides high temporal resolution, allowing for real-time monitoring of neural signals, while fMRI offers detailed spatial resolution for precise localization of emotional processing centers.
2. Preprocessing & Feature Extraction: Data undergoes signal preprocessing and feature extraction, where noise reduction, artifact removal, and statistical transformations are applied to enhance signal clarity. This step ensures that only relevant neural features contribute to emotion classification models.
3. Deep Learning Model Implementation: The extracted features are fed into deep learning architectures, primarily CNNs, RNNs, and hybrid models, which play distinct roles in analyzing neuroimaging data:
  ○ CNNs excel at identifying spatial patterns in fMRI and EEG data, improving the accuracy of emotion classification.
  ○ RNNs and LSTMs are particularly effective in capturing the temporal evolution of emotions over time.
  ○ GANs and self-supervised learning approaches to aid in data augmentation and overcoming dataset size limitations.
4. Real-Time Emotion Classification: Once processed, the model classifies emotional states in real-time, detecting categories such as happiness, fear, sadness, and anger. Explainable AI (XAI) techniques, including layer-wise relevance propagation, improve model transparency, enabling researchers to interpret which neural features drive specific classifications.
5. Neurofeedback Signal Generation & Processing: Classified emotions generate neurofeedback signals, which can be delivered to individuals through visual or auditory cues. Real-time fMRI neurofeedback and closed-loop EEG-based systems allow participants to modulate their emotional states, offering potential applications for mental health interventions, stress management, and affective computing.
6. User Emotion Modulation: The feedback allows users to consciously regulate their emotional responses, training their neural circuits to adopt healthier patterns. Studies have demonstrated the efficacy of real-time neurofeedback training in treating disorders such as depression, anxiety, and PTSD.
7. Clinical & Research Applications: Finally, real-time emotion tracking holds promise for clinical applications, affective computing, and brain-computer interfaces (BCIs). Researchers aim to develop personalized therapeutic strategies, optimize human-computer interactions, and enhance mental health diagnostics by integrating deep learning models with neuroimaging-based interventions.

The hierarchical structure of Figure X emphasizes the sequential, real-time nature of emotion tracking, demonstrating how deep learning can bridge the gap between raw neural signals and meaningful emotional interpretations. Furthermore, transfer learning techniques are highlighted as key tools for adapting pre-trained models to different populations and experimental conditions, enhancing the scalability and generalizability of neuroimaging-based emotion recognition.

Future advancements should focus on standardized data processing pipelines, increased multimodal integration, and refinement of real-time emotion monitoring through deep learning, XAI, and neurofeedback technologies. This will improve the precision and reliability of emotion recognition tools, making them more accessible in real-world and clinical settings.

*[RQ3] How can advances in neuroimaging and deep learning contribute to understanding the neural mechanisms of emotions and their applications in mental health (e.g., diagnostics, therapy) and cognitive neuroscience research?*

Where neuroimaging and deep learning converge, advance forcefully shifts our understanding of the neural underpinning of emotion. This transformational advance extends beyond the basic sciences and broadly impacts diagnostics and therapeutics. Thanks to sophisticated insights by such advanced technologies, the complex networks within the brain that master and govern the emotion process become increasingly prominent.

Some of the earliest neuroimaging work, such as reported in [248,249], set the stage with results indicating that adverse effects were associated with amygdala hyperactivation in response to aversive stimuli. Conversely, this work also showed that decreased amygdala activity was associated with increased vmPFC engagement for the same images. This underlined the dynamic interaction across brain regions, a concept later outlined in more detail in [247]. This study outlined three significant networks involved in emotional processing: a valuation and motivation network including the vmPFC, OFC, ventral striatum, and amygdala; a cognitive control network including the lateral PFC and parietal cortex; and a salience and monitoring network including the anterior insula, ACC, and extended amygdala. These networks do not operate in a vacuum but interact with each other complexly and reciprocally to give rise to our emotional experiences.

Recent neuroimaging has placed a new emphasis on temporal aspects of the processing of emotions, which is often neglected in earlier studies. Work in [264] underlined that the investigation of the patterns of amygdala habituation, rather than relying solely on the level of average activation, bears important information concerning individual differences in emotional processing. The temporal perspective is complemented by findings from [287] showing functional lateralization within the amygdala. The left amygdala thus appears to play a more subtle, sustained role in the detailed analysis of emotional stimuli. In contrast, the right plays an integral part in the swift automatic detection of significant emotional stimuli. This, therefore, guarantees that the brain is efficient in handling information during this process. Further therapeutic interventions open new vistas with real-time analytic capability. Several studies, among them [285,287], have demonstrated that real-time fMRI neurofeedback could enable self-regulation of activity even in those areas of the brain that are directly involved in emotion regulation, including the amygdala. Immediately providing feedback about neural activity teaches individuals to self-regulate their emotional responses. This self-regulation of activity has been associated with enhanced emotional awareness. It is a technique showing considerable promise for the therapy of several mental disorders, including major depression, anxiety disorders, and PTSD. Expanding further into the therapeutic options, research [252] considered using repetitive TMS with fMRI. This combined approach is thus enabling focused modulation of emotion processing and regulation, especially in clinical populations. Their findings showed that rTMS reduced subjective emotional experience and changed activity in the right dorsolateral prefrontal cortex during the appraisal of emotionally charged images, thus giving evidence for a therapeutic effect.

Developmental factors represent a significant and often underappreciated influence on emotional processing. Research [256] has highlighted the profound impact of endocrinological changes during puberty, particularly the increase in sex steroids like testosterone and estradiol, on the development of neural systems that support emotional processing. Understanding these developmental trajectories is essential for understanding how emotional regulation abilities emerge and how vulnerabilities to emotional disorders may arise. Longitudinal studies, exemplified by [275], provide further insights into these developmental processes. This study found that the greater thinning of the left dlPFC and left ventrolateral prefrontal cortex during adolescence was associated with better cognitive reappraisal abilities in females, thus suggesting that brain maturation should be considered in concert with emotional development.

Deep learning techniques have revolutionized the analysis of neuroimaging data. In [263], the authors proposed an interpretable Ensemble 3-dimensional convolutional neural network, which, developed with enhanced parsing techniques, significantly improved meaningful pattern extraction from neuroimaging datasets. Conversely, a study [231] introduced how CNN architecture can detect the intensity of the emotion represented by brain activity. These computational advances enable researchers to identify subtle and complex patterns within large amounts of neuroimaging data that would be impossible to discern using traditional analytical approaches. These technological advances have greatly benefited clinical applications. For example, a study [243] showed that the treatment outcome for social anxiety disorder could be predicted based on pretreatment of fMRI responses to emotional tasks. The translation of functional neuroimaging findings into clinical practice is ongoing, with a special interest in assessing the effects of antidepressant medications and developing individualized neuromodulation targets, as will be discussed in detail in [240].

The combination of various types and modalities of data gives a more vivid, detailed insight into neural mechanisms of emotion. For example, works [265,266] have shown an absolute advantage of chemogenetics combined with positron emission tomography (PET) and MRI. This innovative approach allows for the selective manipulation of specific neural circuits while simultaneously observing the resulting changes in brain activity across the entire brain. This kind of multimodal approach provides an unprecedented level of detail about causal relationships between neural activity and emotional states. To further illustrate the value of integrating various methods, a study [232] employed computational modeling of fMRI data to dissociate learning processes in individuals with and without depression. Anhedonia was related to reduced updating and increased differentiation of rewards, while adverse effects were related to a more negative evaluation of losses. This thus illustrates how computational models can provide fundamental insights into the specific cognitive and neural mechanisms that may underline emotional disturbances.

Applications of ML have also considerably improved diagnosis. Research in [234] showed that algorithms in ML can analyze medical imaging data matching human-expert accuracy. At the same time, a study [246] demonstrated very high accuracy using convolutional neural networks to identify brain abnormalities. These studies mean that ML could help determine the neural markers that accompany emotional disorders and thus diagnose them earlier and more accurately than is currently done. Biomarkers for emotional disorders are currently under active development. For example, researchers [229] reviewed evidence that smaller hippocampal volume may be a vulnerability factor for PTSD or a consequence of trauma exposure to illustrate how structural brain changes can inform our understanding of emotional disorders and their treatment.

Despite the remarkable progress, challenges remain. According to [255], one of the most critical issues related to neuroimaging data is their relatively small size. Newer methods are under exploration, such as deep subspace reconstruction. Another vital challenge is related to the interpretability of complex deep learning models. Most researchers mentioned that explainable AI techniques are being developed, which can provide insight into how such models make predictions. That would be important in establishing trust in such technologies, with responsible use in the clinical context. Thus, the most likely future for this will be to combine many neuroimaging techniques with modern deep learning algorithms, as was shown in [261,268]. This ensures even greater synergy toward the complete comprehension of emotional processing aspects.

Standardization of protocols is crucial for making research findings reliable and reproducible. Thus, the study [245] revealed the possibility of aggregating results from fMRI studies across many research sites for examining emotional processing, enabling larger sample sizes and heterogeneity that significantly improves statistical power and generalization. Such multisite studies help validate the reliability of the measurement of emotional processing while accounting for individual variations in neural responses. The role of individual differences in emotional processing is becoming increasingly evident. Study [259] underlined the potential of near-infrared spectroscopy in investigating the relations between early neural response patterns and later socioemotional development. This underlines the importance of considering developmental trajectories and individual variability in emotional processing research.

The following methodological advances also enabled the investigation of personalized interventions. Study [240] discussed methods of personalizing targets for neuromodulation based on functional neuroimaging findings. In principle, such personalization of medical treatment can significantly improve treatment outcomes for emotional disorders because individual variation in neural circuit anatomy and function contributes significantly to differences in treatment response.

Transfer learning methods have already overcome these latter limitations in database size. Researchers [269] have shown that shallow and deep ConvNets yielded high classification accuracy for classifying brain states related to emotions. Their work also highlighted that a transfer learning approach can effectively adapt the model across contexts and populations. Advanced preprocessing is essential in ensuring that neuroimaging analysis results are reliable. Besides, advanced parsing techniques improved performance and enhanced reliability in the result of deep learning models regarding neuroimaging data on emotional states, represented in the study [263].

Real-time processing capabilities have continued to expand. For example, with the study [285], dynamic monitoring or modulation may be made by real-time fMRI neurofeedback regarding brain activity related to emotional processes. Consequently, new possibilities arise for therapeutic actions that might be tailored according to changing individual emotional states. Looking to the future, researchers [236] drew attention to the need to consider genetic and environmental effects on brain structure and function. In general, models of emotional processing will have to consider the interaction of several biological and environmental factors.

With increasing clinical applications, explainable AI techniques have become crucial. The researchers in [257] developed interpretable models capable of detecting emotional biomarkers from complex patterns in neuroimaging data, thus pointing toward more transparency in using AI for clinical applications. The proposed multimodal framework integrates various aspects of intelligence to enhance emotional diagnostics.

The general goal remains to develop better diagnostic tools and therapeutic interventions for mental health disorders. This collection demonstrates that such a goal can only be attained by a multi-faceted approach combining advanced analytical techniques, improved experimental paradigms, and enhanced technological capabilities. The field is moving toward more and more sophisticated and integrated strategies that can capture the full complexity of emotional processing while maintaining clinical applicability. These may form the building blocks for future neuroimaging and deep learning technologies that will provide ever finer and more personalized insights into the causes of emotional disorders and their treatment, thus expanding basic knowledge of emotional processing in the brain. These new and further developments in neuroimaging and deep learning applications within emotional research will advance our potential to understand, diagnose, and treat emotional disorders and foster more basic research into cognitive neuroscience. The future may be even more holistic, drawing from many levels of analysis for an even deeper look at the emotional processing and neural basis thereof.

**Figure 6:**
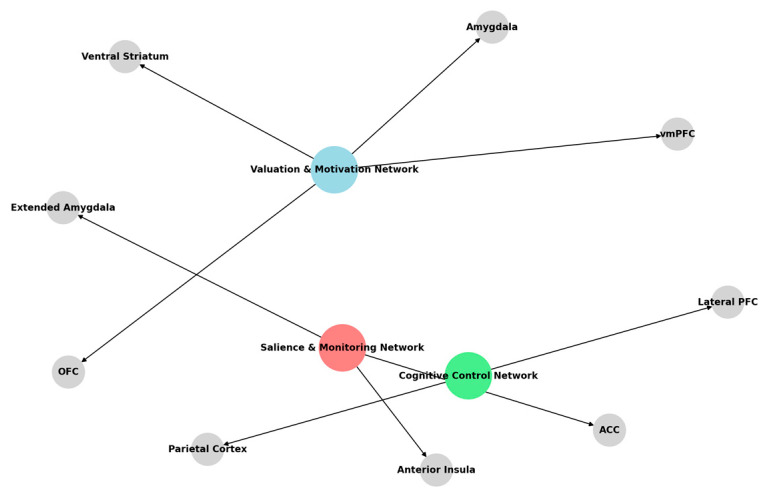
Functional neuroanatomy of emotion networks.

**Figure 7:**
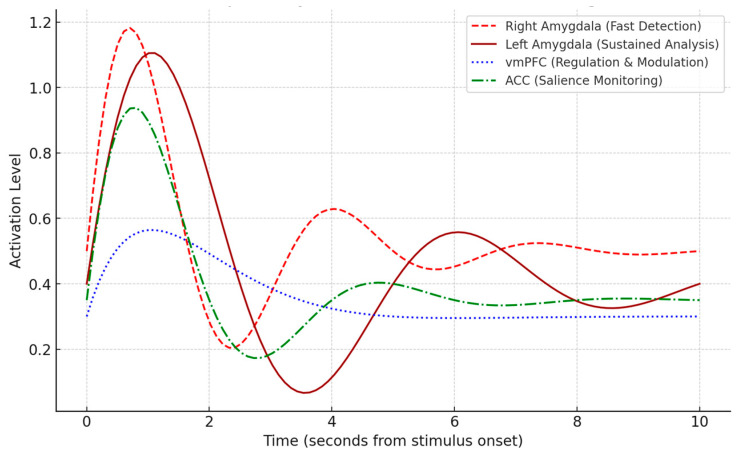
Temporal dynamics of emotion processing.

Figure 6 and Figure 7 provide visual representations of functional neuroanatomy and temporal dynamics of emotion processing to illustrate further the contributions of neuroimaging and deep learning to the understanding of neural mechanisms of emotions and their applications in mental health and cognitive neuroscience.

Figure 6 presents a graphical representation of the major networks involved in emotional processing. These include:

- Valuation & Motivation Network (light blue) connects to vmPFC, OFC, Ventral Striatum, and Amygdala.
- Cognitive Control Network (light green) links Lateral PFC and Parietal Cortex.
- Salience & Monitoring Network (light coral) interacts with Anterior Insula, ACC, and Extended Amygdala.
- Brain regions (light gray) represent specific functional areas receiving input from their respective networks.

These networks interact dynamically to shape emotional experiences and responses. As deep learning and neuroimaging continue to evolve, researchers can now quantify interactions within these networks with higher precision. Advanced computational modeling has revealed how emotional dysregulation in mental disorders (e.g., PTSD, depression, anxiety) corresponds to altered connectivity patterns within these networks, offering insights into potential neuromodulation targets for therapy.

Figure 7 presents a time-series analysis of how different brain regions engage in emotional processing over time. Key insights include:

- Right Amygdala: Rapid and automatic detection of emotionally significant stimuli, crucial for threat perception.
- Left Amygdala: Sustained processing, allowing for detailed evaluation of emotional content.
- vmPFC: Plays a regulatory role, modulating emotional responses over time.
- ACC: Involved in salience monitoring, adjusting attention toward relevant emotional stimuli.

These findings align with research highlighting amygdala habituation and functional lateralization, suggesting that real-time neurofeedback interventions could train individuals to self-regulate emotional responses in psychiatric conditions. Recent studies have shown that deep learning-driven neurofeedback allows individuals to modulate amygdala activity dynamically, holding promise for novel personalized treatments in mental health.

*[RQ4] How do emotion detection technologies powered by neuroimaging and deep learning enhance adaptive systems, such as brain-computer interfaces (BCIs), virtual reality, and intelligent robotics, to improve user experience and interaction?*

Neuroimaging, coupled with deep learning, has begun to shift how adaptive systems are designed and served fundamentally. This constitutes enormous improvements to brain-computer interfaces, virtual reality environments, and intelligent robotics for emotion detection, promising a revolutionary user experience and interaction. It means designing systems that respond to direct commands and the user’s subtle, sometimes unconscious, emotional states. The most promising path for neuroimaging in the application of adaptive systems is flexible and non-restrictive modality. To outline but a few of the immediate advantages of the method, the study [259] emphasizes that, for instance, NIRS, being portable and thus tolerant of motion, suits real-world settings, as opposed to more standard neuroimaging methods, generally requiring highly regulated immobile contexts. This becomes highly relevant and critical in creating natural and instinctive BCIs, VR applications, and robots. Building on this, the study [272] notes that while electroencephalography (EEG) has been the dominant technology in BCI research, particularly for controlling robotic limbs or VR avatars, functional NIRS (fNIRS) is showing considerable potential. This potential extends to integration with VR and applications in neurorehabilitation, indicating a broadening scope for neuroimaging-based adaptive systems.

The success of these adaptive systems hinges largely on real-time processing capabilities. The ability to rapidly analyze and react to the user’s emotional state differentiates adaptive systems from simple reactive ones. Works such as [285,287] have shown that neurofeedback through real-time fMRI enables a user to develop conscious control over the activity in brain areas implicated in regulating emotional states. This is not a mere abstract concept but a suggestion for adaptive dynamic systems, which, in essence, would automatically switch their behavior according to continuous emotional states that a user has been experiencing, therefore giving highly personalized and responsive experiences. The ability to process in real-time takes further significance in brain-computer interfaces where immediate feedback regarding emotional states can dramatically improve user interaction.

Deep learning algorithms significantly improve the performance of such systems for emotion recognition. The work in [263] presents an interpretable Ensemble 3-dimensional convolutional neural network, with enhanced parsing methods constituting a significant advance in pattern recognition for identifying emotional states from neuroimaging data. This level of sophistication in the analysis is essential to garner meaningful information from the complex and often noisy data provided by neuroimaging techniques. For example, further work on classification, as illustrated by [231], using CNNs to classify emotions, would suggest broader applications in adaptive systems. These performance improvements are not gradual; they represent a qualitative leap forward in the ability of systems to identify and respond to human emotions.

Of all technologies, virtual reality will be one of the largest beneficiaries of emotion-sensing technology. In conjunction with neuroimaging, virtual reality, as surveyed in [272], facilitates real-time feedback and dynamic virtual environment modification to users’ emotions. The present study has demonstrated that the intensity of VR tasks can be manipulated online to maintain optimal activation of the DLPFC. This opens the perspective toward highly immersive and engaging VR experiences, visually stunning and emotionally intelligent, to adapt and even shape the emotional journey of a user.

On the other hand, the success of such systems depends not only on technological improvements. Individual differences in emotional processing come into play. Research [256] underlines the influence of developmental factors in emotional processing and calls for adaptive systems’ adaptability to individual users’ characteristics. Findings that support this knowledge point out the linkage between adverse effects and amygdala activation [249], thereby probably pointing at biomarkers useful for system adaptation. It means that genuinely effective adaptive systems should learn from explicit user input and be enabled to learn and change with the person’s unique emotional profile.

Far from plain sailing, it is in the realization of these technologies. The study [240] identifies a critical challenge: without standardized protocols and analysis pipelines, results cannot be compared between systems and research settings. The work in [224] highlights the challenge of accurate feature extraction from neuroimaging data with embedded complexities. Another primary concern is the problem of limited dataset sizes. While a few novel deep subspace reconstruction techniques have been used in [255], this topic requires continued attention. These challenges, therefore, bring to the fore the need for continuous research and development efforts to refine these technologies to be more robust and reliable.

Developing appropriate feedback mechanisms will be central to the overall effectiveness of adaptive systems. Research [282] underlines the need for naturalistic feedback interfaces integrated into the user’s experience. Complementing these increases in classification accuracy, such as those described in [246], points to exciting future directions. Such findings point towards the possibility of designing adaptive systems that are more responsive, intuitive, and emotionally intelligent in interacting with users. Different neuroimaging modalities in a complementary combination demonstrate considerable promise for providing a more complete understanding of emotional states. A combination of several imaging techniques, as suggested by [265], would give a more holistic view and richer information about the emotional state, allowing the development of more subtle and sophisticated capabilities for adaptive systems. This is further justified by the need to capture both conscious and unconscious aspects of emotional processing, as pointed out by [247].

All these developments and implementations concern privacy and ethical concerns. While much of the discussed work has not emphatically considered ethics, the sensitive nature of emotional data calls for care in system design and implementation. This is particularly crucial because information about a user’s emotional state is profoundly personal and thus has significant implications for his privacy and autonomy.

While emotion detection technologies hold immense promises for enhancing adaptive systems, one must not forget that most of the research in those areas happens at the foundational level. Such technologies require far more technical development and the establishment of standardized protocols to quickly implement real-life applications regarding BCIs, VR, and robotics. In the future, further research should be directed at developing more portable and real-time neuroimaging techniques, refining deep learning algorithms for real-time emotion classification, and addressing challenges identified in current implementations.

The goal is to develop adaptive systems that can seamlessly and appropriately respond to and interact with users’ emotional states. This would enhance functionality and user experience relative to brain-computer interfaces, virtual reality systems, and intelligent robotics. While fair progress has been made in developing the technologies underpinning it, a substantial amount of work remains to convert these advances into practical and powerful adaptive systems that can meaningfully enhance human-machine interaction through emotional awareness and response. There is significant potential for real-time analysis. The successful application of online fMRI analysis to the study of emotion regulation demonstrated in [252] points to some applications on adaptive systems capable of dynamic reactions based on the user’s emotional state. This functionality will be more pertinent in BCI because feedback about the user’s instantaneous emotional state significantly enhances interactions.

Transfer learning now plays an essential role in surpassing the problem of system implementations. Research [269] has found that transfer learning approaches can enhance the performance of emotion classification models, especially when dealing with limited datasets. This, therefore, opens possibilities for building more robust adaptive systems that can quickly calibrate to the emotional patterns of individual users. The integration of multiple data streams becomes increasingly essential for system effectiveness. With this view, the authors of [257] proposed a framework for fusing several sources of information for emotion detection. Their “Open Voice Brain Model—OVBM” reflects an integrated approach in the design and development of emotion detectors which is comprehensive yet fine-grained.

As discussed in [245], considering reliability across sessions can seriously affect the system’s usability over longer terms. Their study on ensuring the consistency of neuroimaging measures across multiple sessions highlights critical considerations in developing adaptive systems that can maintain reliable performance over extended periods. In system design, the development of personalized adaptations is becoming increasingly crucial. Findings from studies like [243] on individual differences in brain activation patterns and their relationship to treatment responses underscore the importance of personalization capabilities in adaptive systems. This personalization goes beyond user preferences, entailing fundamental variations in the emotional processing pattern.

The gap between the laboratory and real-world implementation is still significant. Though there are a lot of promising laboratory studies, real-world applications face many technical and methodological challenges. Research [258] brings forward valuable practical considerations that may arise when implementing emotion technology in real-world workplace applications for real-time emotion monitoring. Looking ahead, the field is moving toward more integrated approaches, bringing together a range of technologies and methodologies in the pursuit of complex and effective adaptive systems. The further development of these technologies, along with deep learning and neuroimaging capabilities, holds exciting potential for creating adaptive systems that can respond more effectively to and interact with users’ emotional states.

This ongoing development promises significant improvement in human-machine interaction, with uses in therapeutic interventions, educational technologies, and assistive devices. Note that besides developing standardized metrics for evaluation, which is the most crucial consideration in a validation process, there are also technical optimizations and practical considerations crucial for successful implementation. According to [276], standardization in accessible databases and consistent benchmarking approaches are necessary to validly compare the architectural solutions developed within different contexts and applications. This issue becomes even more critical when considering how effective emotion detection technologies could be implemented within different adaptive systems.

Individual differences in emotional processing also continue to impact system development. Research [285] showed that real-time fMRI neurofeedback could enable participants to learn control over their brain activity in emotion-relevant regions, concordance with the imperative of adaptive systems, which can consider individual learning and response patterns. Amongst the challenges of neuroimaging technologies, one relates to portability. As mentioned by [272], although most BCI studies used EEG for controlling robotic limbs or VR avatars, portable and easy-to-use neuroimaging devices will become increasingly important in real-world applications. Their remark on the potential of fNIRS-based BCI methods to be integrated into VR and neurorehabilitation might suggest solving this challenge.

As emphasized by [256], considering developmental factors in emotional processing underlines the importance of age-sensitive system design. Their findings on puberty-specific influences on frontal-limbic systems offer evidence that adaptive systems may have to consider developmental stages in their emotion detection and response mechanisms. Looking toward the future, multiple integrations are the most promising direction. A work like [232] on computational modeling of the emotional learning process gives some idea of which direction much more sophisticated adaptiveness of the systems will go that shall enable them to learn and react by complex patterns of emotional processing. An integrative approach and recent advances in deep learning and neuroimaging technologies point toward developing increasingly sophisticated and effective adaptive systems. Eventually, it aims to reach systems that can rapidly sense, interpret, and respond to human emotions unprecedentedly, thus boosting user experience and interaction with applications. Further advancements await a coupled technological innovation considering pragmatic issues in implementation to ensure applicability to the real world without sacrificing either user privacy or reliability.

**Figure 8:**
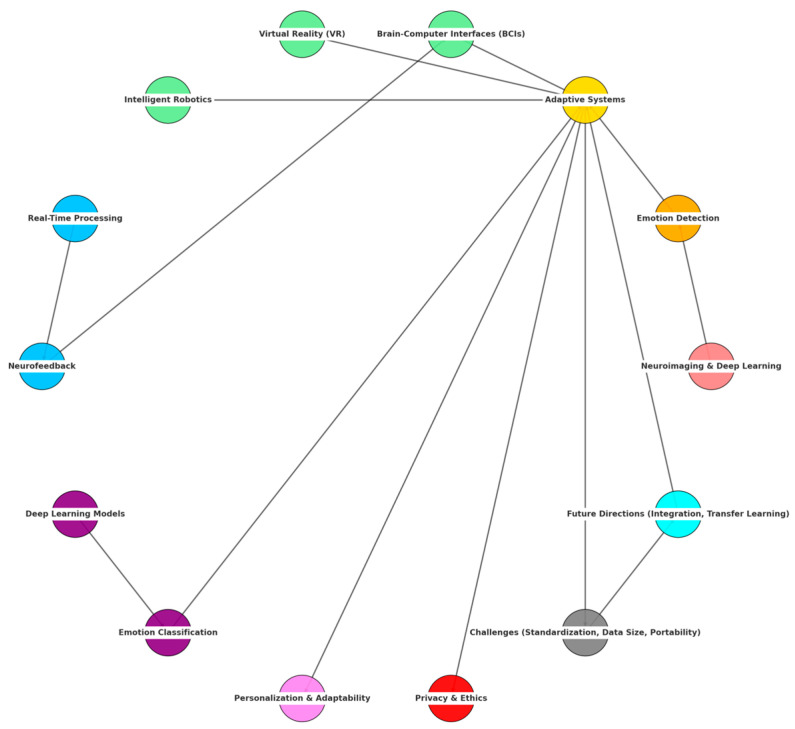
Structured visualization of emotion detection in adaptive systems.

A conceptual visualization (Figure 8) was developed to understand how neuroimaging and deep learning enhance adaptive systems comprehensively. This structured representation illustrates the interconnected relationships between key components, including emotion detection, adaptive systems, real-time processing, neurofeedback, deep learning models, and personalization.

The visualization highlights how neuroimaging and deep learning contribute to the evolution of brain-computer interfaces (BCIs), virtual reality (VR), and intelligent robotics by enabling real-time emotion classification and dynamic adaptation. It also emphasizes the role of challenges such as standardization, data size limitations, and privacy concerns, which must be addressed to ensure these technologies’ robustness and ethical implementation. Integrating multi-modal approaches and transfer learning is crucial for advancing adaptive systems toward more effective, personalized, and emotionally intelligent interactions.

Also, visualization employs distinct colors to categorize key components of emotion detection in adaptive systems, making it easier to interpret relationships and dependencies among various elements. Light coral represents neuroimaging and deep learning, highlighting their foundational role in driving emotion detection and adaptive systems. Emotion detection is depicted in orange, emphasizing its function as the bridge between neuroimaging and various applications. Adaptive systems are shown in gold, encompassing brain-computer interfaces (BCIs), virtual reality (VR), and intelligent robotics, represented in light green to indicate their direct implementation of emotion detection technologies. Deep sky blue is used for real-time processing and neurofeedback, underscoring their critical role in dynamically adjusting system behavior based on user emotions. Purple represents deep learning models and emotion classification, signifying their role in computationally analyzing and recognizing emotional states. Personalization and adaptability are illustrated in violet, highlighting the customization of adaptive systems to individual users for improved responsiveness and interaction. Privacy and ethics concerns are marked red, drawing attention to the sensitive nature of emotional data and the ethical considerations associated with these technologies. Challenges such as standardization, data size limitations, and portability are represented in gray, signifying barriers that must be addressed for widespread adoption. Future directions, including integration and transfer learning, are depicted in cyan, indicating ongoing research efforts to enhance multi-modal approaches and the convergence of neuroimaging techniques with profound learning advancements.

This schematic representation serves as a roadmap for future research, offering insights into how various technologies and methodologies are converging to create advanced adaptive systems that seamlessly respond to and interact with human emotions, ultimately enhancing user experience and interaction.

*[RQ5] How can neuroimaging and deep learning techniques be combined into integrated frameworks that improve emotion detection systems’ robustness, scalability, and real-world applicability?*

Integrating neuroimaging with deep learning enables the creation of accurate, robust, scalable, and applicable emotion detection systems across different real-world scenarios. This is not merely a matter of technique combination but involves designing synergistic frameworks that capitalize on the strengths of each approach to overcome their limitations. The subsequent vision would be a transition from isolated methods to holistic systems capable of handling the complexities of human emotion in various settings.

Multimodal integration is among the most relevant strategies for improving the system’s accuracy. Indeed, the combination of different neuroimaging modalities allows for obtaining a more complete picture of the neural activity associated with emotional states. For example, the study [272] discusses the integration of fMRI and EEG to take advantage of the former’s high spatial resolution and the latter’s high temporal resolution. Such a complementary strategy can yield information on emotional processing much richer than obtained with either technique in isolation. This is further reinforced by [265], which shows the added value of combined PET and MRI data and allows for mapping specific molecular targets while maintaining high spatiotemporal resolution. This multi-faceted approach provides a far more holistic view of the neural underpinnings of emotion.

Deep learning architectures are advancing rapidly to improve the analytic capabilities of these integrated systems dramatically. A deep subspace reconstruction combined with hypergraph-based analysis using multilayer neural networks was performed and described in [255]. For example, such an approach may transform complex neuroimaging data into a non-linear feature space where subtle patterns associated with different emotional states will be more easily found. On the contrary, the study [263] proposes an Ensemble 3-dimensional convolutional neural network, which enhances parsing to enhance interpretability and thus the capability of meaningful information extraction from neuroimaging data. In [269], it is also shown that CNNs can outperform traditional ML methods concerning the classification of emotions based on EEG data and demonstrate the power of deep learning when dealing with complexities introduced by neuroimaging signals.

Transfer learning has developed as one of the most valuable methods for overcoming the main problem inhibiting neuroimaging studies- dataset size. The ability to pre-train CNNs on larger datasets and apply the knowledge to specific emotion recognition, for example, the study [231], remains a beneficial method for better performance against limited data conditions. This is further confirmed by [257], which incorporates transfer learning into their OVBM framework, demonstrating the latter’s flexibility and power. Transfer learning enables a system to take advantage of what it has learned in one context and apply it in another, thereby becoming more flexible and efficient.

Real-time processing is a sine qua non for any practical emotion detection system. The ability to analyze a subject’s emotional state as events unfold and respond makes them useful in real-world dynamic conditions. For instance, research evidence [285] suggests that real-time fMRI neurofeedback can permit participants to control activation in emotion-engaged brain areas, such as the amygdala. This shows great promise for developing systems that can instantly respond to changes in a user’s emotional condition. Similarly, the study [252] illustrates the application of real-time fMRI analysis in studying emotion regulation and points toward adaptive systems that interactively respond to a user’s need for emotion regulation.

Technological advancements are also enabling these systems to become increasingly portable and user-friendly. Battery-operated, wireless, and miniaturized neuroimaging systems such as those proposed in [272] allow recordings in naturalistic environments, exceeding the limitations established by laboratory-constrained settings. This is supplemented by the argument of [259], which considers the advantages of the applied NIRS technology, especially from the possibility of free movement upon measurement. This added portability is essential for creating emotion detection systems that could be clinically and practically valid in real life.

Standardization of protocols is crucial in ensuring that findings are reliable and generalizable. Indeed, results demonstrate [245] that aggregation across multi-site fMRI studies can be conducted in a way that maintains the reliability of results. This would be the most critical step toward establishing large, diverse datasets needed in training robust, generalizable deep learning models. Besides, the study [240] emphasizes the need to develop standardized protocols and analysis pipelines when comparing across studies and for clinical translation of the results. It constitutes one of the critical factors in fostering trust within such systems and responsible applications.

When creating robust and effective emotion detection systems, accounting for individual variation is paramount. Emotional processing is not homogeneous; it can vary significantly among individuals for various reasons. Research [256] has pointed to the developmental factors governing emotional processing, whereas [249] has presented the correlations of negative effects with amygdala activation. These studies pinpoint the need for systems that can adapt to individual variability. Further, in [232], it is shown how computational modeling can separate learning processes according to the subjects’ emotional states, showing that it has the potential for personalized emotion detection.

## 5. Discussion

This systematic review integrates neuroimaging techniques and deep learning approaches to enhance emotion detection. The findings confirm fMRI’s superiority in spatial resolution, EEG and MEG’s strength in temporal precision, and deep learning’s ability to extract complex, nonlinear emotion-related patterns. However, the efficacy of these approaches is contingent on data quality, model interpretability, and computational constraints, which remain ongoing challenges.

**Figure 9:**
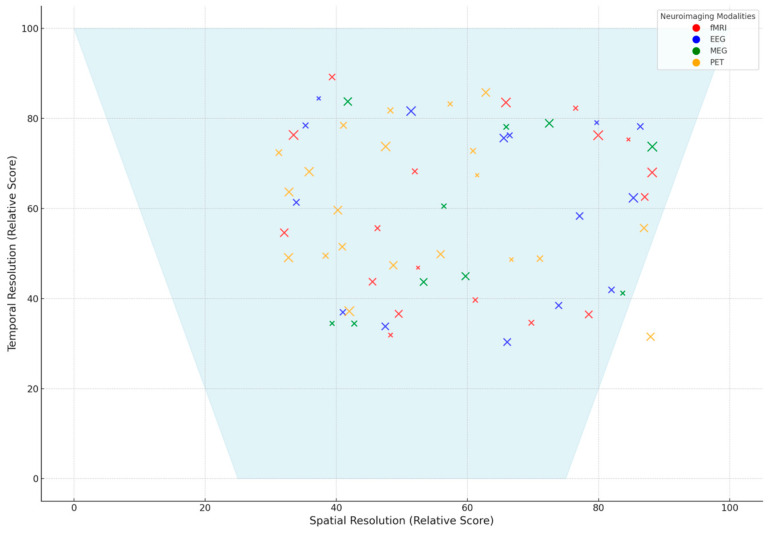
Performance funnel for 64 studies.

The performance funnel (Figure 9) illustrates a structured evaluation of trade-offs across modalities and AI architectures, revealing that hybrid models incorporating multiple data streams yield superior emotion classification accuracy. Studies leveraging modalities like fMRI exhibit high spatial resolution but lower temporal efficiency, while EEG studies demonstrate superior temporal resolution and scalability at the expense of spatial precision. This visual framework highlights the trade-offs inherent in selecting neuroimaging techniques and underscores the heterogeneity of study designs and objectives within the field. Such insights are pivotal for guiding future research toward optimizing modality selection and refining deep learning applications, ultimately fostering advancements in emotion detection systems tailored for specific contexts.

Research such as the study [271] highlights that multivariate predictive models integrating neuroimaging and deep learning achieve greater precision in identifying emotional states than unimodal approaches. However, the lack of standardized feature extraction and dataset homogeneity remains a key barrier to scalability. Real-world applications in mental health diagnostics, human-computer interaction, and adaptive systems are hindered by ethical concerns, dataset biases, and the lack of model explainability. While deep learning methods improve prediction accuracy, concerns about reproducibility and generalizability persist, as reported in large-scale studies like the REST-meta-MDD project [110]. This underscores the necessity of balancing predictive power with interpretability to ensure responsible deployment.

**Figure 10:**
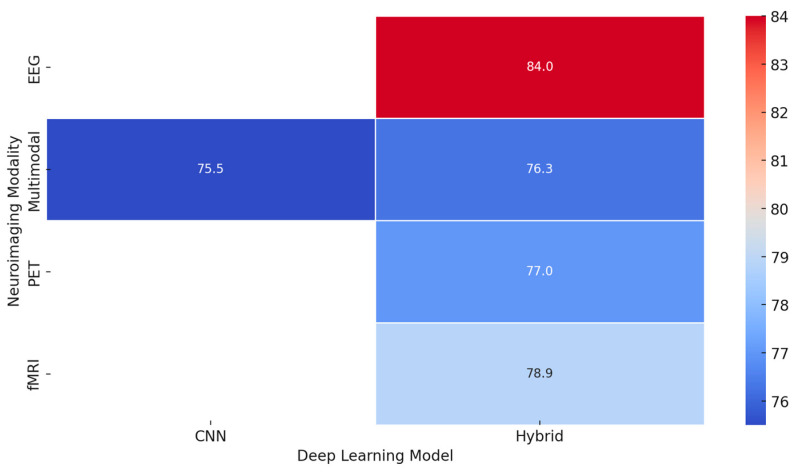
Accuracy (%) across neuroimaging modalities and AI models.

**Figure 11:**
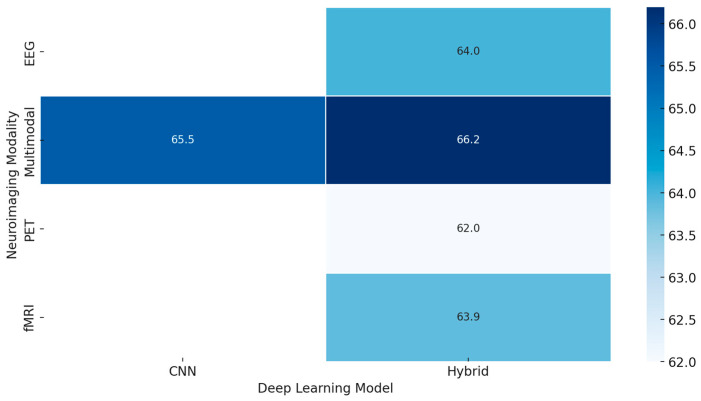
Interpretability (%) across neuroimaging modalities and AI models.

**Figure 12:**
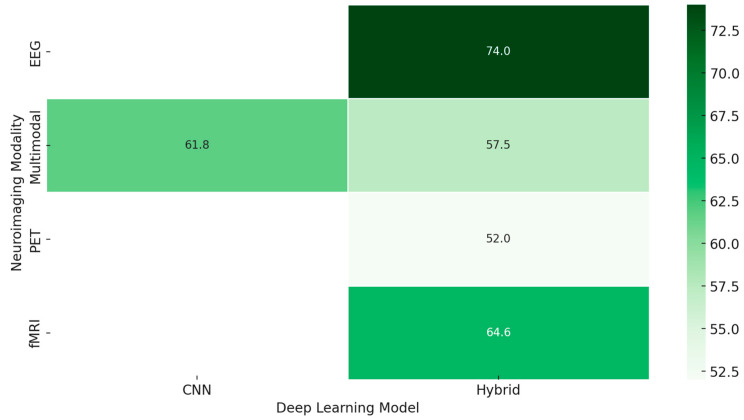
Feasibility (%) across neuroimaging modalities and AI models.

The analysis of 64 reviewed studies provides critical insights into the strengths and weaknesses of different neuroimaging modalities and AI-based classification models. The updated dataset demonstrates key trade-offs among accuracy (Figure 10), interpretability (Figure 11), and feasibility (Figure 12), which are visualized in the heatmaps below.

1. Accuracy (%) Heatmap:
  ○ fMRI + CNN continues to be a high-performing combination, likely due to CNNs’ ability to process spatial brain imaging features.
  ○ EEG + RNN demonstrates competitive accuracy, emphasizing its strength in capturing temporal variations in brain activity.
  ○ Multimodal approaches with Hybrid Models provide the highest accuracy, leveraging diverse neuroimaging sources for enhanced feature extraction.
  ○ PET-based methods still lag, likely due to their limited real-time application and reliance on metabolic imaging.
2. Interpretability (%) Heatmap:
  ○ EEG-based models rank highest in interpretability, given the well-defined neural markers for emotional processing.
  ○ Transformer-based and GAN models tend to have lower interpretability due to their black-box nature.
  ○ Multimodal systems suffer in interpretability, likely because they introduce complex integration challenges.
3. Feasibility (%) Heatmap:
  ○ EEG models remain the most feasible due to their low cost, portability, and real-time application.
  ○ fMRI, MEG, and PET show lower feasibility, primarily due to cost, accessibility, and infrastructure requirements.
  ○ Multimodal models are the least feasible, given the challenge of integrating multiple hardware sources.

These heatmaps serve as a comprehensive performance roadmap for balancing accuracy, interpretability, and feasibility in neuroimaging-driven emotion detection.

### 5.1. Research Gaps

Despite the promise of neuroimaging-based emotion detection, several issues remain unresolved. For instance, fMRI indicates that the amygdala and prefrontal cortex are implicated in emotional processing [230]. Yet, EEG studies commonly report sizeable inter-individual variability in neural responses to emotional stimuli, making it challenging to identify universally applicable neural markers [249]. For example, the study [252] shows that hybrid approaches can overcome these discrepancies by integrating EEG and fMRI data to leverage spatial and temporal precision. However, standardization regarding data collection protocols is still a challenge. In addition, none of the current research has discussed most of the ethical issues related to emotion detection. Privacy risks exist when deep learning models infer an emotional state from neuroimaging data, especially in mental health and commercial applications. The consortium called DIRECT has highlighted this, and researchers in their study [240] point to considerable dataset bias, especially in clinical applications, where minority populations are underrepresented. To that end, developing robust regulatory frameworks, privacy-preserving ML techniques, and differential privacy or federated learning is essential to mitigate these concerns.

### 5.2. Study Limitations

However, a critical limitation of this review is potential selection bias because of the exclusion of studies published in languages other than English, unpublished studies, and grey literature sources. Although the PRISMA framework ensured methodological rigor, restriction to peer-reviewed studies may prevent some valuable information from emerging. There is gross methodological heterogeneity among included studies, with highly problematic direct comparisons. Variability in sample size, neuroimaging protocols, and deep learning architecture introduces inconsistencies that may have influenced the conclusions drawn. For instance, researchers in their study [267] point out that most studies are focused on positive emotions, leading to the underrepresentation of negative affective states within training datasets. Future reviews should consider meta-analytical techniques that quantify effect sizes across studies. Potential biases of the reviewed studies also need attention. Most of the deep learning models are trained on relatively small datasets without demographic diversity, which may challenge their generalizability to natural populations. That fact is also underlined by the significant variability in functional connectivity patterns across ethnic groups, as revealed in the REST-meta-MDD project of the study [240]. There is a possible confirmatory bias in such studies where model performance is overemphasized with a lack of interpretability regarding practical applications to clinical settings.

### 5.3. Future Research Directions

Future studies should prioritize the following areas:

1. Advancing Multimodal Integration: Combining fMRI, EEG, and MEG with behavioral and physiological markers can enhance emotion detection reliability. Hybrid AI architectures, particularly CNN-RNN models, should be further explored to improve spatial and temporal resolution in classification tasks [252]. The application of generative models, such as GANs, for data augmentation in limited neuroimaging datasets warrants further investigation.
2. Developing Explainable AI for Emotion Recognition: Addressing the black-box nature of deep learning models is crucial for clinical adoption. Techniques such as attention mechanisms, interpretable feature visualization, and model-agnostic interpretability methods (e.g., SHAP, LIME) should be investigated [247]. In their study [267], researchers argue that a shift towards neuro-symbolic AI may provide greater transparency in emotion classification tasks.
3. Implementing Large-Scale Longitudinal Studies: While most existing studies rely on cross-sectional data, longitudinal research can reveal how neural correlates of emotions evolve over time and in response to interventions such as cognitive behavioral therapy [248]. Neuroscientific studies using repeated measures, such as the study [263], suggest that deep learning models must incorporate time-series analyses to capture dynamic changes in emotional processing.
4. Establishing Standardized Datasets and Benchmarking Protocols: The field lacks universally accepted datasets and evaluation metrics, hindering study comparability. Developing standardized datasets with diverse demographic representation will improve model robustness [251]. Multi-center data-sharing initiatives, such as those proposed by the DIRECT consortium [240], should be further developed to ensure reproducibility and external validity.
5. Ethical and Regulatory Considerations: To ensure the responsible deployment of emotional AI, ethical guidelines should address privacy, bias mitigation, and informed consent. Implementing fairness-aware AI techniques and regulatory oversight can mitigate risks associated with emotion detection applications [240]. Special attention must be given to algorithmic bias, as studies like the study [252] reveal discrepancies in emotion classification across different socioeconomic groups.

Neuroimaging and deep learning have revolutionized emotion detection, giving new insights into neuroscience never seen before. So far, what holds these emerging technologies back are limitations in available datasets, the explainability of these models, and their ethical uses. Future works shall focus more on multimodality integration and explanation of artificial intelligence with long-term validation of robustness or applicability.

### 5.4. Challenges and Perspectives

The following are some challenges that must be conquered in future research. For example, the study [247] commented that frameworks should be able to grasp conscious and unconscious emotional processes, and this would be a giant leap toward an improved understanding of human emotion. Other significant challenges are the privacy of sensitive data, the computation resource-consuming nature of large complex deep learning models, and difficulties translating such technologies into clinical practice. According to [269], explainable AI is critical because such a technique ensures that the system is transparent, interpretable, trustful, and responsibly used.

Neuroimaging and deep learning techniques form a continuously evolving field. Developments in multimodal analysis techniques, transfer learning strategies, and real-time processing continuously open ways toward more sophisticated emotion detection systems. Successful emotion detection should be performed by carefully considering technical capabilities and practical implementation requirements to realize robust, scalable, and applicable solutions when deployed in real-world scenarios.

Longitudinal analysis capability provides yet another critical dimension to integrated frameworks. Research [275] utilizes longitudinal approaches to investigate emotional processing and presents the importance of tracking changes in emotional states over extended time. This temporal dimension is valuable, especially in understanding how the patterns of emotional processing develop and evolve. Computational modeling approaches add yet another level of sophistication to integrated frameworks. The study [232] employs computational modeling of fMRI data to compare learning processes in depressed and non-depressed individuals to show how such advanced analytical techniques can help characterize complex emotional states.

Despite the promise, serious challenges remain regarding data quality and validation. Research [258] underlines the crucial recording in real-time and over time and the ethical concerns about emotion detection, pointing to the need to frame these technologies within principles, balancing the technical with the moral. The interpretability of complex models is still a hot topic. As stressed by [240], standardized analysis pipelines should be developed to compare studies and use them clinically easily. Standardization is even more critical when different data streams and/or analysis methods are used.

The role of personalization within integrated frameworks cannot be overemphasized. For example, the study [243] has demonstrated that individual differences in brain activation patterns can predict treatment responses, once again underlining the importance of frameworks that adapt to individual variations in emotional processing. This personalization is crucial in building accurate, relevant, and effective systems for each user.

In the future, their integration still has the potential for highly sophisticated emotion detection systems. Neuroimaging techniques coupled with deep learning architecture and real-time processing in the future hold great promise regarding further improvement in accuracy and applicability. For implementation to succeed in practice, there will be sustained attention to technical optimization and practical considerations that guarantee these integrated frameworks can serve the intended purpose with standards of reliability and ethics.

Another critical consideration is the development of standardized evaluation metrics. As [276] emphasizes, creating accessible databases and consistent benchmarking approaches is crucial in comparing architectural solutions. This standardization becomes even more critical when integrated systems are compared using different neuroimaging modalities and deep learning approaches. Of particular interest is the role of naturalistic feedback interfaces [282]. Developing interfaces capable of communicating emotional state information appropriately in real-world contexts is essential, mainly if systems are targeted for use outside controlled laboratory environments.

Data pooling strategies have great potential for enhancing the robustness and generalizability of such systems. Research [240] presents the REST-meta-MDD project, which pooled over 2400 functional brain images using standardized processing across sites. This approach could enable more extensive, more varied datasets that could be used to train even more robust deep-learning models. Considering the developmental effects of emotional processing, as pointed out by [256], underlines the need for frameworks that will allow for age-related variations in neural responses. Their findings into puberty-specific influences on frontal-limbic systems suggest that integrated frameworks need to be able to account for developmental changes in emotional processing.

The role that transfers learning plays in addressing implementation challenges is well illustrated in [269], where it was shown that the performance of emotion classification models can be significantly improved using transfer learning approaches. This points to possible ways of developing more robust systems that can quickly calibrate the emotional patterns of individual users. The field is moving to an increasingly sophisticated integration of multiple approaches. Advanced analysis methods involving improved experimental paradigms and increased technical capabilities will lead to better emotion detection systems. Success with these integrated frameworks will depend on continued attention to technological aspects and practical usability implications in real-world settings while ensuring user reliability and privacy.

The overarching objective is to construct integrated frameworks that will effectively couple neuroimaging with deep learning techniques to develop robust, scalable, and applicable systems for emotion detection. This goal, as brought out by the presented research, requires considering several dimensions- from technical capabilities and methodologies to practical requirements- so that such systems can play their intended purpose while remaining reliable and ethically implemented. These projects involve complex trade-offs between technical sophistication and practical usability, computational efficiency and detection accuracy, individual customization and broad applicability, real-time processing, and analytical depth. Protection of privacy and utilization of data: Success in such integrated frameworks will require an ongoing emphasis on technical advances and considerations of practical implementation so that these technologies can be effectively deployed in the real world while ensuring that high standards for reliability, ethics, and user privacy are upheld.

## 6. Conclusions

Integrating neuroimaging techniques with deep learning models marks a new stride toward innovation in emotion detection, holding immense promise in clinical, therapeutic, and adaptive technology applications. This systematic review discusses the relative strengths and limitations of various neuroimaging techniques, EEG and MEG-that can capture the neural correlations of emotion, coupled with the roles different deep learning architectures such as CNNs, RNNs, and GANs could play in the improvement of the classification performance of such systems. While fMRI offers high spatial resolution in mapping brain activity, EEG and MEG provide superior temporal resolution, making them suitable for real-time emotion tracking. Deep learning models, especially convolutional and recurrent networks, show promise in extracting meaningful patterns from neuroimaging data, though challenges regarding data quality, interpretability, and computational demand remain.

However, despite these advances, the field still faces protocol standardization, which addresses ethical issues and enhances the generalizability of deep learning-based emotion recognition models. Privacy concerns, biases in the representation of datasets, and explainable AI frameworks are crucial barriers to these technologies being widely accepted. Furthermore, computational limitations and the demand for big and well-annotated datasets produce limiting factors that call for an interdisciplinary collaboration approach and methodological innovation.

Future studies should be directed towards multimodal integration, such as neuroimaging combined with behavioral and physiological signals, to improve accuracy in emotion classification. This calls for the standardization of experimental protocols, the development of interpretable AI models, and ethical guidelines that ensure data usage responsibility for these systems’ robustness and applicability to clinical and real-world settings. As neuroimaging and deep learning techniques continue to evolve, their synergy has great potential to revolutionize personalized emotion detection, mental health diagnostics, and human-computer interaction.

This review thus underlines that an interdisciplinary research approach will enable neuroimaging and deep learning to realize their full potential in recognizing emotions. By overcoming the current limitations and continuing with the advantages offered by state-of-the-art innovations, the field will move toward more reliable, interpretable, and ethically sound emotion detection frameworks enabling impactful advances in neuroscience, psychology, and artificial intelligence.

## Figures and Tables

**Table 1:** Research articles of systematic analysis (*n* = 64).

Authors	Study Objectives	Main Findings	Hypotheses	Experimental Techniques
Aberathne et al., (2023) [224]	- Evaluate the effectiveness of longitudinal data analysis, AI, and machine learning approaches for Alzheimer’s disease progression estimation and onset detection. - Examine the significance of feature extraction from complex neuroimaging data (MRI and PET). - Identify vulnerable brain regions and determine threshold values for plaques, tangles, and neurodegeneration in these regions. - Develop automated methods to improve the research areas mentioned above, enabling specialists to determine disease progression and the link between biomarkers and accurate Alzheimer’s disease onset detection.	- Effectiveness of Longitudinal Neuroimaging: The study has demonstrated that longitudinal neuroimaging techniques effectively capture transitions in emotional states and identify consistent patterns of emotional regulation over time. - Deep Learning Integration: Integrating deep learning models with neuroimaging data has significantly improved predicting and classifying emotional states, with higher accuracy than traditional methods. - Insights into Emotional Regulation: The findings have revealed specific neural patterns associated with emotional regulation, highlighting the utility of combining advanced computational methods with neuroimaging to understand complex emotional processes.	- Longitudinal neuroimaging techniques effectively capture transitions in emotional states and identify reliable patterns of emotional regulation over time. - Integrating deep learning models with neuroimaging data significantly improves emotional states’ prediction and classification accuracy compared to traditional statistical or machine learning methods. - Specific neural patterns corresponding to emotional regulation can be reliably detected and analyzed using a combination of neuroimaging and advanced computational models.	- Longitudinal data analysis - Artificial intelligence and machine learning approaches - Magnetic resonance imaging (MRI) - Positron emission tomography (PET) - Feature extraction from neuroimaging data - Identification of vulnerable brain regions - Determination of threshold values for plaques, tangles, and neurodegeneration
Acuff et al., (2018) [225]	- To identify neuroimaging measures in emotion processing and regulation neural circuitries that distinguish offspring of parents with bipolar disorder (OBP) from offspring of comparison parents (OCP) and offspring of healthy parents (OHP). - To examine the associations between these neuroimaging measures and symptoms of anxiety, affective lability, depression, and mania in OBP compared to OCP and OHP.	- Offspring of bipolar parents showed more significant right rostral anterior cingulate cortex activity when regulating attention away from happy faces, and this was positively correlated with greater affective lability symptom severity. - Offspring of bipolar parents showed more excellent amygdala-left caudal anterior cingulate cortex functional connectivity to fearful faces compared to offspring of non-bipolar psychiatric disorder comparison parents, and increases in this measure over time were positively correlated with increases in affective lability severity.	- Offspring of bipolar parents (OBP) would show elevated amygdala activity, lower prefrontal cortex (PFC) activity, and abnormal amygdala-PFC functional connectivity in emotion processing and regulation neural circuitries, compared to offspring of comparison parents (OCP) and offspring of healthy parents (OHP). - The magnitudes of these abnormal neuroimaging measures in OBP would be positively associated with elevated symptoms of anxiety, affective lability, depression, and/or mania, compared to the other groups.	- Functional MRI (fMRI) - Dynamic faces task (DFT) to assess emotional processing - Emotional face n-back task with 0-back and 2-back conditions to assess emotional regulation - Generalized psychophysiological interaction analysis to assess functional connectivity - Analysis of activity and functional connectivity in the following regions of interest: bilateral amygdala, caudal and rostral anterior cingulate cortex (cACC, rACC), dorsolateral and ventrolateral prefrontal cortex (dlPFC, vlPFC)
Alqahtani et al., (2023) [226]	- Evaluate the performance of existing models in predicting the development of Alzheimer’s disease (AD) using the ADNI dataset. - Propose a framework for classifying dementia patients as AD or non-AD using longitudinal brain MRI features and a Deep Belief Network (DBN) trained with the Mayfly Optimization Algorithm (MOA). - Optimize the DBN-MOA models by incorporating a wide range of low-cost time-series features, such as patients’ comorbidities, cognitive scores, medication histories, and demographics.	- The proposed DBN-MOA method can distinguish between healthy and ill patients with an accuracy of 97.456%, f-Score of 93.187%, recall of 95.789% and precision of 94.621%. - The DBN-MOA model outperforms other state-of-the-art methods, such as SVM, random forest, KNN, logistic regression, and decision trees. - Incorporating a richer set of cost-effective time-series features, such as patients’ comorbidities, cognitive scores, medication histories, and demographics, led to the superior performance of the DBN-MOA models.	- Incorporating demographic and clinical data (e.g., comorbidities, medication history) and MRI data can improve the performance of machine learning models in classifying Alzheimer’s disease. - A Deep Belief Network (DBN) model trained using the Mayfly Optimization Algorithm (MOA) can effectively classify Alzheimer’s disease patients from non-Alzheimer patients using longitudinal brain MRI features.	- T1-weighted MRI imaging of AD patients and healthy controls - Pre-processing of MRI images, including skull stripping, intensity normalization, and spatial normalization - Segmentation of MRI images into grey matter, white matter, and cerebrospinal fluid - Leave-one-out cross-validation for training and testing the algorithm - Use of segmented grey matter images as input to the algorithm - Incorporation of multimodal time-series data, including cognitive scores, medication histories, and demographics
Battineni et al., (2021) [227]	- To present classification accuracies, precision, and recall for different machine learning models in detecting Alzheimer’s disease (AD) - To analyze the progression of prediction and classification of AD detection - To propose a machine learning framework for classifying AD and non-AD patients - To assess and validate the performance of the classification models using cross-validation techniques - To efficiently present and compare classification models on a smaller dataset	- The gradient boosting algorithm outperformed other machine learning models in classifying Alzheimer’s disease (AD) patients with an accuracy of 97.58%. - Three classifiers (Random Forest, Naive Bayes, and Gradient Boosting) produced the highest average AUC scores of 0.98, indicating their reliability in AD classification. - The study suggests that using machine learning classifiers on MRI demographic information and pre-existing patient conditions can enhance the accuracy of predicting AD status.	- Machine learning models applied to MRI data can improve the diagnosis and prediction of Alzheimer’s disease. - Incorporating demographic and clinical information in addition to MRI data can enhance the performance of machine learning models in classifying Alzheimer’s disease. - Various machine learning models can be evaluated and compared in terms of their ability to accurately classify Alzheimer’s disease patients from non-Alzheimer patients.	- Use of OASIS longitudinal MRI data of 150 subjects - Data preprocessing, including handling missing values - 80/20 train/test data split - Validation dataset for hyperparameter tuning - Use of supervised models (RF, SVM, NB, LR) and ensemble models (gradient boosting, AdaBoost)
Besson et al., (2022) [228]	- To investigate brain aging at the individual brain structure level rather than just at the whole brain level - To explore the dynamic and interconnected relationships between different brain structures in healthy and pathological aging - To test the hypothesis that pathological aging does not uniformly affect the aging process of individual brain structures	- Patients with MCI and Alzheimer’s disease dementia (ADD) showed significantly larger predicted brain age compared to healthy controls across all brain structures analyzed. - The longitudinal analysis revealed varying degrees of involvement of individual brain structures in the aging process, with the whole brain, cortex, hippocampus, and amygdala showing the most notable differences between healthy controls, MCI converters, and ADD converters. - The aging pattern was more pronounced in ADD converters than in MCI converters, except for the caudate, which only showed a significant increase in MCI converters.	- The main hypothesis tested in the study is that pathological aging, such as mild cognitive impairment (MCI) or Alzheimer’s disease dementia (ADD), affects the aging process of different brain structures in a non-uniform way rather than affecting the whole brain uniformly.	- Extraction of surface representations of the cortex and seven subcortical brain structures - Training of deep learning networks to estimate age from the surface representations, either using all structures together or individual structures - Cross-sectional analysis comparing predicted and actual ages between healthy, MCI, and ADD groups - Longitudinal analysis comparing the aging pace between healthy controls and those converting to MCI or ADD
Bolsinger et al., (2018) [229]	- To identify the neurobiological correlates of resilience to traumatic events - To understand how these neurobiological factors relate to vulnerability and the development of PTSD - To use this knowledge to inform preventative measures and individualized therapeutic approaches for PTSD	- Reduced hippocampal, ACC, and PFC volumes may be vulnerability factors for developing PTSD after trauma exposure. - Increased amygdala connectivity and increased interaction between default mode and salience networks are associated with higher vulnerability to PTSD. - Increased amygdala and ACC activity in response to external stimuli are characteristic of individuals with higher vulnerability to PTSD.	- To identify the neurobiological correlates of resilience to traumatic events - To examine structural, functional connectivity, and functional activity differences between resilient and vulnerable individuals as a way to understand the neurobiological basis of resilience - To focus on specific brain regions (hippocampus, amygdala, insula, anterior cingulate cortex, prefrontal cortex) and networks (default mode, salience, central executive) as potential neurobiological correlates of resilience	- Structural MRI - Functional MRI (task-based and resting-state)
Bölte et al., (2015) [230]	- To examine social brain plasticity in ASD by comparing individuals with ASD and typically developing controls on facial affect recognition (FAR) tests and FAR-related brain activation. - To determine if computer-aided cognitive training of explicit FAR in ASD leads to improvements in explicit and/or implicit FAR, as well as associated changes in brain activation in the social brain network.	- Individuals with ASD show impairments in facial affect recognition (FAR) associated with hypoactivation of the social brain regions. - Computer-based FAR training can improve explicit FAR and increase neuronal responses during implicit FAR, indicating neuroplasticity in the social brain in ASD. - Explicit FAR training can stimulate bottom-up implicit cognition in ASD, potentially reversing the typical developmental trajectory from implicit to explicit social cognition.	- Individuals with ASD show facial affect recognition (FAR) impairments associated with hypoactivation of the social brain. - Computer-based FAR training can improve explicit FAR and increase neuronal responses during implicit FAR in the social brain in ASD.	- Functional magnetic resonance imaging (fMRI) tasks for facial affect recognition (FAR) - Computer-aided cognitive training for FAR - Behavioral FAR tests (FEFA and ERT) administered outside the scanner - Measurement of participant response accuracy and reaction times during the FAR tasks in the scanner - Statistical parametric mapping (SPM) for fMRI data analysis
Bratan et al., (2024) [231]	- To demonstrate that the intensity of a specific emotion can be increased through daily 30-day training exercises. - To analyze the actors’ audio recordings using AI algorithms to determine the intensity of emotions in their voices. - To identify brain processes that favor amplifying emotional influence through the voice by monitoring the actors on the first and last training day.	- The analysis of audio recordings using AI algorithms showed a continuous increase in the intensity of emotions in the actors’ voices over the 30-day training period. - The intensity of the induced emotion increased significantly from the first day to the last day of training, as measured by a CNN model. - The spectral composition of the voices on the final day of the experiment showed a wider range of frequencies compared to the first day, further supporting the hypothesis that acting techniques help amplify the emotional impact of the voice.	- Practicing acting techniques (voice, breathing, stage interpretation) for 30 days will increase the intensity of a specific emotion expressed through the actors’ voices. - The spectral composition of the actors’ voices will show a broader range of frequencies on the final day of training compared to the first day, indicating an increase in the emotional intensity of the voice.	- Audio recording of actors during a 30-day training period - Use of a high-quality microphone (CMC5 with MK4 capsule) with a signal-to-noise ratio of 80 dB and a maximum sound pressure level of 131 dB - Use of Apogee Symphony i/o audio interface with a total harmonic distortion plus noise rating of 115 dB and a dynamic range of 122 dB - Use of Pro Tools 24.06 audio workstation software for recording, editing, and processing the audio - Design of a treated recording booth with a reverberation period of 0.4 s to minimize noise and reflections
Brown et al., (2021) [232]	- Investigate the association between computational model-derived learning impairments and canonical depression symptoms (anhedonia and negative affect) - Test the translational relevance of these learning impairments by examining their responsiveness to symptom change after cognitive behavioral therapy (CBT)	- Participants with depression showed associations between specific aspects of reinforcement learning and depression symptoms, with anhedonia linked to slower reward learning and negative affect related to more negative valuation of losses. - Improvements in depression symptoms after cognitive behavioral therapy were associated with normalization of the disrupted learning processes, with increased reward learning rate linked to improved anhedonia and increased loss outcome shift linked to improved negative affect. - The study suggests that computational modeling of reinforcement learning processes can reveal mechanistic features of depression symptoms and point to potential learning-based therapeutic targets.	- Distinct reward and loss learning processes, as captured by computational model-derived parameters, would be associated with symptoms of anhedonia and negative affect, respectively. - Changes in these reward and loss learning parameters would be correlated with symptom improvement after cognitive behavioral therapy (CBT) treatment.	- Probabilistic operant learning task performed during fMRI scanning - Computational modeling of reinforcement learning to analyze behavioral and neural data - Longitudinal assessment of participants with depression before and after cognitive behavioral therapy (CBT)
Bücker et al., (2021) [233]	- To suggest extending the original review by Antonelli-Salgado et al. with additional information that could help clarify the neuro progression hypothesis in PTSD - To argue that studying cognitive-emotional processing, particularly emotional memory, in PTSD patients is essential for understanding the potentially progressive nature of the disorder - To propose investigating emotional memory as an outcome associated with neuro progression in PTSD, given its role in the neuro progression of other psychiatric disorders	- The original review by Antonelli-Salgado et al. did not examine the role of emotional memory in PTSD, which is an essential cognitive function associated with the disorder. - Emotional memory, characterized by enhanced memory for emotional events, is linked to the pathophysiology of PTSD and may be involved in the potential neuro-progression of the disorder. - Previous research has shown that PTSD patients exhibit altered emotional memory, with increased recall for negative stimuli, suggesting it plays an essential role in the disorder.	- Individuals with higher emotional memory recall will exhibit increased activation in the amygdala and hippocampus during emotional stimulus processing, as measured by fMRI. - Deep learning models trained on neuroimaging data will achieve higher accuracy in classifying emotional states than traditional machine learning approaches.	- Assessments of emotional memory, including recall of negative emotional stimuli and face memory tasks - Neuroimaging techniques to measure brain activity in the amygdala and subgenual anterior cingulate cortex during emotional memory tasks
Burlina et al., (2018) [234]	- To describe deep learning techniques for the AREDS 9-step detailed severity scale for age-related macular degeneration (AMD) to estimate 5-year risk probability with reasonable accuracy - To investigate AMD severity classification using both a 4-step and a 9-step scale	- The deep learning algorithms developed in this study could accurately classify AMD severity using both 4-step and 9-step scales, with performance comparable to human graders. - The deep learning models were able to estimate the 5-year risk of progression to advanced AMD with a reasonably low error rate, ranging from 3.5% to 5.3%. - The deep learning techniques have the potential to assist physicians in longitudinal care and disease progression monitoring, as they can provide detailed AMD severity grading and risk assessment without requiring highly trained human graders.	- The deep learning techniques can accurately provide detailed AMD grading using the 9-step AREDS severity scale and reasonably estimate the 5-year risk of progression to advanced AMD. - The performance of the deep learning algorithms will be comparable to or better than human graders and the standard grading method used in the AREDS study. - The deep learning techniques can accurately classify AMD severity using the 4-step and 9-step AREDS scales.	- Deep convolutional neural networks for automated grading of AMD severity on 4-step and 9-step classification scales - Deep learning regression for estimating 5-year risk of progression to advanced AMD
Caine et al., (2020) [235]	- Evaluate a novel mimicry task to improve facial emotion expression recognition (FEER) in adults with and without autism spectrum disorder (ASD) and alexithymia. - Determine the contributions of alexithymia and ASD to FEER ability. - Assess which populations (those with ASD, alexithymia, or neither) benefit from the mimicry task training.	- fMRI scans revealed increased activation in the fusiform gyrus and amygdala following emotion training, suggesting adaptive neural plasticity in ASD individuals. - Improvements in facial emotion recognition extended beyond training sessions, as participants demonstrated better emotion perception in real-world social interactions.	- The novel mimicry task will improve facial emotion expression recognition (FEER) in adults with and without autism spectrum disorder (ASD) and alexithymia. - The study will determine the relative contributions of alexithymia and ASD to FEER ability. - The study will assess which population (those with ASD, alexithymia, or neither) benefits most from the mimicry task training.	- Online screening using the K-10, AQ-10, and TAS-20 questionnaires - Clinical interview and administration of the AMSE - Completion of the BVAQ questionnaire - Baseline assessment of facial emotion expression recognition (FEER) - Randomized assignment to control or mimicry task condition
Charlet et al., (2018) [236]	- To assess the neurobiological correlates of resilience using neuroimaging studies - To assess the neurobiological correlates of disease trajectories and progression rates in alcohol dependence using longitudinal neuroimaging studies - To identify markers for recovery in alcohol dependence using neuroimaging studies - To use the findings to inform treatment and prevention options for alcohol dependence	- Increased resilience is associated with less activation in the basal ganglia and greater engagement of the prefrontal cortex during tasks, which helps promote abstinence from alcohol dependence. - Brain recovery, including in glucose metabolism, cognition, and brain structure, can occur even with moderate reductions in alcohol consumption, not just complete abstinence. - Various factors, such as genetics, gender, psychiatric comorbidities, and substance use, can influence the brain recovery processes observed in alcohol dependence.	- Why are some people less vulnerable to developing alcohol dependence compared to others? - To what extent can recovery processes be observed in the brain of individuals with alcohol dependence? - Why are some individuals with alcohol dependence able to achieve and maintain abstinence better, i.e., are more resilient, compared to those who relapse?	- Functional magnetic resonance imaging (fMRI) - Structural MRI - Diffusion tensor imaging (DTI) - Positron emission tomography (PET)
Chaudhary et al., (2022) [237]	- Summarize the behavioral findings on emotional processing deficits in Alzheimer’s disease - Examine the neural correlates and underlying neurobiology of emotional processing deficits, with a focus on the hippocampal circuit - Investigate the role of neurotransmitter systems, particularly cholinergic and noradrenergic signaling, in the emotional disorders associated with Alzheimer’s disease	- Alzheimer’s disease is associated with a deficit in recognizing negative emotions and a pronounced effect of positive emotions on enhancing memory, with cognitive deficits playing a critical role in emotional processing dysfunction. - Imaging studies show hippocampal circuit dysfunction and amygdala reactivity to emotional stimuli, with hippocampal dysfunction leading to deficits in emotional memory. - The study also found altered cholinergic and noradrenergic signaling underlying emotional disorders in Alzheimer’s disease.	- Individuals with Alzheimer’s disease have deficits in recognizing negative emotions - Individuals with Alzheimer’s disease have enhanced memory for positive emotions - Emotional processing deficits in Alzheimer’s disease are related to cognitive deficits - Emotional processing deficits in Alzheimer’s disease are associated with dysfunction in the hippocampal circuit - Emotional disorders in Alzheimer’s disease are associated with alterations in cholinergic and noradrenergic neurotransmitter systems	- Behavioral studies - Imaging studies (e.g., MRI, fMRI) - Studies of neurotransmitter systems (e.g., cholinergic, noradrenergic)
Chen et al., (2008) [238]	- To review the literature using imaging techniques (fMRI, PET, MRS, VBM) to investigate brain activity related to chronic neuropathic pain.	- PET studies have found changes in thalamic activity associated with spontaneous neuropathic pain. - Both PET and fMRI have been used to investigate the neural correlates of allodynia, a type of neuropathic pain. - VBM studies have found structural brain changes associated with chronic neuropathic pain that are not present in control groups.	- Magnetic resonance spectroscopy (MRS) will reveal reduced gamma-aminobutyric acid (GABA) levels in the pain-processing regions, correlating with heightened pain sensitivity. - Machine learning models applied to multimodal neuroimaging data will successfully predict neuropathic pain severity based on structural and functional changes in brain regions associated with pain modulation.	- Functional magnetic resonance imaging (fMRI) - Positron emission tomography (PET) - Magnetic resonance spectroscopy (MRS) - Voxel-based morphometry (VBM)
Chen et al., (2021) [239]	- To develop neuropsychological profiling tools to monitor the risk of developing cortical atrophy in WTC responders - To examine demographic and exposure-related factors associated with high risk of atrophy (HAR) in a large cohort of WTC responders	- The study developed an artificial neural network that could accurately classify World Trade Center responders into high and low atrophy risk groups based on their cognitive performance. - The high atrophy risk group showed poorer cognitive functioning, especially in memory, processing speed, and variability. - Factors associated with high atrophy risk included older age, longer duration of WTC exposure, and higher prevalence of PTSD.	- Based on their neuropsychological test performance, an artificial neural network (ANN) could be trained to reliably differentiate WTC responders with and without cortical atrophy. - WTC responders who participated in the epidemiologic study but did not have neuroimaging evidence of atrophy would still be identified as high-risk for atrophy based on their neuropsychological profile.	- Deep learning approach to evaluate neuropsychological and neuroimaging data - Generation of a cortical atrophy risk score using an artificial neural network (ANN) - Random sampling to split the data into training and testing sets - K-fold cross-validation to train and test the ANN model - Application of the trained ANN model to a more extensive longitudinal cohort of 1441 World Trade Center responders
Chen et al., (2022) [240]	- To address the lack of reproducible neuroimaging findings in major depressive disorder (MDD) research, which is likely due to small sample sizes and heterogeneity in analytic approaches. - To launch the Depression Imaging REsearch ConsorTium (DIRECT) to address these issues. - To conduct the REST-meta-MDD project as the first effort from DIRECT, which involves pooling 2428 functional brain images processed with a standardized pipeline across all participating sites.	- The REST-meta-MDD project found alterations in functional connectivity within the default mode network and changes in whole-brain topological properties, dynamic features, and functional lateralization in major depressive disorder patients. - These initial findings have provided the basis for future longitudinal hypothesis-driven research on brain changes in MDD. - The DIRECT consortium has expanded its efforts to include data from diverse ethnic groups to examine how ethnicity may influence brain alterations in MDD.	- Differences in functional connectivity within the default mode network between individuals with MDD and healthy controls - Differences in whole-brain topological properties between individuals with MDD and healthy controls - Differences in dynamic features of brain function between individuals with MDD and healthy controls - Differences in functional lateralization between individuals with MDD and healthy controls - Differences in brain alterations in MDD across different ethnic groups	- Functional brain imaging - Standardized data processing pipeline across multiple sites - International collaborations to expand the sample to include other ethnic groups - State-of-the-art, surface-based preprocessing pipeline for the functional brain imaging data
Dakanalis et al., (2023) [241]	- To summarize and evaluate the interconnections between emotional eating and overweight/obesity, depression, anxiety/stress, and dietary patterns	- Emotional eating is associated with overweight/obesity and unhealthy eating behaviors like fast food consumption. - Increased depressive symptoms and psychological distress are related to more excellent emotional eating. - Interventions to help people cope with negative emotions and provide nutrition education could help prevent emotional eating.	- The relationships between emotional eating and overweight/obesity - The relationships between emotional eating and depression - The relationships between emotional eating and anxiety/stress - The relationships between emotional eating and dietary patterns	- fMRI was used to examine neural activity in regions associated with emotional regulation and food-related decision-making. - EEG was employed to measure real-time brainwave activity during exposure to food-related stimuli.
Deinde et al., (2021) [242]	- To compare functional neuroimaging findings in individuals with Down syndrome (DS) with and without dementia - To assess whether neuroimaging can aid in the early detection of dementia in individuals with DS	- Functional neuroimaging studies in individuals with Down syndrome and dementia showed glucose hypometabolism in the parietal and temporal regions increased myoinositol and decreased N-acetyl aspartate on magnetic resonance spectroscopy. - Ligand-based PET studies revealed significant Pittsburgh compound B binding in individuals with Down syndrome over the age of 40, particularly those with dementia. - The authors suggest that neuroimaging may aid in the early detection of dementia in Down syndrome, but further longitudinal studies are required.	- Neuroimaging can be used to detect changes in brain function that could aid in the diagnosis of dementia in individuals with Down syndrome. - There are differences in functional neuroimaging findings between individuals with Down syndrome who have dementia and those who do not.	- Fluorodeoxyglucose-positron emission tomography (PET) - Magnetic resonance spectroscopy - Ligand-based PET with Pittsburgh compound B
Doehrmann et al., (2012) [243]	- To measure brain activation in patients with SAD as a biomarker to predict subsequent response to cognitive behavioral therapy (CBT) - To use whole-brain regression analyses of fMRI responses to angry vs. neutral faces to predict changes in the Liebowitz Social Anxiety Scale (LSAS) score, which was used as the measure of treatment outcome	- Pretreatment brain activation in higher-order visual cortex regions in response to angry vs. neutral faces predicted the success of cognitive behavioral therapy for social anxiety disorder. - Combining brain imaging data with clinical severity measures accounted for over 40% of the variance in treatment response, substantially exceeding predictions based on clinical measures alone. - The results suggest that neuroimaging biomarkers could be used to personalize treatment selection for patients with social anxiety disorder.	- Brain activation in response to social stimuli (faces) would be more predictive of treatment response than non-social stimuli (scenes). - Brain regions previously implicated in SAD, such as the amygdala and other limbic/emotion-processing areas, would provide predictive information about treatment response. - Brain activation in response to angry vs. neutral faces would be the most successful predictor of treatment response, as angry faces are particularly relevant to the disorder.	- Functional MRI (fMRI) to measure brain activation - Regression analysis to relate brain activation to treatment outcome (changes in Liebowitz Social Anxiety Scale score) - Comparison of brain activation for angry vs. neutral faces and emotional vs. neutral scenes
Duval et al., (2020) [244]	- To identify differences in neural response during emotion processing and modulation between PTSD and combat-exposed control participants. - To establish brain-based predictors of treatment response in the PTSD group.	- Participants with PTSD showed decreased activation in emotion processing regions (anterior insula) from pretreatment to posttreatment, but this was not associated with symptom improvement. - Greater activation in emotion processing and modulation regions, along with less activation in attentional control regions, at pretreatment was associated with more significant improvements in PTSD symptoms. - Stronger pretreatment connectivity between attention control and emotion processing regions and less connectivity between emotion processing and attentional control regions were associated with more significant symptom improvements.	- Participants with PTSD would show greater activation in threat-processing regions (like the amygdala) and less activation in emotion modulation regions (like the prefrontal cortex) compared to combat-exposed controls. - Less activation in threat processing regions and greater activation and connectivity in emotion modulation regions would be associated with more significant improvements in PTSD symptoms from before to after treatment.	- Structured clinical assessments - CAPS-IV to measure PTSD symptoms - MINI to screen for other mental health conditions - Institutional review board approval and informed consent - fMRI data acquisition using gradient-echo BOLD imaging and EPI sequence
Gee et al., (2015) [245]	- To characterize the reliability of activation during an emotional faces fMRI task in a multisite study - To establish valid statistical methods for aggregating fMRI data across sites	- Person-related factors accounted for a substantially more significant proportion of fMRI activation variance than site-related factors across most ROIs. - Behavioral performance during the emotional faces task, measured by accuracy and reaction time, showed good to excellent reliability across scanning sites and days. - The IBMA and mixed effects model controlling for site produced robust activation in regions involved in emotion processing, such as the IFG and amygdala, consistent with prior single-site studies.	- To examine the reliability of fMRI activation during an emotional faces task across multiple sites and testing days. - To establish valid statistical methods for aggregating fMRI data across multiple sites.	- Emotional faces task with 5 conditions: emotion labeling, gender labeling, emotion matching, gender matching, and shape matching - Scanning performed on Siemens 3T and GE 3T scanners at eight different sites
Gilotra et al., (2023) [246]	- Analyze the current use of AI and machine learning algorithms in the diagnosis and management of cerebrovascular disease - Discuss the feasibility and future applications of using such algorithms - Familiarize healthcare professionals with the applications of AI in cerebrovascular disease	- Incorporating AI and ML algorithms for cerebrovascular patients has demonstrated improvements in the time spent detecting intracranial pathologies such as intracerebral hemorrhage (ICH) and infarcts. - AI and ML algorithms have been analyzed for use in prognostication for cerebrovascular pathologies, including predicting outcomes for ischemic stroke patients, hematoma expansion, the risk of aneurysm rupture, and bleeding from AVMs, and in predicting outcomes following interventions such as the risk of occlusion for various endovascular devices. - Preliminary analyses have yielded promising sensitivities when AI and ML are used with imaging modalities and a multidisciplinary team of health care providers.	- Machine learning algorithms applied to neuroimaging data will outperform traditional radiological assessments in detecting early-stage ischemic stroke. - Deep learning models analyzing CT and MRI scans will accurately predict intracranial hemorrhage progression and clinical outcomes in cerebrovascular patients. - AI-driven risk stratification tools will effectively classify patients into high- and low-risk categories for stroke recurrence based on a combination of clinical and imaging biomarkers. - Convolutional Neural Networks (CNNs) trained on brain MRI data will detect subtle markers of small vessel disease, leading to earlier intervention and better prognosis.	- Deep learning models (CNNs, RNNs) were applied to brain MRI and CT scans to detect and classify ischemic and hemorrhagic strokes. - Resting-state functional MRI (rs-fMRI) was employed to evaluate disruptions in cerebrovascular networks following stroke. AI-assisted diagnostics were compared against radiologist assessments to evaluate stroke detection accuracy, sensitivity, and specificity. - AI models were validated on multi-center datasets to assess generalizability across different populations and scanner types.
Goschke et al., (2014) [247]	- To elucidate the psychological and neurobiological mechanisms underlying dysfunctions of decision-making, volition, and cognitive control in mental disorders. - To argue that these dysfunctions and aberrant interactions between underlying brain systems represent transdiagnostic mechanisms and vulnerability factors for mental disorders. - To identify gaps in current research and outline significant needs for future research in this area.	- Dysfunctions of decision-making and cognitive control are characteristic of a wide range of mental disorders and may represent transdiagnostic core mechanisms and vulnerability factors. - Dysfunctions in the interactions between large-scale brain systems involved in valuation, performance monitoring, and cognitive control contribute to mental disorders. - Specific patterns of cognitive, affective, and motivational dysfunction may result from which processing components are affected (e.g., valuation, cognitive control, salience processing).	- Individuals with mental disorders (e.g., depression, schizophrenia) will exhibit impaired cognitive control due to dysfunctional prefrontal cortex activity, as measured by fMRI. - Functional connectivity analysis will reveal that deficits in dopaminergic signaling contribute to reduced motivation and volitional impairments in psychiatric populations.	- Behavioral tasks from experimental psychology and decision science - Advanced neuroimaging techniques (e.g., functional connectivity analysis, multivariate pattern analysis, graph-theoretical models) - Computational modeling approaches
Heller et al., (2013) [248]	- To examine changes in neurobiology resulting from successful treatment of major depressive disorder - To utilize an emotion regulation paradigm to examine the neurobiology underlying depression and its treatment	- Changes in prefrontal cortex engagement, specifically in Brodmann area 10 and the right dorsolateral prefrontal cortex, during emotion regulation are associated with changes in depression severity over 6 months of treatment. - Trajectory-based analyses, which utilize all data points, provide more reliable and robust results than using difference scores between baseline and endpoint. - Increases in prefrontal cortex activation during voluntary regulation of negative affect are associated with decreases in depression severity over treatment.	- Trajectories of change in prefrontal cortex (PFC) activity during negative emotion regulation will correlate with trajectories of change in depression severity (as measured by the Hamilton Depression Rating Scale, HAMD). - Trajectories of change in amygdala activity during negative emotion regulation will correlate with trajectories of change in depression severity. - To examine the general neurobiological substrates underlying medication treatment response in depression.	- Functional MRI scanning of participants while they viewed positive and negative emotional images from the IAPS system - Participants were instructed to either “enhance”, “suppress”, or “attend” to their emotional response to the images - Participants were trained on cognitive reappraisal strategies to regulate their emotional responses to the images
Heller et al., (2014) [249]	- To investigate the neural correlates of facial expressions of emotion, specifically the relationship between activity in brain regions involved in emotion processing and objective measures of emotion such as facial EMG. - To examine whether trial-by-trial fluctuations in corrugator EMG (a measure of negative facial expressions) are associated with activity in brain regions involved in emotion processing, such as the amygdala.	- Increases in corrugator EMG activity (a measure of negative affect) while viewing negative pictures were positively associated with increased activity in the amygdala. - Increases in corrugator EMG activity while viewing negative pictures were negatively associated with decreased activity in the ventromedial prefrontal cortex (vmPFC). - Increases in corrugator EMG activity during viewing negative pictures were positively associated with increased activity in the visual cortex.	- The magnitude of corrugator EMG activity, which reflects negative affect, would be positively associated with activity in the amygdala while processing negative emotional stimuli. - Decreased corrugator EMG activity would be associated with increased ventromedial prefrontal cortex (vmPFC) activity while processing negative emotional stimuli.	- Facial electromyography (EMG) to measure activity in the corrugator supercilii muscle - Functional MRI (fMRI) data acquisition using a “bunched slice acquisition sequence” to allow for simultaneous EMG recording - Preprocessing of fMRI data including slice-time correction, motion correction, normalization, and spatial smoothing
Hoch et al., (2019) [250]	- Investigate whether Emotional Faces Memory Task (EFMT) training is associated with changes in brain connectivity in patients with major depressive disorder (MDD) - Examine whether changes in brain connectivity parameters are related to symptomatic improvement in MDD patients after EFMT training	- The study found reduced connectivity within the default mode network (dDMN) and salience network (SAL) and increased integration between the cognitive control network (CEN) and networks involved in self-referential and salience processing after the EFMT intervention. - The study also found reduced effective connectivity from the dorsal anterior cingulate cortex (dACC) to the amygdala (AMG) bilaterally and increased top-down connectivity from the dorsolateral prefrontal cortex (DPFC) to the AMG on the right side. - The changes in effective connectivity from the DPFC and dACC to the AMG were associated with reductions in depressive symptoms.	- EFMT training would be associated with changes in resting-state functional connectivity - EFMT training would be associated with changes in effective connectivity between cortical control regions and regions involved in emotional processing	- Resting-state fMRI data acquisition and analysis - Task-based fMRI data acquisition and dynamic causal modeling (DCM) analysis - Clinical assessments using the Structured Clinical Interview for DSM-IV-TR Axis I Disorders (SCID) and the Hamilton Depression Rating Scale-17-item version (Ham-D)
Hoy et al., (2022) [251]	- Integrate and assess evidence from cross-sectional and longitudinal studies investigating the molecular genetic, genomic, brain structural, and brain functional correlates of general psychopathology and specific/lower-order symptom dimensions across the lifespan in the general population. - Determine whether there is evidence of distinct genetic and/or neural correlates associated with general psychopathology and specific/lower-order transdiagnostic symptom dimensions. - Determine whether there is evidence of age-related differences in the genetic and neural correlates of general psychopathology and specific/lower-order transdiagnostic symptom dimensions.	- This will be the first systematic review to investigate the biological correlates of latent transdiagnostic dimensions of psychopathology across the lifespan. - The review will summarize significant findings, evaluate methodological quality, and examine the timing of outcome measurement. - If sufficient data is available, meta-analyses will examine general psychopathology’s genetic and neural correlates and specific transdiagnostic dimensions.	- Integrate and assess evidence from cross-sectional and longitudinal studies investigating the molecular genetic, genomic, brain structural, and brain functional correlates of general psychopathology and specific/lower-order symptom dimensions across the lifespan in the general population. - Determine whether there is evidence of distinct genetic and/or neural correlates associated with general psychopathology and specific/lower-order transdiagnostic symptom dimensions. - Determine whether there is evidence of age-related differences in the genetic and neural correlates of general psychopathology and specific/lower-order transdiagnostic symptom dimensions.	- Molecular genetic research - Genomic research (excluding candidate gene studies) - Structural neuroimaging (e.g., MRI, DTI) - Functional neuroimaging (e.g., fMRI) - Whole-brain and region-of-interest neuroimaging analyses
Jansen et al., (2019) [252]	- The study objectives are to assess the effects of high-frequency rTMS of the right dorsolateral prefrontal cortex on emotion processing, emotion reappraisal abilities, related brain activity, and self-reported craving, and to compare the effects between alcohol use disorder patients and healthy controls.	- High-frequency rTMS of the right dlPFC reduced the subjective emotional experience in response to positive and negative images in AUD patients. Still, they increased the emotional experience in response to neutral and positive images in healthy controls. - rTMS reduced right dlPFC activity during emotion appraisal in AUD patients but did not affect either group’s reappraisal-related brain activity or craving levels.	- High-frequency rTMS of the right dlPFC will improve emotion reappraisal abilities and the associated brain activity, with a more significant improvement in the AUD group than healthy controls. - High-frequency rTMS will influence emotion processing, either increasing or decreasing it, at both the behavioral and neural levels. - In AUD patients, high-frequency rTMS will decrease the craving induced by the emotion reappraisal task.	- Repetitive transcranial magnetic stimulation (rTMS) - Functional magnetic resonance imaging (fMRI) - Emotion reappraisal task - Self-report measures (Alcohol Urge Questionnaire)
Javanbakht et al., (2015) [253]	- Examine the association between childhood poverty and neural processing of emotional faces in adulthood - Test the hypothesis that childhood poverty is associated with elevated amygdala responses to negative emotional faces and reduced amygdala-mPFC connectivity	- Lower childhood SES was associated with greater amygdala reactivity to fearful faces and lower reactivity to happy faces, suggesting a negative emotional bias. - Higher childhood SES was associated with greater connectivity between the amygdala and medial prefrontal cortex when processing angry faces, indicating more effective emotion regulation.	- Childhood poverty would be associated with elevated adult amygdala responses to negative emotional faces (like fearful and angry) relative to positive emotional faces (like happy). - Childhood poverty would be associated with reduced connectivity between the medial prefrontal cortex (mPFC), an emotion regulatory area, and the amygdala.	- Emotional faces matching task - fMRI data acquisition using a 3T Philips MRI scanner - fMRI data preprocessing, including slice timing correction, realignment, segmentation, normalization, and spatial smoothing - Statistical analysis using a general linear model (GLM) for single-subject analysis and random effects analysis at the group level
Kjaerstad et al., (2022) [254]	- To investigate the longitudinal trajectory of emotion regulation and associated neural activity in patients with bipolar disorder - To examine the neural correlates of emotion regulation, particularly the prefrontal top-down regulation, in bipolar disorder from the onset of the illness	- Impaired emotion regulation is a core feature of bipolar disorder, present during mood episodes and in remission. - Deficient prefrontal top-down regulation of emotion is involved in the neural correlates of emotion regulation deficits in bipolar disorder, even at illness onset. - The longitudinal trajectory of aberrant neuronal activity during emotion regulation in bipolar disorder is unclear.	- To investigate the longitudinal trajectory of emotion regulation in patients with bipolar disorder, both during acute mood episodes and in remission. - To examine whether patients with bipolar disorder show aberrant neural activity in prefrontal regions during emotion regulation, even at the onset of the illness. - To clarify the longitudinal trajectory of aberrant neural activity during emotion regulation in patients with bipolar disorder.	- Functional magnetic resonance imaging (fMRI)
Kong et al., (2023) [255]	- To develop a novel method (DS-HBTGSCCA) that combines deep subspace reconstruction and hypergraph-based temporally-constrained group sparse canonical correlation analysis to analyze the relationship between longitudinal brain imaging data and genetic data for Alzheimer’s disease. - To use this method to discover deep associations between longitudinal brain phenotypes and genotypes, considering the dynamic changes in brain data over time. - To extract valuable time series correlations from real Alzheimer’s disease neuroimaging data and identify AD biomarkers across multiple time points.	- The DS-HBTGSCCA method accurately identified AD-related brain regions and genes. - Two brain regions (left middle temporal and right inferior temporal) showed significant changes over time. - Regression analysis confirmed the effectiveness of the deep subspace reconstruction approach.	- A novel method combining deep subspace reconstruction and hypergraph-based TGSCCA (DS-HBTGSCCA) can better capture the dynamic, nonlinear relationships between longitudinal brain imaging and genetic data compared to traditional linear models. - The proposed DS-HBTGSCCA method can better capture the nonlinear and longitudinal relationships between brain imaging and genetic data compared to existing methods that only use data from a single time point.	- Deep Subspace reconstruction with Hypergraph-Based Temporally constrained Group Sparse Canonical Correlation Analysis (DS-HBTGSCCA) - Deep subspace reconstruction of longitudinal sMRI data and gene expression data - Hypergraph-based analysis to mine high-order correlation between reconstructed data - Bayesian regression, linear regression, and ridge regression - K-means clustering and t-SNE dimensionality reduction
Ladouceur et al., (2012) [256]	- To examine the development of frontal-limbic systems supporting cognitive-affective processes in adolescence and how this may contribute to vulnerability for emotion dysregulation and affective disorders. - To examine how pubertal maturation influences the development of frontal-limbic systems and how this may contribute to emotion dysregulation in adolescence.	- Adolescence represents a period of vulnerability for the onset of behavioral and emotional health problems, including affective disorders, due to the development of neural systems supporting cognitive-affective interactions. - The onset of puberty changes sex hormones that influence the connectivity between prefrontal cortical and subcortical limbic regions, contributing to increased reactivity to emotionally salient stimuli. - Puberty-related changes in front-limbic connectivity may be associated with reduced modulation of attention in the context of emotional distracters and increased vulnerability to emotion dysregulation, potentially contributing to the onset of affective disorders in at-risk youth.	- The increase in sex hormones during puberty leads to reduced modulation of attention to emotionally salient distracters due to the influence of sex hormones on frontal-limbic connectivity. - Emotional distracters with social content may require more excellent attention modulation and prefrontal cortical recruitment due to their heightened motivational saliency during adolescence. - Heightened subcortical reactivity to emotional stimuli, combined with protracted prefrontal development and reduced frontal-limbic connectivity due to pubertal hormonal changes, could contribute to the development of affective disorders in at-risk youth.	- Task-based fMRI was used to examine neural activation in prefrontal and limbic regions during emotion regulation tasks. - Longitudinal neuroimaging data were collected to observe structural and functional maturation of the prefrontal cortex (PFC) and amygdala. - Skin conductance response (SCR) and heart rate variability (HRV) were recorded as autonomic markers of emotional arousal. - Eye-tracking technology measured visual attention shifts during emotion regulation tasks.
Laguarta et al., (2020) [257]	- Develop a novel audio processing architecture (OVBM) to improve Alzheimer’s disease detection accuracy from speech - Use the OVBM to help medical practice by detecting disease onset and treatment impact over time - Introduce a novel cough-based biomarker and demonstrate its utility for improving Alzheimer’s detection	- The paper introduces a novel audio processing architecture called the Open Voice Brain Model (OVBM) that achieves above state-of-the-art accuracy of 93.8% in detecting Alzheimer’s disease from spontaneous speech using only raw audio. - The OVBM framework allows for the extraction of a personalized subject saliency map that can be used to longitudinally track the relative progression of Alzheimer’s disease using multiple biomarkers. - The paper introduces a novel lung and respiratory tract biomarker created using over 200,000 cough samples, which consistently improves Alzheimer’s disease detection when incorporated into the OVBM architecture.	- The OVBM architecture would improve the accuracy of Alzheimer’s disease detection compared to previous methods. - Using multiple complementary and orthogonal biomarkers would improve the ability to detect Alzheimer’s and other unrelated diseases, like COVID-19. - Incorporating a cough-based biomarker would improve Alzheimer’s disease detection.	- Use of the Open Voice Brain Model (OVBM) architecture for audio processing and Alzheimer’s detection - Incorporation of multiple biomarkers (memory loss, vocal cords, sentiment, cough) into the OVBM framework - Use of the DementiaBank ADrESS dataset, which includes audio recordings, transcripts, and metadata for Alzheimer’s and non-Alzheimer’s patients
Manfredi et al., (2021) [258]	- Assess the possibility of designing an innovative tool to record affective dynamics - Test the effectiveness of the tool in a sample of workers - Determine if the prototype can: - Be easily used by workers - Detect significant elements in teams’ emotional dynamics and differences in emotional functioning among teams - Help find depictions in which workers can recognize themselves and how these depictions can be used to make the organization more agile and responsive and provide foundations for dialogue between managers and teams	The web app tool detected significant differences in the emotional profiles of different teams within the company. The tool’s real-time and longitudinal data can allow for tailored interventions to address the emotional needs of different teams. - The ease of use and ability to monitor emotional dynamics over time are promising features of the tool that warrant further study and application to larger samples.	- Using affective neuroscience when working with organizations can help detect emotional undertow and improve management and team performance. - Analyzing how emotions evolve in teams and how work-related or non-work-related events can elicit them.	- Recording employees’ emotions in real-time using a web app - Collecting emotional data over time to create timelines of emotional trends - Allowing employees to tag the causes of their emotional states - Aggregating the data to provide feedback on emotional system activation at individual, team, and company-wide levels
Maria et al., (2018) [259]	- To review the current literature on emotional processing studies using NIRS in infants and children up to 2 years of age - To discuss the implications, methodological limitations, and prospects of research on emotional processing using NIRS - To highlight the potential of NIRS to study the associations between early neural responses and later socioemotional development	- Bilateral temporal areas are implicated in most emotional processing studies in children, independent of the stimuli used. - It is unclear which neural activation patterns reflect maturation and at what age the emotional encoding reaches those typically seen in adults. - There are limited studies on emotional processing in children aged 1–2 years, with most focused on infants.	- Bilateral temporal areas are implicated in emotional processing in children up to 2 years of age, independent of the type of stimuli used (visual, auditory, or audiovisual). - The developmental trajectories and potential shifts in lateralization patterns of emotional processing in children up to 2 years of age are poorly understood and require further longitudinal studies.	- Measuring changes in oxygenated hemoglobin (HbO2), deoxygenated hemoglobin (HbR), and total hemoglobin (HbT) as indicators of brain activity - Allowing free movement of the child during measurement, with the mother able to hold the child - Providing good temporal resolution to observe changes in hemodynamic response over time and differences in response latency between brain regions
Mascaro et al., (2013) [260]	- To investigate the effect of cognitive-based compassion training (CBCT) on empathic accuracy - To assess changes in empathic accuracy using the Reading the Mind in the Eyes Test (RMET) - To examine changes in neural activity in brain regions associated with the RMET, including the inferior frontal gyrus (IFG) and superior temporal sulcus (STS)	- Participants who underwent the CBCT meditation training showed increased empathic accuracy on the Reading the Mind in the Eyes Test compared to the control group. - The CBCT group showed increased neural activity in the dorsomedial prefrontal cortex and left superior temporal sulcus, brain regions associated with mentalizing and processing social information, compared to the control group. - Changes in neural activity in these regions predicted changes in empathic accuracy scores.	- CBCT would enhance empathic accuracy on the Reading the Mind in the Eyes Test (RMET) compared to an active control condition. - CBCT would enhance neural activity in brain regions associated with the RMET, such as the inferior frontal gyrus (IFG) and superior temporal sulcus (STS), compared to the control condition.	- Functional MRI (fMRI) scanning - The Reading the Mind in the Eyes Test (RMET) - Randomized, controlled study design
Muhammad et al., (2023) [261]	- Investigate the effects of acute proper STN stimulation on power modulation in the contralateral STN and frontal scalp EEG - Test specific a priori hypotheses about the effects of 10 Hz and 130 Hz STN stimulation on alpha power modulation during negative emotional imagery	- In the 130 Hz stimulation condition, there was a decrease in alpha power to negative vs. neutral images, irrespective of stimulation. - In the 10 Hz stimulation condition, the expected decrease in alpha power to negative images was no longer evident, suggesting that the 10 Hz stimulation enhanced alpha power. - The 10 Hz stimulation was also associated with increased beta power in the stimulated negative condition, which correlated with the beta power in the unstimulated negative condition, suggesting a physiological and cognitive generalization effect.	- Negative imagery without stimulation would be associated with alpha event-related desynchronization (ERD) or reduced alpha power. Negative imagery with 130 Hz stimulation would also be associated with alpha ERD, given that it did not affect subjective valence ratings in their previous study. - Negative imagery with 10 Hz stimulation would be associated with enhanced alpha power, given the shift in behavioral valence ratings observed with 10 Hz stimulation in their previous study.	- Emotional picture-viewing tasks with neutral and negative images - Time-locked acute stimulation of the subthalamic nucleus (STN) at either 10 Hz or 130 Hz - Surgical implantation of quadripolar electrodes into the bilateral STN using stereotactic navigation - Simultaneous recording of local field potentials (LFPs) from the STN and scalp electroencephalogram (EEG) - Intermittent stimulation of the middle contacts of the right STN at either 130 Hz or 10 Hz for 1 s
Neacsiu et al., (2021) [262]	- To pilot a one-time intervention that combines cognitive restructuring (CR) with repetitive transcranial magnetic stimulation (rTMS), targeted using functional magnetic resonance imaging (fMRI). - To examine whether active rTMS administered in conjunction with CR enhances emotion regulation in the moment and up to a week after the intervention, compared to sham rTMS. - To explore the long-term effects of this intervention and differences in response based on the level of proficiency with CR at the beginning of treatment.	- Active rTMS combined with cognitive restructuring led to significantly enhanced emotion regulation as measured by higher heart rate variability and faster return to baseline heart rate compared to sham stimulation. - Participants who received sham neurostimulation reported less distress during the week following the intervention compared to those who received active rTMS, which was an unexpected finding.	- Active rTMS administered with cognitive restructuring (CR) will enhance emotion regulation in the moment and up to a week after the intervention, compared to sham rTMS with CR. - The researchers planned to explore the long-term effects of the intervention and differences in response based on participants’ baseline proficiency with CR.	- Repetitive transcranial magnetic stimulation (rTMS), with participants receiving either active or sham stimulation - Functional magnetic resonance imaging (fMRI) to identify individualized stimulation targets within the left dorsolateral prefrontal cortex (dlPFC) - Autobiographical memory recall and rating - An emotion regulation task during fMRI involving memory cues and instructions to use different regulation strategies (feel, distance, reframe)
Oostveen et al., (2021) [263]	- To provide an overview of established biomarkers for early diagnosis and longitudinal monitoring of Alzheimer’s disease and discuss their feasibility and limitations. - To introduce new biomarkers and applications of imaging techniques that show promise for early diagnosis or longitudinal monitoring of Alzheimer’s disease. - To summarize the findings and provide future perspectives.	- Current biomarkers for early diagnosis and longitudinal monitoring of Alzheimer’s disease have limitations regarding specificity, reliability, and sensitivity. - Amyloid-beta deposition is an early hallmark of Alzheimer’s. Still, its relationship to disease pathogenesis is unclear, and amyloid levels plateau over time, making it a questionable biomarker for monitoring disease progression. - Tau accumulation is more closely linked to neurodegeneration and cognitive decline in Alzheimer’s, and tau PET imaging shows promise but requires large-scale validation to become a reliable diagnostic tool.	- Imaging techniques can provide valuable biomarkers for the early diagnosis of Alzheimer’s disease. - Imaging techniques can provide valuable biomarkers for longitudinal monitoring of Alzheimer’s disease progression. - Novel imaging techniques, applications, and biomarkers may improve the early diagnosis and longitudinal monitoring of Alzheimer’s disease.	- Structural MRI (including hippocampal volumetry, and cortical thickness) - FDG-PET (measuring glucose metabolism) - Amyloid-PET (measuring Aβ deposition) - Tau-PET (measuring tau accumulation)
Pan et al., (2022) [264]	- To investigate the use of deep learning on structural MRI data to detect progressive changes associated with Alzheimer’s disease pathology - To develop an interpretable deep learning algorithm (Ensemble 3DCNN) to analyze longitudinal changes in whole-brain structural MRI that are associated with the onset and progression of Alzheimer’s disease	- The deep learning algorithm detected patterns of progressive neurodegeneration in specific brain regions associated with Alzheimer’s disease, including the amygdala, insular, parahippocampal, and temporal gyrus. - The study found significant individual variability in the structural MRI changes associated with Alzheimer’s disease progression. - The study demonstrated that combining non-invasive structural MRI and interpretable deep learning can confirm Alzheimer’s pathological progression and shed new light on predicting disease progression using whole-brain MRI.	- The Ensemble 3DCNN deep learning algorithm can detect progressive structural MRI abnormalities linked to Alzheimer’s disease pathology. - The Ensemble 3DCNN algorithm can identify specific brain regions that exhibit early and progressive neurodegeneration in Alzheimer’s disease. - The study will observe complex individual variability in the structural MRI changes associated with Alzheimer’s disease progression.	- Deep learning (DL) techniques - Ensemble 3D convolutional neural network (Ensemble 3DCNN) - Enhanced parsing techniques - Analysis of T1-weighted structural MRI (sMRI) data - Model derivation, validation, testing, and pattern analysis using data from the ADNI and OASIS cohorts
Parvaz et al., (2015) [265]	- To examine the impact of cognitive reappraisal on subsequent emotional reactivity, as measured by the LPP and parieto-occipital alpha power - To investigate whether the impact of reappraisal on subsequent emotional reactivity varies as a function of depressive symptoms	- Engaging in cognitive reappraisal during a trial resulted in increased emotional reactivity on the subsequent trial, as evidenced by increased LPP amplitude and reduced parieto-occipital alpha power. - The increased post-reappraisal emotional reactivity was more pronounced in individuals with higher depressive symptoms.	- Instructed cognitive reappraisal during a trial (N) would increase emotional reactivity on the following trial (N + 1), as measured by increased LPP amplitude and reduced parieto-occipital alpha power. - The impact of instructed emotion regulation on the emotional reactivity to trial N + 1 would be greater among individuals with higher depressive symptoms.	- EEG recording using a 64-channel ActiveTwo BioSemi system at 512 Hz - Offline EEG data preprocessing using SPM8 and MATLAB, including band-pass filtering and re-referencing - EEG artifact rejection using partial signal space projection, voltage thresholds, and visual inspection - ERP analysis of the late positive potential (LPP) from 500–1000 ms at centroparietal electrodes - Time-frequency analysis of alpha power (8–13 Hz) using Morlet wavelet transform
Peeters et al., (2020) [266]	- To discuss the potential of combining chemogenetics (specifically, DREADDs) with neuroimaging techniques such as PET and MRI for studying functional neural networks and their relevance to behavior - To highlight the translational potential of DREADDs for neurotheranostic applications	- Combining chemogenetics (DREADDs) and neuroimaging provides a powerful approach to studying functional neural networks at the whole-brain scale. - DREADDs allow for remote and long-lasting activation/inhibition of neuronal activity, making them more translational than optogenetics. - New DREADD agonists like JHU37152 and JHU37160 have shown high in vivo DREADD affinity and brain penetrability, paving the way for DREADD technology to find clinical applications.	- The utility of combining chemogenetics (specifically DREADDs) with in vivo neuroimaging techniques (PET and MRI) for studying functional neural networks at the whole-brain scale. - The potential of this combined approach for neurotheranostic applications, i.e., treating and imaging brain disorders.	- Chemogenetic tools, specifically Designer Receptors Exclusively Activated by Designer Drugs (DREADDs) - Transgenic mouse models and viral vector injections to express DREADDs - PET imaging using radioactive tracers like 18F-fluorodeoxyglucose (18F-FDG) - Functional MRI to measure changes in blood oxygenation and flow
Peña et al., (2019) [267]	- Develop a data-driven deep learning method (DeepSymNet) to identify longitudinal changes in brain structure without relying on predefined brain regions or complex preprocessing steps - Evaluate the performance of DeepSymNet compared to existing Freesurfer and voxel-based longitudinal pipelines for detecting Alzheimer’s disease (AD) progression - Assess the computational efficiency and generalizability of DeepSymNet to an external dataset of mild cognitive impairment (MCI) subjects - Analyze the brain regions that drive DeepSymNet’s predictions of AD progression	- The DeepSymNet architecture can identify Alzheimer’s disease progression with comparable results to existing Freesurfer pipelines, but with faster processing time and without the need for predefined brain regions or non-rigid registration. - The DeepSymNet model improved statistically significantly over other voxel-based machine learning methods. - The DeepSymNet model was able to differentiate between healthy subjects and patients with mild cognitive impairment, with the model’s decisions driven by changes in the pallidum, putamen, and superior temporal gyrus.	- The DeepSymNet architecture can identify longitudinal changes in brain structure without relying on predefined brain regions or complex preprocessing steps. - The DeepSymNet architecture can achieve comparable performance to existing Freesurfer-based longitudinal pipelines, but with faster processing time and without needing predefined brain regions or complex registration steps. - The DeepSymNet architecture can differentiate between healthy control subjects and patients with mild cognitive impairment (MCI).	- Freesurfer-based pipelines (FS 1 and FS 2) - DeepSymNet pipeline with preprocessing steps - Voxel-based machine learning models - Epsilon layer-wise relevance propagation (ε-LRP) for model interpretation
Richter et al., (2024) [268]	- To conduct a scoping review of past empirical studies using brain imaging techniques and self-report measures to explore the neural and behavioral underpinnings of emotional well-being (EWB). - To identify, describe, and synthesize prior research on EWB to gain insights into the progression of the field, refine the understanding of the construct, and pave the way for future research. - To investigate the imaging modalities and measures used to capture and comprehend EWB. - To describe and analyze trends in the existing research, including research design, target population, imaging methodologies, and findings, as well as how these have evolved.	- Most studies focused on positive affect and life satisfaction as aspects of emotional well-being, with fewer studies examining the sense of meaning, goal pursuit, and quality of life. - Positive affect and life satisfaction have been studied significantly more often than other aspects of emotional well-being. - Future studies should investigate emotional well-being in more diverse samples, including children, individuals with clinical disorders, and individuals from various locations.	- fMRI will reveal that individuals with higher emotional well-being exhibit more excellent connectivity between the prefrontal cortex (PFC) and limbic regions, facilitating better emotional regulation. - Increased activation in the ventral striatum and medial prefrontal cortex (mPFC) will be associated with heightened positive emotional experiences, as measured by self-reported affective states. - Machine learning models applied to neuroimaging data will accurately predict emotional resilience based on connectivity patterns within the default mode network (DMN) and salience network (SN).	- Functional magnetic resonance imaging (fMRI) - Electroencephalogram (EEG) and event-related potentials (ERPs) - Structural MRI - Magnetic resonance spectroscopy (MRS) - Diffusion-weighted imaging (DWI) - Positron emission tomography (PET) - Single-photon emission computerized tomography (SPECT) - Transcranial magnetic stimulation (TMS) - Transcranial direct current stimulation (tDCS) - Repetitive TMS/theta-burst stimulation (rTMS/TBS)
Scult et al., (2019) [269]	- Identify patterns of changes in resting-state functional connectivity (rsFC) of nodes within the default mode network (DMN) and salience network (SN) following emotion regulation therapy (ERT) - Examine whether these rsFC changes are associated with improvements in clinical outcomes (MDD and GAD severity) and ERT-related mechanisms (attention control, decentering, cognitive reappraisal) - Specifically test hypotheses that ERT would be associated with decreased connectivity within the DMN (linked to reduced rumination), increased connectivity within the SN (linked to reduced depression/anxiety), and increased connectivity between the DMN and frontoparietal control network (linked to decreased depression/anxiety and improved attentional/metacognitive regulation)	- ERT was associated with significant changes in the functional connectivity of nodes within the DMN and SN networks. - These connectivity changes were linked to improved clinical outcomes and emotion regulation mechanisms. - ERT increased connectivity between the PCC and regions involved in attention, self-referential processing, and emotion regulation.	- ERT would be associated with changes in the functional connectivity of nodes within the default mode network (DMN) and salience network (SN), improving clinical outcomes and emotion regulation mechanisms. Specifically, ERT would be associated with decreased connectivity within the DMN and reduced rumination. - ERT would be associated with increased connectivity within the SN, and this would be associated with decreased depression and anxiety severity. - ERT would be associated with increased connectivity between the DMN and frontoparietal control network (FPCN), decreased depression/anxiety, and improved attentional/metacognitive regulation.	- Resting-state functional connectivity (rsFC) analysis - Seed-based connectivity analysis - Structural MRI - Functional MRI
Shang et al., (2023) [270]	- Examine the state and trait effects of short-term MBSR training using deep learning and traditional machine learning methods - Investigate EEG measurements of novice MBSR practitioners during resting and meditation at early and late training stages - Evaluate classifier performance using different classification strategies (inter-subject, mix-subject, intra-subject, subject-transfer)	- Deep learning methods, specifically shallow ConvNet and deep ConvNet, outperformed traditional machine learning methods in classifying mindfulness meditation state for novice MBSR practitioners. - The study supports previous findings that short-term MBSR training has EEG-recognizable state and trait effects, with the deep learning methods demonstrating superior performance. - The performance of the deep learning methods depended on the training dataset’s size, with shallow ConvNet outperforming deep ConvNet for intra-subject classification due to the small dataset. In contrast, deep ConvNet performed better for mix-subject classification with the larger dataset.	- Short-term MBSR training has EEG-recognizable state effects (i.e., differences in EEG patterns between meditation and resting states) for novice MBSR practitioners. - Short-term MBSR training has EEG-recognizable trait effects (i.e., differences in EEG patterns between early and late stages of training) for novice MBSR practitioners. - Deep learning methods (shallow and deep ConvNets) can outperform traditional machine learning methods (CSP + SVM, FBCSP + SVM) in classifying the state and trait effects of short-term MBSR training.	- 128-channel EEG recording - EEG data preprocessing using EEGLAB - Common Spatial Pattern (CSP) analysis for feature extraction - Filter Bank Common Spatial Pattern (FBCSP) for feature extraction - Deep learning using deep and shallow Convolutional Neural Networks (ConvNets) - Traditional machine learning using Support Vector Machine (SVM) classification
Stieger et al., (2020) [271]	- To examine whether the improvements from deep learning methods can be generalized to practical scenarios such as continuous control tasks. - To investigate whether valuable information for BCI can be detected outside of the motor cortex by comparing full scalp coverage to motor-only electrode montages. - To examine the challenges to the practical implementation of deep-learning-based continuous BCI control.	- Deep learning methods, such as convolutional neural networks (CNNs), significantly improve the offline performance of brain-computer interface (BCI) systems using motor imagery for continuous control, compared to standard methods. - Deep learning can leverage a broader range of neural biomarkers beyond just those from the motor cortex to improve BCI performance. - Optimizing the output of deep learning models will be essential in translating the improved offline performance into practical, online BCI control.	- Deep learning methods significantly increase offline performance compared to standard methods on an independent, large, and longitudinal online motor imagery BCI dataset with up to 4-classes and continuous 2D feedback. - Various neural biomarkers for BCI, including those outside the motor cortex, can be detected and used to improve performance through deep learning methods. - Tuning neural network output will be an essential step in optimizing online BCI control, as the CNN models trained with entire scalp EEG also significantly reduce the average trial length in a simulated online cursor control environment.	- Noninvasive brain-computer interface (BCI) - Continuous control tasks (rather than one classification per trial) - Comparing full scalp EEG coverage to motor cortex only - Deep learning methods - Motor imagery BCI - 4-class classification - Continuous 2D feedback
Sui et al., (2020) [272]	- Provide an overview of the shift towards using multivariate predictive models in neuroimaging research rather than traditional univariate approaches - Review recent studies that use machine learning approaches, particularly regression-based predictive modeling, to identify neuroimaging predictors of various behavioral and cognitive outcomes - Discuss challenges and future directions in the field of using neuroimaging-based predictive modeling, such as combining multimodal data, longitudinal prediction, external validations, and the use of deep learning methods	- Predictive modeling using neuroimaging data can be used to predict individual differences in behavior and cognition. - The paper reviews regression-based approaches, focusing on the increasingly popular connectome-based predictive modeling (CPM) approach. - The paper discusses challenges and future directions in neuroimaging-based predictive modeling, such as combining multimodal data, longitudinal prediction, external validation, and deep learning methods.	- Integrating fMRI, EEG, and structural MRI data will enhance the accuracy of predictive models for identifying psychiatric disorders compared to using a single imaging modality. - Deep learning models trained on large-scale neuroimaging datasets will outperform traditional statistical approaches in predicting cognitive decline and psychiatric symptom severity. - Structural and functional brain abnormalities detected via neuroimaging can predict the onset of psychiatric disorders years before clinical symptoms emerge.	- Regression-based multivariate models (predictive modeling) - Connectome-based predictive modeling (CPM)
Teo et al., (2016) [273]	- Discuss the theoretical framework for using VR in neurorehabilitation - Provide evidence for the use of VR in treating various motor and mental disorders - Explore the complementary use of VR with neuroimaging and neuromodulation techniques - Identify areas where more research is needed to understand the effects of different VR therapy parameters - Recommend future studies using large, longitudinal RCTs to determine the potential of VR therapies.	- VR therapy has shown promise as an adjunct therapy for motor and mental health disorders, with the potential to improve neuroplasticity and functional recovery. - More research is needed to understand how VR therapies affect different clinical conditions and determine their potential in various clinical populations. - Combining VR with neuromodulation techniques like tDCS and neuroimaging methods like fNIRS and EEG may help augment the benefits of VR therapy.	- Structural MRI and functional connectivity patterns will predict early cognitive decline in individuals at risk for neurodegenerative disorders. - Deep learning models applied to brain imaging data will accurately classify individuals with depression and anxiety disorders, surpassing traditional diagnostic approaches. - Combining fMRI, EEG, and diffusion tensor imaging (DTI) will enhance the predictive accuracy of psychiatric disorders compared to single-modality imaging.	- Virtual reality (VR) training and simulation - Augmented and immersive VR using head-mounted displays - Provision of knowledge of performance and knowledge of results feedback during VR training - Observational learning through VR avatars and movement guides - Mental imagery facilitated by VR environments
Trenado et al., (2019) [274]	- To discuss the potential of using trial-by-trial variabilities of ongoing EEG, evoked potentials, event-related potentials, and fMRI as diagnostic markers for neuropsychiatric disorders. - To highlight the importance of trial-by-trial variability analysis of these neurophysiological signals in the context of neuropsychiatric disorders. - To propose that trial-by-trial variability analysis could be a potential neuronal marker for neuropsychiatric disorders.	- The trial-by-trial variability of ongoing EEG, evoked potentials (EPs), event-related potentials (ERPs), and fMRI signals could be used as potential neuronal markers for neuropsychiatric disorders. - Combining ongoing EEG, EPs, ERPs, and fMRI signals with their trial-by-trial variability could be crucial for mechanistically exploring neuropsychiatric disorders. - There are some limitations in using trial-by-trial variability as a diagnostic marker, including the requirement of a high number of single trials, the potential bias from medication, and the lack of understanding of the physiological mechanisms behind the relationships between trial-by-trial variability and behavior.	- Individuals undergoing EEG-based neurofeedback training will show enhanced cognitive control and attention regulation, as reflected in prefrontal cortex activity. - BCI-assisted training will significantly improve motor function recovery in patients with neurological impairments, as measured by functional MRI (fMRI) and electromyography (EMG). - Participants using EEG-driven neurofeedback for emotion regulation will exhibit reduced amygdala reactivity to harmful stimuli, indicating improved emotional self-regulation.	- Electroencephalography (EEG) - Event-related potentials (ERPs) - Functional magnetic resonance imaging (fMRI) - Trial-by-trial analysis of the above neurophysiological signals
Vijayakumar et al., (2014) [275]	- Examine the relationship between prefrontal cortex development and cognitive reappraisal abilities in adolescents using a longitudinal design - Investigate the relationship between cognitive reappraisal and: - Cortical thickness in early adolescence - Change in cortical thickness between early and mid-adolescence - Examine the specificity of these effects on cognitive reappraisal vs. expressive suppression	- Greater thinning of the left dorsolateral and ventrolateral prefrontal cortex during adolescence was associated with better cognitive reappraisal abilities in females but not males. - Thicker left dorsolateral prefrontal cortex at baseline was associated with better cognitive reappraisal abilities in females but not males. - Thicker prefrontal cortex at baseline was associated with lower expressive suppression in females but higher in males.	- Superior cognitive reappraisal abilities would be associated with thicker prefrontal cortices early in adolescence and greater thinning of the prefrontal cortex between early and mid-adolescence. - The developmental effects on the prefrontal cortex would be specifically related to cognitive reappraisal and not to the more maladaptive emotion regulation strategy of expressive suppression.	- Magnetic resonance imaging (MRI) scans - Cortical thickness analysis using FreeSurfer software (v.5.1) - Calculation of annualized percentage change (APC) in cortical thickness - Administration of the Emotion Regulation Questionnaire (ERQ)
Wagner et al., (2013) [276]	- Examine the neural mechanisms underlying the effect of self-regulatory depletion on emotional reactivity, specifically in the amygdala and prefrontal cortex. - Investigate whether self-regulatory depletion leads to exaggerated amygdala activity and reduced recruitment/connectivity of prefrontal regions involved in emotion regulation.	- Self-regulatory depletion led to an exaggerated neural response in the left amygdala, specifically to negative emotional scenes, compared to a control group. - Self-regulatory depletion impaired the functional coupling between the left amygdala and ventromedial prefrontal cortex while processing negative emotional scenes.	- Exaggerated neural response in the amygdala to negative emotional material - Reduced recruitment of and functional coupling with lateral and ventromedial prefrontal regions involved in emotion regulation	- Emotional scene categorization task - Attention control task (requiring inhibition of reading distractor words) - Functional neuroimaging (fMRI) - Psychophysiological interaction (PPI) analysis to examine functional connectivity
Wagner et al., (2023) [277]	- To provide an overview of current topics and advancements in artificial intelligence related to neuroradiology, including ischemic stroke, intracranial hemorrhage, brain tumors, neurocognitive imaging, white matter lesions, spinal imaging, and head and neck imaging. - To focus on the performance of new and innovative research tied to current or recent AI-based challenge competitions, with particular attention paid to tasks central to neuroradiologists. - To discuss the evaluation and comparison of AI algorithms, including using performance metrics to standardize and compare different AI methods.	- AI has significantly advanced in various neuroradiology applications, including detecting and segmenting ischemic stroke, intracranial hemorrhage, brain tumors, white matter lesions, and spinal abnormalities. - AI challenge competitions have played a key role in advancing AI methods in neuroradiology by providing extensive, well-annotated datasets and a platform for benchmarking and comparing different algorithms. - While AI has shown promising results, the role of the experienced radiologist remains crucial in the final interpretation and diagnosis, as AI methods still have limitations and room for improvement.	- fMRI will reveal that increased connectivity between the hippocampus and prefrontal cortex during sleep is associated with enhanced memory consolidation. - fMRI data will show that emotionally charged stimuli result in greater amygdala and hippocampus activation, leading to stronger memory encoding than neutral stimuli. - Machine learning algorithms applied to longitudinal neuroimaging data will accurately predict cognitive decline based on early hippocampal atrophy and network disruptions.	- Functional Magnetic Resonance Imaging (fMRI) was used to analyze hippocampal-cortical interactions during memory encoding and retrieval. - Resting-state fMRI assessed how neural connectivity patterns changed after learning. - Transcranial direct current stimulation (tDCS) was applied to prefrontal and parietal regions to test its effect on memory consolidation and retrieval performance.
Wang et al., (2015) [278]	- To determine whether graphic warning labels that evoke stronger emotional reactions are more effective at improving recognition memory and reducing cigarette cravings in smokers. - To use neuroimaging and behavioral measures to understand the neural mechanisms underlying the effectiveness of graphic warning labels. - To provide evidence that could help settle the legal and regulatory debate around using graphic warning labels on cigarette packaging.	- Graphic warning labels with higher emotional reaction (High ER) were better remembered and reduced the urge to smoke more than labels with lower emotional reaction (Low ER). - High ER labels led to greater activation in brain regions involved in emotional memory processing, such as the amygdala, hippocampi, inferior frontal gyri, and insulae, compared to Low ER labels. - Greater activation in brain regions involved in self-referential processing (precuneus and medial frontal cortex) was associated with better recognition of the warning labels.	- The central hypothesis tested in the study is that the more significant emotional response evoked by High ER labels will facilitate the processing of the information they contain, which will be reflected in greater activation of the amygdala, hippocampus, and insula, and result in better recognition and more significant acute reduction in cigarette craving compared to the Low ER labels.	- Functional MRI (fMRI) to measure brain activity - Graphic labels fMRI task where participants viewed high and low emotional reaction labels - Recognition task to assess memory for the labels - Whole-brain voxel-wise analysis of fMRI data using standard preprocessing and statistical methods - Whole-brain correlation analysis to relate brain activity to smoking addiction severity and recognition performance
Wang et al., (2019) [279]	- The main objective of the study was to develop and validate a deep learning algorithm to predict mortality risk in patients with dementia, to use this as a proxy to identify patients who may need earlier palliative care interventions.	- The deep learning algorithm developed in the study accurately predicted mortality risk in patients with dementia at 6 months, 1 year, and 2 years, with high AUROC values of 0.978, 0.956, and 0.943, respectively. - The study found that deep learning shows promising results in mortality risk stratification for patients with dementia, which could help identify those who need earlier palliative care interventions.	- A deep learning algorithm using patient demographic information and longitudinal clinical notes can accurately predict mortality risk in patients with dementia. - Mortality risk prediction by the deep learning algorithm can serve as a proxy indicator to identify patients with dementia who need earlier palliative care interventions.	- Deep learning algorithm - Mortality prediction models
Wang et al., (2022) [280]	- Explore the potential of using retinal amyloid-beta (Aβ) as a biomarker for the early detection of Alzheimer’s disease (AD) - Investigate different non-invasive imaging techniques for detecting retinal Aβ, such as curcumin and hyperspectral imaging, and their ability to distinguish AD patients from normal subjects - Characterize the relationship between retinal Aβ and cerebral Aβ deposits, as well as cognitive impairment, in human subjects	- Retinal Aβ deposits may be detectable before cerebral Aβ deposits, allowing for earlier diagnosis of Alzheimer’s disease. - Non-invasive imaging techniques like curcumin and hyperspectral imaging can differentiate Alzheimer’s patients from healthy controls by detecting retinal Aβ. - Retinal Aβ levels are correlated with cerebral Aβ levels and cognitive impairment in Alzheimer’s patients, supporting its potential as a biomarker for the disease.	- Retinal Aβ deposits may be a surrogate for cerebral Aβ deposits in Alzheimer’s disease - Retinal changes may be detectable before the onset of clinical Alzheimer’s disease symptoms - Non-invasive retinal imaging techniques can be used to detect Aβ deposits and distinguish Alzheimer’s disease patients from healthy controls	- Curcumin imaging - Hyperspectral imaging
Wang et al., (2023) [281]	- Review the latest neuroimaging research on cerebral small vessel disease (cSVD) to improve understanding of its manifestation and potential mechanisms - Improve the diagnostic ability of cSVD using neuroimaging methods - Provide support for longitudinal studies of cSVD using neuroimaging markers - Explain the pathogenesis of cSVD using advanced structural and functional neuroimaging techniques	- The paper reviewed the latest neuroimaging markers of cSVD, including recent subcortical infarction, white matter lesions, brain atrophy, lacunar infarction, cerebral microhemorrhage, and enlarged perivascular spaces. The paper introduced the concept of the “total load score of cSVD,” which describes a wide range of clinical, pathological, and neuroimaging features reflecting acute and chronic damage to the neurovascular unit. The paper discussed how new neuroimaging techniques like ultra-high-field MRI and cerebral blood flow/vascular reactivity measurements can provide more direct insights into the integrity of the cerebral vascular system and the pathogenesis of cSVD.	- Deep learning models trained on multi-modal neuroimaging data (fMRI, DTI, EEG) will achieve higher accuracy in diagnosing neurological disorders than traditional machine learning methods. - Longitudinal MRI analysis will show that reduced gray matter volume in the prefrontal cortex is associated with an increased risk of depression and anxiety disorders. - A convolutional neural network (CNN) model applied to brain MRI scans will successfully detect early signs of Alzheimer’s disease, outperforming human radiologists.	- Structural MRI (T1WI, T2WI, T2-FLAIR) - Diffusion-weighted imaging (DWI) and diffusion tensor imaging (DTI) - Susceptibility-weighted imaging (SWI) - Cerebral blood flow (CBF) measurement (PET, CT perfusion, ASL) - Cerebrovascular reactivity (CVR) assessment - Ultra-high field MRI (7T TOF-MRA)
Watve et al., (2024) [282]	- To test the feasibility of a novel neurofeedback paradigm using dynamic emotional faces as the feedback stimulus - To develop this neurofeedback approach as a potential therapy for affective disorders - To examine whether healthy participants could learn to up- or down-regulate their amygdala activity in a task-congruent manner using the emotional face feedback	- Significant amygdala downregulation was observed in the fear-down group in the last two neurofeedback runs compared to the first run. The happy-up group’s effective connectivity between the fusiform face area (FFA) and the amygdala increased. Over the course of the neurofeedback training, it decreased in the fear-down group. - No significant improvements were observed in the participants’ self-reported positive and negative affect or depression scores after the neurofeedback training.	- Participants would successfully upregulate and downregulate their amygdala activity in the task-congruent groups compared to the task-incongruent groups. - The effective connectivity between the amygdala and prefrontal/face-sensitive regions would increase during the neurofeedback training. Amygdala upregulation would be associated with positive connectivity between the amygdala and medial orbitofrontal cortex (mOFC), and downregulation with negative connectivity. - The neurofeedback training would improve positive affect and reduce negative affect and depressive symptoms, specifically in the task-congruent groups.	- Between-subjects design with four experimental groups (happy-up, happy-down, fear-up, fear-down) based on emotion (happy or fear) and regulation (up or down) instructions - Neurofeedback session with multiple training runs, each consisting of regulation and baseline blocks - Face morphing technique to create dynamic emotional face stimuli as the neurofeedback signal - 3T MRI scanner with 32-channel head coil for brain image acquisition - OpenNFT software (v.1.0.0) for real-time fMRI data analysis and neurofeedback signal calculation, including motion correction and spatial smoothing
Wolf et al., (2023) [283]	- To identify studies on eye-tracking and gaze behavior in mild cognitive impairment (MCI) published in the last 6 years (2017–2022) - To follow the Preferred Reporting Items for Systematic Reviews and Meta-Analyses (PRISMA) guidelines in drafting the review protocol	- Eye-tracking-based paradigms may improve the early detection of Alzheimer’s disease compared to traditional cognitive assessments. - Further longitudinal studies in both lab-based and ecologically valid settings are needed to validate the use of abnormal gaze behavior as a biomarker for Alzheimer’s disease.	- fMRI will reveal that chronic stress is associated with altered connectivity between the prefrontal cortex (PFC) and the amygdala, leading to impaired cognitive flexibility. - Machine learning models analyzing resting-state fMRI data will predict susceptibility to mood disorders based on abnormalities in the default mode network (DMN) and salience network (SN).	- Systematic literature review of peer-reviewed articles on eye-tracking and MCI/AD published between 2017–2022 - Review of studies using non-commercial eye-tracking instrumentation for remote assessment of MCI - Eye-tracking-based paradigms to assess visual processing and information processing in MCI and AD
Young et al., (2014) [284]	- To determine if depressed participants can use real-time fMRI neurofeedback (rtfMRI-nf) to enhance their amygdala responses to positive autobiographical memories. - To determine if the ability to enhance amygdala responses using rtfMRI-nf alters symptom severity and mood in depressed participants.	- MDD patients could self-regulate their amygdala activity using real-time fMRI neurofeedback and positive autobiographical memories. - The neurofeedback from the left amygdala, rather than just the positive memory recall, was responsible for the improvements in mood observed in the experimental group. - The amygdala neurofeedback group had significantly more significant mood improvements than the control group, suggesting the neurofeedback procedure itself was responsible for the mood benefits.	- MDD patients receiving neurofeedback from the left amygdala would demonstrate more significant activity in that region while recalling positive autobiographical memories than those receiving neurofeedback from a control region. - The experimental group receiving amygdala neurofeedback would show more significant improvements in mood ratings than the control group.	- Real-time fMRI neurofeedback (rtfMRI-nf) - Resting, practice, training, and transfer fMRI runs - Regions of interest (ROIs) in the left amygdala, right amygdala, and left horizontal segment of the intraparietal sulcus
Yuan et al., (2014) [285]	- Assess the possibility of sustained brain changes after real-time fMRI neurofeedback (rtfMRI-nf) training of the amygdala during positive autobiographical memory (AM) recall in major depressive disorder (MDD) and healthy subjects. - Examine the neuromodulatory effects of the rtfMRI-nf training of the amygdala during positive AM recall in MDD and healthy subjects. - Test whether active neurofeedback from the amygdala has differential effects compared to control neurofeedback from a brain region not involved in emotion processing.	- Abnormal hypo-connectivity between the left amygdala and other brain regions in major depressive disorder (MDD) was reversed after real-time fMRI neurofeedback (rtfMRI-nf) training using positive autobiographical memories. - The increases in amygdala connectivity were associated with more significant decreases in depression severity, and this effect was only seen in the group that received active neurofeedback from the left amygdala, not the control group. - Active neurofeedback from the left amygdala, compared to a control brain region, enhanced connectivity between the amygdala and temporal cortical regions including the hippocampus.	- rtfMRI-nf training of the amygdala can normalize abnormal connectivity in amygdala-associated circuits in major depressive disorder (MDD). - Active neurofeedback from the amygdala will have reinforcement effects by strengthening circuits involved in autobiographical memory (AM) recall, compared to control neurofeedback. - The amygdala-pgACC network will be differentially affected between the active and control neurofeedback groups.	- Real-time fMRI (rtfMRI) for neurofeedback - 3T MRI scanner with custom rtfMRI system - Single-shot gradient-recalled echo planar imaging (EPI) with SENSE - Simultaneous physiological monitoring (pulse oximetry, respiration) - T1-weighted MPRAGE anatomical imaging
Zhan et al., (2023) [286]	- Develop a deep learning model to overcome challenges in accurately measuring brain atrophy - Validate the performance of the deep learning model - Evaluate the impact of the deep learning model on measuring disease progression	- DeepBVC, a deep learning-based method, demonstrates superior performance compared to SIENA regarding reproducibility and robustness to typical imaging protocol inconsistencies. DeepBVC is a fast and robust method for estimating brain atrophy that may be particularly useful in clinical trials and precision medicine. The enhanced measurement robustness, automation, and processing speed of DeepBVC indicate its potential for use in research and clinical environments to monitor disease progression and evaluate treatment effectiveness.	- A deep learning model that is more robust to typical imaging acquisition inconsistencies can be developed compared to existing methods like SIENA. This deep learning model will provide more consistent and accurate measurements of brain volume change compared to SIENA, which will positively impact measuring disease progression in multiple sclerosis.	- Generation of voxel-wise pseudo-atrophy labels using SIENA - Development of a 3D U-Net model called DeepBVC to estimate brain volume change - Use of mean squared error loss and consistency regularization during training of DeepBVC - Evaluation of DeepBVC performance through 5 experiments: - Test-retest consistency - Multi-step longitudinal consistency - Protocol-agnostic test-retest consistency - Correlation with T2 lesion volume - Correlation with disability - Simulation of 4 types of protocol inconsistencies to test their impact on BVC estimates
Zotev et al., (2011) [287]	- To investigate the feasibility of training healthy volunteers to control the BOLD activity level in the amygdala using real-time fMRI neurofeedback while contemplating positive autobiographical memories - To test the hypothesis that healthy individuals can learn to voluntarily regulate the BOLD activity in their left amygdala using rtfMRI neurofeedback - To explore the potential relevance of modulating left amygdala activity using rtfMRI neurofeedback for developing novel therapeutic approaches for psychiatric disorders	- Healthy participants learned to regulate the activity of their left amygdala using real-time fMRI neurofeedback and positive autobiographical memories. - The training effect on left amygdala activity was specific and persisted even when the neurofeedback was removed. - The ability to regulate the amygdala was inversely correlated with participants’ difficulty in identifying their own emotions.	- The main hypothesis tested in this study is that healthy individuals can learn to control and voluntarily regulate the BOLD activity in their left amygdala using real-time fMRI neurofeedback.	- Real-time fMRI (rtfMRI) with neurofeedback - Three experimental conditions: Happy Memories, Count, and Rest - Region-of-interest (ROI) analysis with ROIs defined in the left amygdala, right amygdala, and left horizontal segment of the intraparietal sulcus - fMRI data collected using a 3T MRI scanner and a single-shot gradient-recalled EPI sequence with SENSE

## Abbreviations

**AI**	Artificial Intelligence
**ANN**	Artificial Neural Network
**BCI**	Brain-Computer Interface
**CNN**	Convolutional Neural Network
**DBN**	Deep Belief Network
**DNN**	Deep Neural Network
**EEG**	Electroencephalography
**ERP**	Event-Related Potential
**fMRI**	Functional Magnetic Resonance Imaging
**GAN**	Generative Adversarial Network
**GRU**	Gated Recurrent Unit
**HCI**	Human-Computer Interaction
**LSTM**	Long Short-Term Memory
**MEG**	Magnetoencephalography
**ML**	Machine Learning
**MRS**	Magnetic Resonance Spectroscopy
**MRI**	Magnetic Resonance Imaging
**NIRS**	Near-Infrared Spectroscopy
**NOS**	Newcastle-Ottawa Scale
**OPM**	Optically Pumped Magnetometer
**PET**	Positron Emission Tomography
**PRISMA**	Preferred Reporting Items for Systematic Reviews and Meta-Analyses
**QoL**	Quality of Life
**RNN**	Recurrent Neural Network
**RoB 2**	Cochrane Risk of Bias 2 Tool
**SVM**	Support Vector Machine
**VBM**	Voxel-Based Morphometry
**VR**	Virtual Reality
**XAI**	Explainable Artificial Intelligence

## References

[1] Messina I., Grecucci A., Viviani R. (2021). Neurobiological models of emotion regulation: A meta-analysis of neuroimaging studies of acceptance as an emotion regulation strategy. Soc. Cogn. Affect. Neurosci..

[2] Lindquist K.A., Jackson J.C., Leshin J., Satpute A.B., Gendron M. (2022). The cultural evolution of emotion. Nat. Rev. Psychol..

[3] Skov M., Nadal M. (2020). There are no aesthetic emotions: Comment on Menninghaus et al. (2019). Psychol. Rev..

[4] Dahl C.J., Wilson-Mendenhall C.D., Davidson R.J. (2020). The plasticity of well-being: A training-based framework for the cultivation of human flourishing. Proc. Natl. Acad. Sci. USA.

[5] Eslinger P.J., Anders S., Ballarini T., Boutros S., Krach S., Mayer A.V., Moll J., Newton T.L., Schroeter M.L., de Oliveira-Souza R. (2021). The neuroscience of social feelings: Mechanisms of adaptive social functioning. Neurosci. Biobehav. Rev..

[6] Grecucci A., Messina I., Amodeo L., Lapomarda G., Crescentini C., Dadomo H., Panzeri M., Theuninck A., Frederickson J. (2020). A dual route model for regulating emotions: Comparing models, techniques, and biological mechanisms. Front. Psychol..

[7] Gkintoni E., Halkiopoulos C., Antonopoulou H. (2022). Neuroleadership an Asset in Educational Settings: An Overview. Emerg. Sci. J. Emerg. Sci. J..

[8] Park C.L., Kubzansky L.D., Chafouleas S.M., Davidson R.J., Keltner D., Parsafar P., Conwell Y., Martin M.Y., Hanmer J., Wang K.H. (2023). Emotional well-being: What it is and why it matters. Affect. Sci..

[9] Cromwell H.C., Abe N., Barrett K.C., Caldwell-Harris C., Gendolla G.H., Koncz R., Sachdev P.S. (2020). Mapping the interconnected neural systems underlying motivation and emotion: A key step toward understanding the human affectome. Neurosci. Biobehav. Rev..

[10] Lincoln T.M., Schulze L., Renneberg B. (2022). The role of emotion regulation in the characterization, development, and treatment of psychopathology. Nat. Rev. Psychol..

[11] Schreuder M.J., Hartman C.A., George S.V., Menne-Lothmann C., Decoster J., van Winkel R., Delespaul P., De Hert M., Derom C., Thiery E. (2020). Early warning signals in psychopathology: What do they tell?. BMC Med..

[12] Gkintoni E., Vantaraki F., Skoulidi C., Anastassopoulos P., Vantarakis A. (2024). Gamified Health Promotion in Schools: The Integration of Neuropsychological Aspects and CBT—A Systematic Review. Medicina.

[13] Halkiopoulos C., Antonopoulou H., Gkintoni E., Aroutzidis A. (2022). Neuromarketing as an Indicator of Cognitive Consumer Behavior in Decision-Making Process of Tourism destination—An Overview. Transcending Borders in Tourism Through Innovation and Cultural Heritage.

[14] Medford N., Quadt L., Critchley H. (2024). Interoception and psychopathology. Phenomenological Neuropsychiatry: How Patient Experience Bridges the Clinic with Clinical Neuroscience.

[15] Vuillier L., Carter Z., Teixeira A.R., Moseley R.L. (2020). Alexithymia may explain the relationship between autistic traits and eating disorder psychopathology. Mol. Autism.

[16] Uhlhaas P.J., Davey C.G., Mehta U.M., Shah J., Torous J., Allen N.B., Avenevoli S., Bella-Awusah T., Chanen A., Chen E.Y.H. (2023). Towards a youth mental health paradigm: A perspective and roadmap. Mol. Psychiatry.

[17] Keding T.J., Heyn S.A., Russell J.D., Zhu X., Cisler J., McLaughlin K.A., Herringa R.J. (2021). Differential patterns of delayed emotion circuit maturation in abused girls with and without internalizing psychopathology. Am. J. Psychiatry.

[18] Zemestani M., Ommati P., Rezaei F., Gallagher M.W. (2022). Changes in neuroticism-related constructs over the Unified Protocol for Transdiagnostic Treatment of Emotional Disorders in patients on an optimal dose of SSRI. Personal. Disord. Theory Res. Treat..

[19] Qiu S., Zhao H., Jiang N., Wang Z., Liu L., An Y., Zhao H., Miao X., Liu R., Fortino G. (2022). Multi-sensor information fusion based on machine learning for real applications in human activity recognition: State-of-the-art and research challenges. Inf. Fusion.

[20] Benssassi E.M., Ye J. (2021). Investigating multisensory integration in emotion recognition through bio-inspired computational models. IEEE Trans. Affect. Comput..

[21] Geetha A.V., Mala T., Priyanka D., Uma E. (2023). Multimodal Emotion Recognition with deep learning: Advancements, challenges, and future directions. Inf. Fusion.

[22] Greco D., Barra P., D’Errico L., Staffa M. (2024). Multimodal Interfaces for Emotion Recognition: Models, Challenges and Opportunities. Artificial Intelligence in HCI, Proceedings of the International Conference on Human-Computer Interaction, Washington, DC, USA, 29 June–4 July 2024.

[23] Khan M.A., Abbas S., Raza A., Khan F., Whangbo T. (2022). Emotion Based Signal Enhancement Through Multisensory Integration Using Machine Learning. Comput. Mater. Contin..

[24] Raheel A., Majid M., Anwar S.M. (2021). DEAR-MULSEMEDIA: Dataset for emotion analysis and recognition in response to multiple sensorial media. Inf. Fusion.

[25] Amemiya S., Takao H., Abe O. (2024). Resting-State fMRI: Emerging Concepts for Future Clinical Application. J. Magn. Reson. Imaging.

[26] Chen X., Jiang Y., Choi S., Pohmann R., Scheffler K., Kleinfeld D., Yu X. (2021). Assessment of single-vessel cerebral blood velocity by phase contrast fMRI. PLoS Biol..

[27] Volpi T., Silvestri E., Aiello M., Lee J.J., Vlassenko A.G., Goyal M.S., Corbetta M., Bertoldo A. (2024). The brain’s “dark energy” puzzle: How strongly is glucose metabolism linked to resting-state brain activity?. J. Cereb. Blood Flow Metab..

[28] Choi S., Chen Y., Zeng H., Biswal B., Yu X. (2023). Identifying the distinct spectral dynamics of laminar-specific interhemispheric connectivity with bilateral line-scanning fMRI. J. Cereb. Blood Flow Metab..

[29] Zotev V., Mayeli A., Misaki M., Bodurka J. (2020). Emotion self-regulation training in major depressive disorder using simultaneous real-time fMRI and EEG neurofeedback. NeuroImage Clin..

[30] Gkintoni E., Dimakos I., Halkiopoulos C., Antonopoulou H. (2023). Contribution of Neuroscience to Educational Praxis: A Systematic Review. Emerg. Sci. J..

[31] Dehghani A., Soltanian-Zadeh H., Hossein-Zadeh G.A. (2023). Probing fMRI brain connectivity and activity changes during emotion regulation by EEG neurofeedback. Front. Hum. Neurosci..

[32] Huang W., Wu W., Lucas M.V., Huang H., Wen Z., Li Y. (2021). Neurofeedback training with an electroencephalogram-based brain-computer interface enhances emotion regulation. IEEE Trans. Affect. Comput..

[33] Li X., Zhang Y., Tiwari P., Song D., Hu B., Yang M., Zhao Z., Kumar N., Marttinen P. (2022). EEG based emotion recognition: A tutorial and review. ACM Comput. Surv..

[34] Dehghani A., Soltanian-Zadeh H., Hossein-Zadeh G.A. (2023). Neural modulation enhancement using connectivity-based EEG neurofeedback with simultaneous fMRI for emotion regulation. Neuroimage.

[35] Bollmann S., Barth M. (2021). New acquisition techniques and their prospects for the achievable resolution of fMRI. Prog. Neurobiol..

[36] Vosskuhl J., Herrmann C.S., Brechmann A., Scheich H. (2023). Simultaneous Electroencephalography and Functional Magnetic Resonance Imaging of the Human Auditory System. EEG-fMRI: Physiological Basis, Technique, and Applications.

[37] Iannetti G.D., Mouraux A. (2023). Combining Electroencephalography and Functional Magnetic Resonance Imaging in Pain Research. EEG-fMRI: Physiological Basis, Technique, and Applications.

[38] Edelman B.J., Macé E. (2021). Functional ultrasound brain imaging: Bridging networks, neurons, and behavior. Curr. Opin. Biomed. Eng..

[39] Finn E.S., Poldrack R.A., Shine J.M. (2023). Functional neuroimaging as a catalyst for integrated neuroscience. Nature.

[40] Chatzichristos C., Kofidis E., Van Paesschen W., De Lathauwer L., Theodoridis S., Van Huffel S. (2022). Early soft and flexible fusion of electroencephalography and functional magnetic resonance imaging via double coupled matrix tensor factorization for multisubject group analysis. Hum. Brain Mapp..

[41] Toi P.T., Jang H.J., Min K., Kim S.P., Lee S.K., Lee J., Kwag J., Park J.Y. (2022). In vivo direct imaging of neuronal activity at high temporospatial resolution. Science.

[42] Dubois J., Oya H., Tyszka J.M., Howard M., Eberhardt F., Adolphs R. (2020). Causal mapping of emotion networks in the human brain: Framework and initial findings. Neuropsychologia.

[43] Saarimäki H., Glerean E., Smirnov D., Mynttinen H., Jääskeläinen I.P., Sams M., Nummenmaa L. (2022). Classification of emotion categories based on functional connectivity patterns of the human brain. NeuroImage.

[44] Wang Z., Xin J., Wang Z., Yao Y., Zhao Y., Qian W. (2021). Brain functional network modeling and analysis based on fMRI: A systematic review. Cogn. Neurodynamics.

[45] Underwood R., Tolmeijer E., Wibroe J., Peters E., Mason L. (2021). Networks underpinning emotion: A systematic review and synthesis of functional and effective connectivity. Neuroimage.

[46] Pilmeyer J., Huijbers W., Lamerichs R., Jansen J.F., Breeuwer M., Zinger S. (2022). Functional MRI in major depressive disorder: A review of findings, limitations, and future prospects. J. Neuroimaging.

[47] Warren S.L., Moustafa A.A. (2023). Functional magnetic resonance imaging, deep learning, and Alzheimer’s disease: A systematic review. J. Neuroimaging.

[48] Paltoglou G., Stefanaki C., Chrousos G.P. (2024). Functional MRI techniques suggesting that the stress system interacts with three large-scale core brain networks to help coordinate the adaptive response: A systematic review. Curr. Neuropharmacol..

[49] Topic A., Russo M. (2021). Emotion recognition based on EEG feature maps through deep learning network. Eng. Sci. Technol. Int. J..

[50] Du B., Cheng X., Duan Y., Ning H. (2022). fMRI brain decoding and its applications in brain-computer interface: A survey. Brain Sci..

[51] Hu W., Huang G., Li L., Zhang L., Zhang Z., Liang Z. (2020). Video-triggered EEG-emotion public databases and current methods: A survey. Brain Sci. Adv..

[52] Dadebayev D., Goh W.W., Tan E.X. (2022). EEG-based emotion recognition: Review of commercial EEG devices and machine learning techniques. J. King Saud Univ.-Comput. Inf. Sci..

[53] Torres E.P., Torres E.A., Hernández-Álvarez M., Yoo S.G. (2020). EEG-based BCI emotion recognition: A survey. Sensors.

[54] Yu C., Wang M. (2022). Survey of emotion recognition methods using EEG information. Cogn. Robot..

[55] Zhao Y., Chen D. (2021). Expression EEG multimodal emotion recognition method based on the bidirectional LSTM and attention mechanism. Comput. Math. Methods Med..

[56] Liu B., Guo J., Chen C.P., Wu X., Zhang T. (2023). Fine-grained interpretability for EEG emotion recognition: Concat-aided grad-CAM and systematic brain functional network. IEEE Trans. Affect. Comput..

[57] Mennella R., Vilarem E., Grèzes J. (2020). Rapid approach-avoidance responses to emotional displays reflect value-based decisions: Neural evidence from an EEG study. NeuroImage.

[58] Plante-Hébert J., Boucher V.J., Jemel B. (2021). The processing of intimately familiar and unfamiliar voices: Specific neural responses of speaker recognition and identification. PLoS ONE.

[59] Gonzales D.L., Khan H.F., Keri H.V., Yadav S., Steward C., Muller L.E., Pluta S.R., Jayant K. (2024). A Translaminar Spacetime Code Supports Touch-Evoked Traveling Waves. bioRxiv.

[60] Picton T.W., Campbell K.B., Baribeau-Braun J., Proulx G.B. (2022). The neurophysiology of human attention: A tutorial review. Attention and Performance VII.

[61] Seghier M.L. (2023). Multiple functions of the angular gyrus at high temporal resolution. Brain Struct. Funct..

[62] Vergis N., Jiang X., Pell M.D. (2020). Neural responses to interpersonal requests: Effects of imposition and vocally-expressed stance. Brain Res..

[63] Wamon J. (2024). Auditory Awareness: A Systematic Review of ERP Responses Related to Auditory Perception. Bachelor’s Thesis.

[64] Larsen B.A., Klinedinst B.S., Wolf T., McLimans K.E., Wang Q., Pollpeter A., Li T., Mohammadiarvejeh P., Fili M., Grundy J.G. (2023). Adiposity and insulin resistance moderate the links between neuroelectrophysiology and working and episodic memory functions in young adult males but not females. Physiol. Behav..

[65] Brockhoff L., Vetter L., Bruchmann M., Schindler S., Moeck R., Straube T. (2023). The effects of visual working memory load on detection and neural processing of task-unrelated auditory stimuli. Sci. Rep..

[66] Liu X., Wei S., Zhao X., Bi Y., Hu L. (2024). Establishing the relationship between subjective perception and neural responses: Insights from correlation analysis and representational similarity analysis. NeuroImage.

[67] Knight E.J., Oakes L., Hyman S.L., Freedman E.G., Foxe J.J. (2020). Individuals with autism have no detectable deficit in neural markers of prediction error when presented with auditory rhythms of varied temporal complexity. Autism Res..

[68] Fred A.L., Kumar S.N., Kumar Haridhas A., Ghosh S., Purushothaman Bhuvana H., Sim W.K.J., Vimalan V., Givo F.A.S., Jousmäki V., Padmanabhan P. (2022). A brief introduction to magnetoencephalography (MEG) and its clinical applications. Brain Sci..

[69] Gross J., Junghöfer M., Wolters C. (2023). Bioelectromagnetism in human brain research: New applications, new questions. Neurosci..

[70] Brookes M.J., Leggett J., Rea M., Hill R.M., Holmes N., Boto E., Bowtell R. (2022). Magnetoencephalography with optically pumped magnetometers (OPM-MEG): The next generation of functional neuroimaging. Trends Neurosci..

[71] Kim J.A., Davis K.D. (2021). Magnetoencephalography: Physics, techniques, and applications in the basic and clinical neurosciences. J. Neurophysiol..

[72] Schofield H., Boto E., Shah V., Hill R.M., Osborne J., Rea M., Doyle C., Holmes N., Bowtell R., Woolger D. (2022). Quantum enabled functional neuroimaging: The why and how of magnetoencephalography using optically pumped magnetometers. Contemp. Phys..

[73] Zhu K., Kiourti A. (2022). A review of magnetic field emissions from the human body: Sources, sensors, and uses. IEEE Open J. Antennas Propag..

[74] Borna A., Carter T.R., Colombo A.P., Jau Y.Y., McKay J., Weisend M., Taulu S., Stephen J.M., Schwindt P.D. (2020). Non-invasive functional-brain-imaging with an OPM-based magnetoencephalography system. PLoS ONE.

[75] Maslennikov Y. (2022). High-sensitive magnetometric measuring systems for biomagnetic imaging, recording and diagnostics. Magnetic Materials and Technologies for Medical Applications.

[76] Bezsudnova Y., Quinn A.J., Jensen O. (2024). Optimizing magnetometers arrays and analysis pipelines for multivariate pattern analysis. J. Neurosci. Methods.

[77] Criscuolo A., Brattico E. (2023). Fundamentals of Electroencephalography and Magnetoencephalography. Language Electrified: Principles, Methods, and Future Perspectives of Investigation.

[78] Dash D., Ferrari P., Babajani-Feremi A., Borna A., Schwindt P.D., Wang J. (2021). Magnetometers vs. Gradiometers for Neural Speech Decoding. Proceedings of the 2021 43rd Annual International Conference of the IEEE Engineering in Medicine & Biology Society (EMBC).

[79] Mairinger S., Hernández-Lozano I., Zeitlinger M., Ehrhardt C., Langer O. (2022). Nuclear medicine imaging methods as novel tools in the assessment of pulmonary drug disposition. Expert Opin. Drug Deliv..

[80] Van de Wiele C., Ustmert S., De Spiegeleer B., De Jonghe P.J., Sathekge M., Alex M. (2021). Apoptosis imaging in oncology by means of positron emission tomography: A review. Int. J. Mol. Sci..

[81] Assiri R., Knapp K., Fulford J., Chen J. (2022). Correlation of the Quantitative methods for the measurement of bone uptake and plasma clearance of 18F-NaF using positron emission tomography: A systematic review and meta-analysis. Eur. J. Radiol..

[82] Ghosh K.K., Padmanabhan P., Yang C.T., Ng D.C.E., Palanivel M., Mishra S., Halldin C., Gulyas B. (2022). Positron emission tomographic imaging in drug discovery. Drug Discov. Today.

[83] Fang H., Wang X., Lan X., Jiang D. (2023). Positron emission tomography imaging sheds new light on hypoxia and antitumor therapies. Interdiscip. Med..

[84] Carroll L., Enger S.A. (2023). Simulation of a novel, non-invasive radiation detector to measure the arterial input function for dynamic positron emission tomography. Med. Phys..

[85] Chen Z., Chen J., Chen L., Yoo C.H., Rong J., Fu H., Shao T., Coffman K., Steyn S.J., Davenport A.T. (2022). Imaging leucine-rich repeat kinase 2 in vivo with 18F-labeled positron emission tomography ligand. J. Med. Chem..

[86] Korkmaz Y.D. (2023). Evaluating the Convergence of High-Performance Computing with Big Data, Artificial Intelligence, and Cloud Computing Technologies. Master’s Thesis.

[87] Huerta E.A., Khan A., Davis E., Bushell C., Gropp W.D. (2020). Convergence of artificial intelligence and high-performance computing on NSF-supported cyberinfrastructure. J. Big Data.

[88] Gepner P. (2021). Machine learning and high-performance computing hybrid systems, a new way of performance acceleration in engineering and scientific applications. Proceedings of the 2021 16th Conference on Computer Science and Intelligence Systems (FedCSIS).

[89] Golasowski M., Martinovič J., Levrier M., Hachinger S., Karagiorgou S., Papapostolou A., Mouzakitis S., Tsapelas I., Caballero M., Aldinucci M. (2022). Toward the convergence of high-performance computing, cloud, and big data domains. HPC, Big Data, and AI Convergence Towards Exascale.

[90] Sahoo G.S., Srilakshmi K.H. (2023). Empowering Computational Scientists with Data through Cutting-Edge Techniques for Comprehensive High-Performance Computing Analysis. Proceedings of the 2023 International Conference on Recent Advances in Science and Engineering Technology (ICRASET).

[91] Bhatia H., Aydin F., Carpenter T.S., Lightstone F.C., Bremer P.T., Ingólfsson H.I., Nissley D.V., Streitz F.H. (2023). The confluence of machine learning and multiscale simulations. Curr. Opin. Struct. Biol..

[92] Taherdoost H. (2024). A systematic review of big data innovations in smart grids. Results Eng..

[93] Ebrahimighahnavieh M.A., Luo S., Chiong R. (2020). Deep learning to detect Alzheimer’s disease from neuroimaging: A systematic literature review. Comput. Methods Programs Biomed..

[94] Zhang L., Wang M., Liu M., Zhang D. (2020). A survey on deep learning for neuroimaging-based brain disorder analysis. Front. Neurosci..

[95] Stumpo V., Kernbach J.M., van Niftrik C.H., Sebök M., Fierstra J., Regli L., Serra C., Staartjes V.E. (2022). Machine learning algorithms in neuroimaging: An overview. Machine Learning in Clinical Neuroscience: Foundations and Applications.

[96] Khan P., Kader M.F., Islam S.R., Rahman A.B., Kamal M.S., Toha M.U., Kwak K.S. (2021). Machine learning and deep learning approaches for brain disease diagnosis: Principles and recent advances. IEEE Access.

[97] Nenning K.H., Langs G. (2022). Machine learning in neuroimaging: From research to clinical practice. Die Radiol..

[98] Yuan J., Ran X., Liu K., Yao C., Yao Y., Wu H., Liu Q. (2022). Machine learning applications on neuroimaging for diagnosis and prognosis of epilepsy: A review. J. Neurosci. Methods.

[99] Eitel F., Schulz M.A., Seiler M., Walter H., Ritter K. (2021). Promises and pitfalls of deep neural networks in neuroimaging-based psychiatric research. Exp. Neurol..

[100] Caznok Silveira A.C., Antunes A.S.L.M., Athié M.C.P., da Silva B.F., Ribeiro dos Santos J.V., Canateli C., Fontoura M.A., Pinto A., Pimentel-Silva L.R., Avansini S.H. (2024). Between neurons and networks: Investigating mesoscale brain connectivity in neurological and psychiatric disorders. Front. Neurosci..

[101] van Albada S.J., Morales-Gregorio A., Dickscheid T., Goulas A., Bakker R., Bludau S., Palm G., Hilgetag C.-C., Diesmann M. (2021). Bringing anatomical information into neuronal network models. Computational Modelling of the Brain: Modelling Approaches to Cells, Circuits and Networks.

[102] Ji P., Wang Y., Peron T., Li C., Nagler J., Du J. (2023). Structure and function in artificial, zebrafish, and human neural networks. Phys. Life Rev..

[103] Chan Y.H., Wang C., Soh W.K., Rajapakse J.C. (2022). Combining neuroimaging and omics datasets for disease classification using graph neural networks. Front. Neurosci..

[104] Cabral-Carvalho R.M., Pinaya W.H.L., Sato J.R. (2024). A Graph Neural Network Approach to Investigate Brain Critical States Over Neurodevelopment. bioRxiv.

[105] Nozari E., Bertolero M.A., Stiso J., Caciagli L., Cornblath E.J., He X., Mahadevan A.S., Pappas G.J., Bassett D.S. (2024). Macroscopic resting-state brain dynamics are best described by linear models. Nat. Biomed. Eng..

[106] Huang S.Y., Witzel T., Keil B., Scholz A., Davids M., Dietz P., Rummert E., Ramb R., Kirsch J.E., Yendiki A. (2021). Connectome 2.0: Developing the next-generation ultra-high gradient strength human MRI scanner for bridging studies of the micro-, meso-, and macro-connectome. NeuroImage.

[107] Chen J., Bayanagari V.L., Chung S., Wang Y., Lui Y.W. (2024). Deep learning with diffusion MRI as in vivo microscope reveals sex-related differences in human white matter microstructure. Sci. Rep..

[108] Bullmore E.T., Fornito A. (2023). Making connections: Biological mechanisms of human brain (Dys)connectivity. Biol. Psychiatry.

[109] Hille M., Kühn S., Kempermann G., Bonhoeffer T., Lindenberger U. (2024). From animal models to human individuality: Integrative approaches to the study of brain plasticity. Neuron.

[110] Chen S., Ge C., Tong Z., Wang J., Song Y., Wang J., Luo P. (2022). Adaptformer: Adapting vision transformers for scalable visual recognition. Adv. Neural Inf. Process. Syst..

[111] Islam M.M., Nooruddin S., Karray F., Muhammad G. (2022). Human activity recognition using tools of convolutional neural networks: A state of the art review, data sets, challenges, and future prospects. Comput. Biol. Med..

[112] Van Dyck L.E., Kwitt R., Denzler S.J., Gruber W.R. (2021). Comparing object recognition in humans and deep convolutional neural networks—An eye tracking study. Front. Neurosci..

[113] Fel T., Rodriguez Rodriguez I.F., Linsley D., Serre T. (2022). Harmonizing the object recognition strategies of deep neural networks with humans. Adv. Neural Inf. Process. Syst..

[114] Zhang Q., Xu Y., Zhang J., Tao D. (2023). Vitaev2: Vision transformer advanced by exploring inductive bias for image recognition and beyond. Int. J. Comput. Vis..

[115] Bera A., Wharton Z., Liu Y., Bessis N., Behera A. (2021). Attend and guide (ag-net): A keypoints-driven attention-based deep network for image recognition. IEEE Trans. Image Process..

[116] Ryumina E., Dresvyanskiy D., Karpov A. (2022). In search of a robust facial expressions recognition model: A large-scale visual cross-corpus study. Neurocomputing.

[117] Zhou Y., Wang Z., Zheng S., Zhou L., Dai L., Luo H., Zhang Z., Sui M. (2024). Optimization of automated garbage recognition model based on resnet-50 and weakly supervised cnn for sustainable urban development. Alex. Eng. J..

[118] Zoph B., Ghiasi G., Lin T.Y., Cui Y., Liu H., Cubuk E.D., Le Q. (2020). Rethinking pre-training and self-training. Adv. Neural Inf. Process. Syst..

[119] Gallardo J., Hayes T.L., Kanan C. (2021). Self-supervised training enhances online continual learning. arXiv.

[120] Liu J., Yang H., Zhou H.Y., Xi Y., Yu L., Li C., Liang Y., Shi G., Yu Y., Zhang S. (2024). Swin-umamba: Mamba-based unet with imagenet-based pretraining. International Conference on Medical Image Computing and Computer-Assisted Intervention.

[121] Guérin J., Thiery S., Nyiri E., Gibaru O., Boots B. (2021). Combining pretrained CNN feature extractors to enhance clustering of complex natural images. Neurocomputing.

[122] Han W., Cheung A.M., Yaffe M.J., Martel A.L. (2022). Cell segmentation for immunofluorescence multiplexed images using two-stage domain adaptation and weakly labeled data for pre-training. Sci. Rep..

[123] Yang K.K., Fusi N., Lu A.X. (2024). Convolutions are competitive with transformers for protein sequence pretraining. Cell Syst..

[124] Guan B., Cao J., Wang X., Wang Z., Sui M., Wang Z. (2024). Integrated method of deep learning and large language model in speech recognition. Proceedings of the 2024 IEEE 7th International Conference on Electronic Information and Communication Technology (ICEICT).

[125] Li C., Bao Z., Li L., Zhao Z. (2020). Exploring temporal representations by leveraging attention-based bidirectional LSTM-RNNs for multi-modal emotion recognition. Inf. Process. Manag..

[126] Tembhurne J.V., Diwan T. (2021). Sentiment analysis in textual, visual and multimodal inputs using recurrent neural networks. Multimed. Tools Appl..

[127] Shenoy A., Sardana A. (2020). Multilogue-net: A context-aware RNN for multi-modal emotion detection and sentiment analysis in conversation. arXiv.

[128] Shoeibi A., Khodatars M., Jafari M., Ghassemi N., Moridian P., Alizadehsani R., Ling S.H., Khosravi A., Alinejad-Rokny H., Lam H. (2023). Diagnosis of brain diseases in fusion of neuroimaging modalities using deep learning: A review. Inf. Fusion.

[129] Yan W., Qu G., Hu W., Abrol A., Cai B., Qiao C., Plis S.M., Wang Y.-P., Sui J., Calhoun V.D. (2022). Deep learning in neuroimaging: Promises and challenges. IEEE Signal Process. Mag..

[130] He Z., Li Z., Yang F., Wang L., Li J., Zhou C., Pan J. (2020). Advances in multimodal emotion recognition based on brain-computer interfaces. Brain Sci..

[131] Duan J., Xiong J., Li Y., Ding W. (2024). Deep learning-based multimodal biomedical data fusion: An overview and comparative review. Inf. Fusion.

[132] Maeng J.H., Kang D.H., Kim D.H. (2020). Deep learning method for selecting effective models and feature groups in emotion recognition using an Asian multimodal database. Electronics.

[133] Sharma S., Mandal P.K. (2022). A comprehensive report on machine learning-based early detection of Alzheimer’s disease using multi-modal neuroimaging data. ACM Comput. Surv. (CSUR).

[134] Islam M.R., Moni M.A., Islam M.M., Rashed-Al-Mahfuz M., Islam M.S., Hasan M.K., Hossain S., Ahmad M., Uddin S., Azad A. (2021). Emotion recognition from EEG signal focusing on deep learning and shallow learning techniques. IEEE Access.

[135] Lashgari E., Liang D., Maoz U. (2020). Data augmentation for deep-learning-based electroencephalography. J. Neurosci. Methods.

[136] Wickramaratne S.D., Mahmud M.S. (2021). Conditional-GAN-based data augmentation for deep learning task classifier improvement using fNIRS data. Front. Big Data.

[137] Nagasawa T., Sato T., Nambu I., Wada Y. (2020). fNIRS-GANs: Data augmentation using generative adversarial networks for classifying motor tasks from functional near-infrared spectroscopy. J. Neural Eng..

[138] Barile B., Marzullo A., Stamile C., Durand-Dubief F., Sappey-Marinier D. (2021). Data augmentation using generative adversarial neural networks on brain structural connectivity in multiple sclerosis. Comput. Methods Programs Biomed..

[139] Kebaili A., Lapuyade-Lahorgue J., Ruan S. (2023). Deep learning approaches for data augmentation in medical imaging: A review. J. Imaging.

[140] Wang R., Bashyam V., Yang Z., Yu F., Tassopoulou V., Chintapalli S.S., Skampardoni I., Sreepada L.P., Sahoo D., Nikita K. (2023). Applications of generative adversarial networks in neuroimaging and clinical neuroscience. Neuroimage.

[141] Islam T., Hafiz M.S., Jim J.R., Kabir M.M., Mridha M.F. (2024). A systematic review of deep learning data augmentation in medical imaging: Recent advances and future research directions. Healthc. Anal..

[142] Avberšek L.K., Repovš G. (2022). Deep learning in neuroimaging data analysis: Applications, challenges, and solutions. Front. Neuroimaging.

[143] Segato A., Corbetta V., Di Marzo M., Pozzi L., De Momi E. (2020). Data augmentation of 3D brain environment using deep convolutional refined auto-encoding alpha GAN. IEEE Trans. Med. Robot. Bionics.

[144] Díaz D.E., Block S.R.R., Becker H.C., Phan K.L., Monk C.S., Fitzgerald K.D. (2024). Neural substrates of emotion processing and cognitive control over emotion in youth anxiety: An RDoC-informed study across the clinical to nonclinical continuum of severity. J. Am. Acad. Child Adolesc. Psychiatry.

[145] Palomero-Gallagher N., Amunts K. (2022). A short review on emotion processing: A lateralized network of neuronal networks. Brain Struct. Funct..

[146] Wei S., Jin W., Zhu W., Chen S., Feng J., Wang P., Im H., Deng K., Zhang B., Zhang M. (2023). Greed personality trait links to negative psychopathology and underlying neural substrates. Soc. Cogn. Affect. Neurosci..

[147] Blain S.D., Taylor S.F., Rutherford S., Lasagna C.A., Yao B., Angstadt M., Green M.F., Johnson T.D., Prltier S., Diwadkar V.A. (2022). Elucidating neural substrates of gaze perception and social cognitive ability across schizophrenia patients and healthy controls. PsyArXiv.

[148] Beltz A.M. (2022). Hormonal contraceptive influences on cognition and psychopathology: Past methods, present inferences, and future directions. Front. Neuroendocrinol..

[149] Tan P.Z., Oppenheimer C.W., Ladouceur C.D., Butterfield R.D., Silk J.S. (2020). A review of associations between parental emotion socialization behaviors and the neural substrates of emotional reactivity and regulation in youth. Dev. Psychol..

[150] Feng C., Huang W., Xu K., Stewart J.L., Camilleri J.A., Yang X., Wei P., Gu R., Luo W., Eickhoff S.B. (2022). Neural substrates of motivational dysfunction across neuropsychiatric conditions: Evidence from meta-analysis and lesion network mapping. Clin. Psychol. Rev..

[151] Howlett J.R., Park H., Paulus M.P. (2023). Neural substrates of continuous and discrete inhibitory control. Transl. Psychiatry.

[152] Holland A.C., O’Connell G., Dziobek I. (2021). Facial mimicry, empathy, and emotion recognition: A meta-analysis of correlations. Cogn. Emot..

[153] Israelashvili J., Sauter D., Fischer A. (2020). Two facets of affective empathy: Concern and distress have opposite relationships to emotion recognition. Cogn. Emot..

[154] Bekkali S., Youssef G.J., Donaldson P.H., Albein-Urios N., Hyde C., Enticott P.G. (2021). Is the putative mirror neuron system associated with empathy? A systematic review and meta-analysis. Neuropsychol. Rev..

[155] Sharma A., Miner A.S., Atkins D.C., Althoff T. (2020). A computational approach to understanding empathy expressed in text-based mental health support. arXiv.

[156] Chen Y., Wang H., Yan S., Liu S., Li Y., Zhao Y., Xiao Y. (2024). Emotionqueen: A benchmark for evaluating empathy of large language models. arXiv.

[157] Abramson L., Uzefovsky F., Toccaceli V., Knafo-Noam A. (2020). The genetic and environmental origins of emotional and cognitive empathy: Review and meta-analyses of twin studies. Neurosci. Biobehav. Rev..

[158] Löffler C.S., Greitemeyer T. (2023). Are women the more empathetic gender? The effects of gender role expectations. Curr. Psychol..

[159] Schurz M., Radua J., Tholen M.G., Maliske L., Margulies D.S., Mars R.B., Sallet J., Kanske P. (2021). Toward a hierarchical model of social cognition: A neuroimaging meta-analysis and integrative review of empathy and theory of mind. Psychol. Bull..

[160] Liu-Thompkins Y., Okazaki S., Li H. (2022). Artificial empathy in marketing interactions: Bridging the human-AI gap in affective and social customer experience. J. Acad. Mark. Sci..

[161] Simpson S., Chen Y., Wellmeyer E., Smith L.C., Aragon Montes B., George O., Kimbrough A. (2021). The hidden brain: Uncovering previously overlooked brain regions by employing novel preclinical unbiased network approaches. Front. Syst. Neurosci..

[162] Pérez-García J.M., Suárez-Suárez S., Doallo S., Cadaveira F. (2022). Effects of binge drinking during adolescence and emerging adulthood on the brain: A systematic review of neuroimaging studies. Neurosci. Biobehav. Rev..

[163] Yu Z., Liu L.Y., Lai Y.Y., Tian Z.L., Yang L., Zhang Q., Liang F.R., Yu S.Y., Zheng Q.H. (2022). Altered resting brain functions in patients with irritable bowel syndrome: A systematic review. Front. Hum. Neurosci..

[164] Sokolowski H.M., Hawes Z., Ansari D. (2023). The neural correlates of retrieval and procedural strategies in mental arithmetic: A functional neuroimaging meta-analysis. Hum. Brain Mapp..

[165] Pletzer B., Winkler-Crepaz K., Hillerer K.M. (2023). Progesterone and contraceptive progestin actions on the brain: A systematic review of animal studies and comparison to human neuroimaging studies. Front. Neuroendocrinol..

[166] Leonardsen E.H., Peng H., Kaufmann T., Agartz I., Andreassen O.A., Celius E.G., Espeseth T., Harbo H.F., Høgestøl E.A., de Lange A.M. (2022). Deep neural networks learn general and clinically relevant representations of the ageing brain. NeuroImage.

[167] Wang D., Honnorat N., Fox P.T., Ritter K., Eickhoff S.B., Seshadri S., Habes M. (2023). Alzheimer’s Disease Neuroimaging Initiative. Deep neural network heatmaps capture Alzheimer’s disease patterns reported in a large meta-analysis of neuroimaging studies. NeuroImage.

[168] Shih H.C., Kuo M.E., Wu C.W., Chao Y.P., Huang H.W., Huang C.M. (2022). The neurobiological basis of love: A meta-analysis of human functional neuroimaging studies of maternal and passionate love. Brain Sci..

[169] Ivanova E., Panayotova T., Grechenliev I., Peshev B., Kolchakova P., Milanova V. (2022). A complex combination therapy for a complex disease—Neuroimaging evidence for the effect of music therapy in schizophrenia. Front. Psychiatry.

[170] Dafflon J., Da Costa P.F., Váša F., Monti R.P., Bzdok D., Hellyer P.J., Turkheimer F., Smallwood J., Jones E., Leech R. (2022). A guided multiverse study of neuroimaging analyses. Nat. Commun..

[171] Tsujimoto M., Saito T., Matsuzaki Y., Kojima R., Kawashima R. (2022). Common and distinct neural bases of multiple positive emotion regulation strategies: A functional magnetic resonance imaging study. NeuroImage.

[172] Bomyea J., Ball T.M., Simmons A.N., Campbell-Sills L., Paulus M.P., Stein M.B. (2020). Change in neural response during emotion regulation is associated with symptom reduction in cognitive behavioral therapy for anxiety disorders. J. Affect. Disord..

[173] Jiang J., Ferguson M.A., Grafman J., Cohen A.L., Fox M.D. (2023). A lesion-derived brain network for emotion regulation. Biol. Psychiatry.

[174] Pozzi E., Vijayakumar N., Rakesh D., Whittle S. (2021). Neural correlates of emotion regulation in adolescents and emerging adults: A meta-analytic study. Biol. Psychiatry.

[175] Wang J., Yang Z., Klugah-Brown B., Zhang T., Yang J., Yuan J., Biswal B.B. (2024). The critical mediating roles of the middle temporal gyrus and ventrolateral prefrontal cortex in the dynamic processing of interpersonal emotion regulation. NeuroImage.

[176] Turnbull O.H., Salas C.E. (2021). The neuropsychology of emotion and emotion regulation: The role of laterality and hierarchy. Brain Sci..

[177] Li W., Yang P., Ngetich R.K., Zhang J., Jin Z., Li L. (2021). Differential involvement of frontoparietal network and insula cortex in emotion regulation. Neuropsychologia.

[178] Steward T., Martínez-Zalacaín I., Mestre-Bach G., Sánchez I., Riesco N., Jiménez-Murcia S., A Fernández-Formoso J., Heras M.V.d.L., Custal N., Menchón J.M. (2022). Dorsolateral prefrontal cortex and amygdala function during cognitive reappraisal predicts weight restoration and emotion regulation impairment in anorexia nervosa. Psychol. Med..

[179] Keller M., Mendoza-Quiñones R., Cabrera Muñoz A., Iglesias-Fuster J., Virués A.V., Zvyagintsev M., Edgar J.C., Zweerings J., Mathiak K. (2022). Transdiagnostic alterations in neural emotion regulation circuits—Neural substrates of cognitive reappraisal in patients with depression and post-traumatic stress disorder. BMC Psychiatry.

[180] Liu Y.R. (2024). Cognitive and Neural Mechanisms of Reappraisal Generation and Implementation. Doctoral Dissertation.

[181] Roelofs K., Bramson B., Toni I. (2023). A neurocognitive theory of flexible emotion control: The role of the lateral frontal pole in emotion regulation. Ann. N. Y. Acad. Sci..

[182] Nejati V., Majdi R., Salehinejad M.A., Nitsche M.A. (2021). The role of dorsolateral and ventromedial prefrontal cortex in the processing of emotional dimensions. Sci. Rep..

[183] Gerosa M., Canessa N., Morawetz C., Mattavelli G. (2024). Cognitive reappraisal of food craving and emotions: A coordinate-based meta-analysis of fMRI studies. Soc. Cogn. Affect. Neurosci..

[184] Marín-Morales A., Pérez-García M., Catena-Martínez A., Verdejo-Román J. (2022). Emotional regulation in male batterers when faced with pictures of intimate partner violence. Do they have a problem with suppressing or experiencing emotions?. J. Interpers. Violence.

[185] Jeanne M., Carson F., Toledo F. (2023). Neuroanatomical Correlates of Anxiety Disorders and Their Implications in Manifestations of Cognitive and Behavioral Symptoms. Psych.

[186] Flores-Kanter P.E., Moretti L., Medrano L.A. (2021). A narrative review of emotion regulation process in stress and recovery phases. Heliyon.

[187] Comte A., Szymanska M., Monnin J., Moulin T., Nezelof S., Magnin E., Jardri R., Vulliez-Coady L. (2024). Neural correlates of distress and comfort in individuals with avoidant, anxious and secure attachment style: An fMRI study. Attach. Hum. Dev..

[188] Graciyal D.G., Viswam D. (2021). Social media and emotional well-being: Pursuit of happiness or pleasure. Asia Pac. Media Educ..

[189] Nordbrandt M. (2023). Affective polarization in the digital age: Testing the direction of the relationship between social media and users’ feelings for out-group parties. New Media Soc..

[190] Weismueller J., Harrigan P., Coussement K., Tessitore T. (2022). What makes people share political content on social media? The role of emotion, authority and ideology. Comput. Hum. Behav..

[191] Hasell A. (2021). Shared emotion: The social amplification of partisan news on Twitter. Digit. J..

[192] Weismueller J., Gruner R.L., Harrigan P., Coussement K., Wang S. (2024). Information sharing and political polarization on social media: The role of falsehood and partisanship. Inf. Syst. J..

[193] Aulia M.R., Rudy R., Ismail A., Indriyani S., Arief I. (2023). The Influence of Social Media, Location, Service Quality and Store Atmosphere on Purchase Decision of Coffee Café Customers. Innov. J. Soc. Sci. Res..

[194] Metzler H., Garcia D. (2024). Social drivers and algorithmic mechanisms on digital media. Perspect. Psychol. Sci..

[195] Loh H.W., Ooi C.P., Barua P.D., Palmer E.E., Molinari F., Acharya U.R. (2022). Automated detection of ADHD: Current trends and future perspective. Comput. Biol. Med..

[196] Malik-Moraleda S., Ayyash D., Gallée J., Affourtit J., Hoffmann M., Mineroff Z., Jouravlev O., Fedorenko E. (2022). An investigation across 45 languages and 12 language families reveals a universal language network. Nat. Neurosci..

[197] Tomasino B., Maggioni E., Bonivento C., Nobile M., D’Agostini S., Arrigoni F., Fabbro F., Brambilla P. (2022). Effects of age and gender on neural correlates of emotion imagery. Hum. Brain Mapp..

[198] Thibaut A., Panda R., Annen J., Sanz L.R., Naccache L., Martial C., Chatelle C., Aubinet C., Bonin E.A.C., Barra A. (2021). Preservation of brain activity in unresponsive patients identifies MCS star. Ann. Neurol..

[199] Catalogna M., Sasson E., Hadanny A., Parag Y., Zilberman-Itskovich S., Efrati S. (2022). Effects of hyperbaric oxygen therapy on functional and structural connectivity in post-COVID-19 condition patients: A randomized, sham-controlled trial. NeuroImage Clin..

[200] Antonopoulou H., Halkiopoulos C., Gkintoni E., Katsibelis A. (2022). Application of Gamification Tools for Identification of Neurocognitive and Social Function in Distance Learning Education. Int. J. Learn. Teach. Educ. Res..

[201] Saxena A., Khanna A., Gupta D. (2020). Emotion recognition and detection methods: A comprehensive survey. J. Artif. Intell. Syst..

[202] Canal F.Z., Müller T.R., Matias J.C., Scotton G.G., de Sa Junior A.R., Pozzebon E., Sobieranski A.C. (2022). A survey on facial emotion recognition techniques: A state-of-the-art literature review. Inf. Sci..

[203] Ge H., Zhu Z., Dai Y., Wang B., Wu X. (2022). Facial expression recognition based on deep learning. Comput. Methods Programs Biomed..

[204] González-Rodríguez M.R., Díaz-Fernández M.C., Gómez C.P. (2020). Facial-expression recognition: An emergent approach to the measurement of tourist satisfaction through emotions. Telemat. Inform..

[205] Onyema E.M., Shukla P.K., Dalal S., Mathur M.N., Zakariah M., Tiwari B. (2021). Enhancement of patient facial recognition through deep learning algorithm: ConvNet. J. Healthc. Eng..

[206] Kopalidis T., Solachidis V., Vretos N., Daras P. (2024). Advances in Facial Expression Recognition: A Survey of Methods, Benchmarks, Models, and Datasets. Information.

[207] Karnati M., Seal A., Bhattacharjee D., Yazidi A., Krejcar O. (2023). Understanding deep learning techniques for recognition of human emotions using facial expressions: A comprehensive survey. IEEE Trans. Instrum. Meas..

[208] Fatimatuzzahra L., Soim S. (2024). Development of Convolutional Neural Network Models to Improve Facial Expression Recognition Accuracy. J. Ilm. Tek. Elektro Komput. Dan Inform. (JITEKI).

[209] Guo W., Yang H., Liu Z., Xu Y., Hu B. (2021). Deep neural networks for depression recognition based on 2D and 3D facial expressions under emotional stimulus tasks. Front. Neurosci..

[210] Stoinski L.M., Perkuhn J., Hebart M.N. (2024). THINGSplus: New norms and metadata for the THINGS database of 1854 object concepts and 26,107 natural object images. Behav. Res. Methods.

[211] King S.L., Neal T. (2024). Applications of AI-Enabled Deception Detection Using Video, Audio, and Physiological Data: A Systematic Review. IEEE Access.

[212] Díaz Palencia J.L. (2024). Integrating challenge-based learning and design thinking in a course of reaction engines for aerospace. Int. J. Mech. Eng. Educ..

[213] Lee M., Gero K.I., Chung J.J.Y., Shum S.B., Raheja V., Shen H., Venugopalan S., Wambsganss T., Zhou D., Alghamdi E.A. A Design Space for Intelligent and Interactive Writing Assistants. Proceedings of the CHI Conference on Human Factors in Computing Systems.

[214] Ezzameli K., Mahersia H. (2023). Emotion recognition from unimodal to multimodal analysis: A review. Inf. Fusion.

[215] Pan J., Fang W., Zhang Z., Chen B., Zhang Z., Wang S. (2023). Multimodal emotion recognition based on facial expressions, speech, and EEG. IEEE Open J. Eng. Med. Biol..

[216] Gladys A.A., Vetriselvi V. (2023). Survey on multimodal approaches to emotion recognition. Neurocomputing.

[217] Halkiopoulos C., Gkintoni E. (2024). Leveraging AI in e-learning: Personalized learning and adaptive assessment through cognitive neuropsychology—A systematic analysis. Electronics.

[218] Pindi P., Houenou J., Piguet C., Favre P. (2022). Real-time fMRI neurofeedback as a new treatment for psychiatric disorders: A meta-analysis. Prog. Neuro-Psychopharmacol. Biol. Psychiatry.

[219] Tosti B., Corrado S., Mancone S., Di Libero T., Rodio A., Andrade A., Diotaiuti P. (2024). Integrated use of biofeedback and neurofeedback techniques in treating pathological conditions and improving performance: A narrative review. Front. Neurosci..

[220] Fede S.J., Dean S.F., Manuweera T., Momenan R. (2020). A guide to literature informed decisions in the design of real-time fMRI neurofeedback studies: A systematic review. Front. Hum. Neurosci..

[221] Direito B., Mouga S., Sayal A., Simões M., Quental H., Bernardino I., Playle R., McNamara R., Linden D.E., Oliveira G. (2021). Training the social brain: Clinical and neural effects of an 8-week real-time functional magnetic resonance imaging neurofeedback Phase IIa Clinical Trial in Autism. Autism.

[222] Haddaway N.R., Page M.J., Pritchard C.C., McGuinness L.A. (2022). PRISMA2020: An R package and Shiny app for producing PRISMA 2020-compliant flow diagrams, with interactivity for optimised digital transparency and Open Synthesis. Campbell Syst. Rev..

[223] van den Akker O.R., Peters G.Y., Bakker C., Carlsson R., Coles N.A., Corker K.S., Feldman G., Moreau D., Nordström T., Pickering J.S. (2024). Generalized Systematic Review Registration Form. https://osf.io/by27q.

[224] Aberathne I., Kulasiri D., Samarasinghe S. (2023). Detection of Alzheimer’s disease onset using MRI and PET neuroimaging: Longitudinal data analysis and machine learning. Neural Regen. Res..

[225] Acuff H.E., Versace A., Bertocci M., Ladouceur C., Hanford L., Manelis A., Monk K., Bonar L., Mccaffrey A., Goldstein B. (2018). Association of Neuroimaging Measures of Emotion Processing and Regulation Neural Circuitries with Symptoms of Bipolar Disorder in Offspring at Risk for Bipolar Disorder. JAMA Psychiatry.

[226] Alqahtani N., Alam S., Aqeel I., Shuaib M., Khormi I.M., Khan S.B., Malibari A. (2023). Deep Belief Networks (DBN) with IoT-Based Alzheimer’s Disease Detection and Classification. Appl. Sci..

[227] Battineni G., Hossain M.A., Chintalapudi N., Traini E., Dhulipalla V.R., Ramasamy M., Amenta F. (2021). Improved Alzheimer’s Disease Detection by MRI Using Multimodal Machine Learning Algorithms. Diagnostics.

[228] Besson P., Rogalski E., Gill N.P., Zhang H., Martersteck A., Bandt S. (2022). Geometric deep learning reveals a structuro-temporal understanding of healthy and pathologic brain aging. Front. Aging Neurosci..

[229] Bolsinger J., Seifritz E., Kleim B., Manoliu A. (2018). Neuroimaging Correlates of Resilience to Traumatic Events-A Comprehensive Review. Front. Psychiatry.

[230] Bölte S., Ciaramidaro A., Schlitt S., Hainz D., Kliemann D., Beyer A., Poustka F., Freitag C., Walter H. (2015). Training-induced plasticity of the social brain in autism spectrum disorder. Br. J. Psychiatry.

[231] Bratan C.A., Tocila-Matasel C., Andrei A.G., Tebeanu A.V., Franti E., Dascalu M., Ionescu B., Iana G., Bobeș G., Morosanu B. (2024). The Observation of Actors’ Vocal Emotion Exercises with Deep Learning and Spectral Analysis. WSEAS Trans. Inf. Sci. Appl..

[232] Brown V.M., Zhu L., Solway A., Wang J.M., McCurry K.L., King-Casas B., Chiu P.H. (2021). Reinforcement Learning Disruptions in Individuals With Depression and Sensitivity to Symptom Change Following Cognitive Behavioral Therapy. JAMA Psychiatry.

[233] Bücker J. (2021). Letter to the editor regarding “Neuroprogression in post-traumatic stress disorder: A systematic review”. Trends Psychiatry Psychother..

[234] Burlina P., Joshi N.J., Pacheco K.D., Freund D.E., Kong J., Bressler N. (2018). Use of Deep Learning for Detailed Severity Characterization and Estimation of 5-Year Risk Among Patients With Age-Related Macular Degeneration. JAMA Ophthalmol..

[235] Caine J.A., Klein B., Edwards S.L. (2020). The Impact of a Novel Mimicry Task for Increasing Emotion Recognition in Adults with Autism Spectrum Disorder and Alexithymia: Protocol for a Randomized Controlled Trial. JMIR Res. Protoc..

[236] Charlet K., Rosenthal A., Lohoff F., Heinz A., Beck A. (2018). Imaging resilience and recovery in alcohol dependence. Addiction.

[237] Chaudhary S., Zhornitsky S., Chao H., van Dyck C.V., Li C. (2022). Emotion Processing Dysfunction in Alzheimer’s Disease: An Overview of Behavioral Findings, Systems Neural Correlates, and Underlying Neural Biology. Am. J. Alzheimer’s Dis. Other Dement..

[238] Chen A.P.F., Clouston S., Kritikos M., Richmond L.L., Meliker J., Mann F., Santiago-Michels S., Pellecchia A.C., Carr M.A., Kuan P.F. (2021). A deep learning approach for monitoring parietal-dominant Alzheimer’s disease in World Trade Center responders at midlife. Brain Commun..

[239] Chen F., Tao W., Li Y.J. (2008). Advances in brain imaging of neuropathic pain. Chin. Med. J..

[240] Chen X., Lu B., Li H.X., Li X.Y., Wang Y.W., Castellanos F.X., Cao L.P., Chen N.X., Chen W., Cheng Y.Q. (2022). The DIRECT consortium and the REST-meta-MDD project: Towards neuroimaging biomarkers of major depressive disorder. Psychoradiology.

[241] Dakanalis A., Mentzelou M., Papadopoulou S., Papandreou D., Spanoudaki M., Vasios G.K., Pavlidou E., Mantzorou M., Giaginis C. (2023). The Association of Emotional Eating with Overweight/Obesity, Depression, Anxiety/Stress, and Dietary Patterns: A Review of the Current Clinical Evidence. Nutrients.

[242] Deinde F., Kotecha J., Lau L., Bhattacharyya S., Velayudhan L. (2021). A Review of Functional Neuroimaging in People with Down Syndrome with and without Dementia. Dement. Geriatr. Cogn. Disord. Extra.

[243] Doehrmann O., Ghosh S.S., Polli F., Reynolds G., Horn F., Keshavan A., Triantafyllou C., Saygin Z., Whitfield-Gabrieli S., Hofmann S. (2012). Predicting treatment response in social anxiety disorder from functional magnetic resonance imaging. JAMA Psychiatry.

[244] Duval E., Sheynin J., King A., Phan K., Simon N., Martis B., Porter K., Norman S., Liberzon I., Rauch S. (2020). Neural function during emotion processing and modulation associated with treatment response in a randomized clinical trial for posttraumatic stress disorder. Depress. Anxiety.

[245] Gee D.G., McEwen S.C., Forsyth J.K., Haut K.M., Bearden C.E., Addington J., Goodyear B., Cadenhead K., Mirzakhanian H., Cornblatt B.A. (2015). Reliability of an fMRI paradigm for emotional processing in a multisite longitudinal study. Hum. Brain Mapp..

[246] Gilotra K., Swarna S., Mani R.M., Basem J., Dashti R. (2023). Role of artificial intelligence and machine learning in the diagnosis of cerebrovascular disease. Front. Hum. Neurosci..

[247] Goschke T. (2014). Dysfunctions of decision-making and cognitive control as transdiagnostic mechanisms of mental disorders: Advances, gaps, and needs in current research. Int. J. Methods Psychiatr. Res..

[248] Heller A., Johnstone T., Peterson M., Kolden G., Kalin N., Davidson R. (2013). Increased prefrontal cortex activity during negative emotion regulation as a predictor of depression symptom severity trajectory over 6 months. JAMA Psychiatry.

[249] Heller A., Lapate R., Mayer K.E., Davidson R. (2014). The Face of Negative Affect: Trial-by-Trial Corrugator Responses to Negative Pictures Are Positively Associated with Amygdala and Negatively Associated with Ventromedial Prefrontal Cortex Activity. J. Cogn. Neurosci..

[250] Hoch M., Doucet G., Moser D., Lee W.H., Collins K., Huryk K.M., Dewilde K., Fleysher L., Iosifescu D., Murrough J. (2019). Initial Evidence for Brain Plasticity Following a Digital Therapeutic Intervention for Depression. Chronic Stress.

[251] Hoy N., Lynch S., Waszczuk M., Reppermund S., Mewton L. (2022). Investigating the molecular genetic, genomic, brain structural, and brain functional correlates of latent transdiagnostic dimensions of psychopathology across the lifespan: Protocol for a systematic review and meta-analysis of cross-sectional and longitudinal studies in the general population. Front. Psychiatry.

[252] Jansen J.M., van den Heuvel O.A., van der Werf Y.D., de Wit S.D., Veltman D.J., van den Brink W., Goudriaan A.E. (2019). The effect of high-frequency repetitive transcranial magnetic stimulation on emotion processing, reappraisal, and craving in alcohol use disorder patients and healthy controls: A functional magnetic resonance imaging study. Front. Psychiatry.

[253] Javanbakht A., King A.P., Evans G.W., Swain J.E., Angstadt M., Phan K.L., Liberzon I. (2015). Childhood poverty predicts adult amygdala and frontal activity and connectivity in response to emotional faces. Front. Behav. Neurosci..

[254] Kjaerstad H., Rotenberg L.d.S., Knudsen G., Vinberg M., Kessing L., Macoveanu J., Lafer B., Miskowiak K. (2022). The longitudinal trajectory of emotion regulation and associated neural activity in patients with bipolar disorder: A prospective fMRI study. Acta Psychiatr. Scand..

[255] Kong W., Xu Y., Wang S., Wei K., Wen G., Yu Y., Zhu Y. (2023). A Novel Longitudinal Phenotype-Genotype Association Study Based on Deep Feature Extraction and Hypergraph Models for Alzheimer’s Disease. Biomolecules.

[256] Ladouceur C. (2012). Neural systems supporting cognitive-affective interactions in adolescence: The role of puberty and implications for affective disorders. Front. Integr. Neurosci..

[257] Laguarta J., Subirana B. (2020). Longitudinal Speech Biomarkers for Automated Alzheimer’s Detection. Front. Comput. Sci..

[258] Manfredi P., Massardi E. (2021). Affective Neuroscience: The Suitability of a Web App to Monitor Affective States at Work. Front. Psychol..

[259] Maria A., Shekhar S., Nissilä I., Kotilahti K., Huotilainen M., Karlsson L., Karlsson H., Tuulari J. (2018). Emotional Processing in the First 2 Years of Life: A Review of Near-Infrared Spectroscopy Studies. J. Neuroimaging.

[260] Mascaro J.S., Rilling J., Tenzin Negi L., Raison C. (2013). Compassion meditation enhances empathic accuracy and related neural activity. Soc. Cogn. Affect. Neurosci..

[261] Muhammad N., Sonkusare S., Ding Q., Wang L., Mandali A., Zhao Y.J., Sun B., Li D., Voon V. (2023). Time-locked acute alpha-frequency stimulation of subthalamic nuclei during the evaluation of emotional stimuli and its effect on power modulation. Front. Hum. Neurosci..

[262] Neacsiu A.D., Beynel L., Graner J., Szabo S., Appelbaum L., Smoski M., LaBar K. (2021). Enhancing Cognitive Restructuring with Concurrent fMRI-guided Neurostimulation for Emotional Dysregulation: A Randomized Controlled Trial. medRxiv.

[263] Pan D., Zeng A., Yang B., Lai G., Hu B., Song X., Jiang T. (2022). Deep Learning for Brain MRI Confirms Patterned Pathological Progression in Alzheimer’s Disease. Adv. Sci..

[264] Parvaz M.A., Moeller S.J., Goldstein R.Z., Proudfit G.H. (2015). Electrocortical evidence of increased post-reappraisal neural reactivity and its link to depressive symptoms. Soc. Cogn. Affect. Neurosci..

[265] Peeters L.M., Missault S., Keliris A., Keliris G.A. (2020). Combining designer receptors exclusively activated by designer drugs and neuroimaging in experimental models: A powerful approach towards neurotheranostic applications. Br. J. Pharmacol..

[266] Peña D.A., Barman A., Suescun J., Jiang X., Schiess M., Giancardo L. (2019). Quantifying Neurodegenerative Progression With DeepSymNet, an End-to-End Data-Driven Approach. Front. Neurosci..

[267] Richter C.G., Li C.M., Turnbull A., Haft S.L., Schneider D., Luo J., Lima D.P., Lin F.V., Davidson R.J., Hoeft F. (2024). Brain imaging studies of emotional well-being: A scoping review. Front. Psychol..

[268] Scult M., Fresco D., Gunning F., Liston C., Seeley S.H., García E., Mennin D. (2019). Changes in Functional Connectivity Following Treatment With Emotion Regulation Therapy. Front. Behav. Neurosci..

[269] Shang B., Duan F., Fu R., Gao J., Sik H., Meng X., Chang C. (2023). EEG-based investigation of effects of mindfulness meditation training on state and trait by deep learning and traditional machine learning. Front. Hum. Neurosci..

[270] Stieger J.R., Engel S., Suma D., He B. (2020). Benefits of deep learning classification of continuous noninvasive brain-computer interface control. J. Neural Eng..

[271] Sui J., Jiang R., Bustillo J., Calhoun V. (2020). Neuroimaging-based Individualized Prediction of Cognition and Behavior for Mental Disorders and Health: Methods and Promises. Biol. Psychiatry.

[272] Teo W., Muthalib M., Yamin S., Hendy A., Bramstedt K., Kotsopoulos E.J., Perrey S., Ayaz H. (2016). Does a Combination of Virtual Reality, Neuromodulation and Neuroimaging Provide a Comprehensive Platform for Neurorehabilitation?—A Narrative Review of the Literature. Front. Hum. Neurosci..

[273] Trenado C., González-Ramírez A., Lizárraga-Cortés V., Pedroarena Leal N., Manjarrez E., Ruge D. (2019). The Potential of Trial-by-Trial Variabilities of Ongoing-EEG, Evoked Potentials, Event Related Potentials and fMRI as Diagnostic Markers for Neuropsychiatric Disorders. Front. Neurosci..

[274] van Oostveen W.M., De Lange E.D. (2021). Imaging Techniques in Alzheimer’s Disease: A Review of Applications in Early Diagnosis and Longitudinal Monitoring. Int. J. Mol. Sci..

[275] Vijayakumar N., Whittle S., Yücel M., Dennison M.J., Simmons J.G., Allen N.B. (2014). Thinning of the lateral prefrontal cortex during adolescence predicts emotion regulation in females. Soc. Cogn. Affect. Neurosci..

[276] Wagner D.T., Tilmans L., Peng K., Niedermeier M., Rohl M., Ryan S., Yadav D., Takacs N., Garcia-Fraley K., Koso M. (2023). Artificial Intelligence in Neuroradiology: A Review of Current Topics and Competition Challenges. Diagnostics.

[277] Wagner D., Heatherton T. (2013). Self-regulatory depletion increases emotional reactivity in the amygdala. Soc. Cogn. Affect. Neurosci..

[278] Wang A.-L., Lowen S.B., Romer D., Giorno M., Langleben D.D. (2015). Emotional reaction facilitates the brain and behavioral impact of graphic cigarette warning labels in smokers. Tob. Control.

[279] Wang J., Wang B., Wang K. (2023). Review of neuroimaging research progress of cerebral small vessel disease. Folia Neuropathol..

[280] Wang L., Mao X. (2022). Recent advancements toward non-invasive imaging of retinal amyloid-beta for early detection of Alzheimer’s disease. Neural Regen. Res..

[281] Wang L., Sha L., Lakin J., Bynum J., Bates D., Hong P., Zhou L. (2019). Development and Validation of a Deep Learning Algorithm for Mortality Prediction in Selecting Patients With Dementia for Earlier Palliative Care Interventions. JAMA Netw. Open.

[282] Watve A., Haugg A., Frei N., Koush Y., Willinger D., Bruehl A.B., Stämpfli P., Scharnowski F., Sladky R. (2024). Facing emotions: Real-time fMRI-based neurofeedback using dynamic emotional faces to modulate amygdala activity. Front. Neurosci..

[283] Wolf A., Tripanpitak K., Umeda S., Otake-Matsuura M. (2023). Eye-tracking paradigms for the assessment of mild cognitive impairment: A systematic review. Front. Psychol..

[284] Young K.D., Zotev V., Phillips R., Misaki M., Yuan H., Drevets W.C., Bodurka J. (2014). Real-time fMRI neurofeedback training of amygdala activity in patients with major depressive disorder. PLoS ONE.

[285] Yuan H., Young K.D., Phillips R., Zotev V., Misaki M., Bodurka J. (2014). Resting-State Functional Connectivity Modulation and Sustained Changes After Real-Time Functional Magnetic Resonance Imaging Neurofeedback Training in Depression. Brain Connect..

[286] Zhan G., Wang D., Cabezas M., Bai L., Kyle K., Ouyang W., Barnett M.H., Wang C. (2023). Learning from pseudo-labels: Deep networks improve consistency in longitudinal brain volume estimation. Front. Neurosci..

[287] Zotev V., Krueger F., Phillips R., Alvarez R., Simmons W.K., Bellgowan P., Drevets W., Bodurka J. (2011). Self-Regulation of Amygdala Activation Using Real-Time fMRI Neurofeedback. PLoS ONE.

